# Modulation of cytokeratin and cytokine/chemokine expression following influenza virus infection of differentiated human tonsillar epithelial cells

**DOI:** 10.1128/jvi.01460-24

**Published:** 2025-01-10

**Authors:** S. Scott Perry, David C. Brice, Ahmed Atef Sakr, Ahmed Kandeil, Jennifer DeBeauchamp, Mohamed Ghonim, Jeremy Jones, Lance Miller, Kasi Vegesana, Jeremy Chase Crawford, Deanna M. Langfitt, Lisa Kercher, Hossam A. Abdelsamed, Robert G. Webster, Paul G. Thomas, Richard J. Webby, Faten A. Okda

**Affiliations:** 1 Department of Bone Marrow Transplantation and Cellular Therapy, St. Jude Children’s Research Hospital 5417 , Memphis, Tennessee, USA; 2 Department of Host-Microbe Interactions, St Jude Children's Research Hospital 5417 , Memphis, Tennessee, USA; 3 Cornell Veterinary Biobank, Cornell University College of Veterinary Medicine 43317 , Ithaca, New York, USA; 4 National Research Center, Giza, Egypt; 5 Immunology Center of Georgia (IMMCG), Department of Physiology, Medical College of Georgia (MCG), Augusta University 160343 , Augusta, Georgia, USA; University of North Carolina at Chapel Hill, Chapel Hill, North Carolina, USA

**Keywords:** tonsils, crypts, squamous epithelial cells, cytokeratins, keratin, reticular epithelial cells, tropism, cytokines, chemokines, influenza viruses, sialic acid receptors

## Abstract

The tonsils have been identified as a site of replication for Epstein–Barr virus, adenovirus, human papillomavirus, and other respiratory viruses. Human tonsil epithelial cells (HTECs) are a heterogeneous group of actively differentiating cells. Here, we investigated the cellular features and susceptibility of differentiated HTECs to specific influenza viruses, including expression of avian-type and mammalian-type sialic acid (SA) receptors, viral replication dynamics, and the associated cytokine secretion profiles. We found that differentiated HTECs possess more abundant α2,3-linked SA (preferentially bound by avian influenza viruses) than α2,6-linked SA (preferentially bound by mammalian strains). This dual receptor expression suggests a role in influenza virus adaptation and tropism within the tonsils by facilitating the binding and entry of multiple influenza virus strains. Our results indicated the susceptibility of differentiated HTECs to a wide range of influenza viruses from human, swine, and avian hosts. Virus production for most strains was detected as early as 1 day post-infection (dpi), and typically peaked by 3 dpi. However, pandemic H1N1 virus showed remarkably delayed replication kinetics that did not peak until at least 7 dpi. Notably, influenza virus infection impacted the expression of cytokeratins in HTEC cultures, which correlated with altered cytokine secretion patterns. These patterns varied within the strains but were most distinct in swine H3N2 infection. In conclusion, differentiated HTECs exhibited a strain-specific pattern of influenza virus replication and innate immune responses that included changes in cytokeratin and cytokine expression. These studies shed light on the complex interplay between influenza viruses and host cells in the tonsils.

**IMPORTANCE:**

To develop effective interventions against influenza, it is important to identify host factors affecting pathogenesis and immune responses. Tonsils are lymphoepithelial organs characterized by infiltration of B and T lymphocytes into the squamous epithelium of tonsillar crypts, beneath which germinal centers play key roles in antigen processing and the immune response. Influenza virus tropism in the human upper respiratory tract is a key determinant of host-range, pathogenesis, and transmission. Accordingly, experimental models using primary cells from the human respiratory tract are relevant for assessing virus tropism and replication competence. Our study addresses the dynamics of influenza virus replication in HTECs, including cellular tropism, infectivity, and cytokeratin and cytokine expression. The results of this study highlight the complex interplay between structural proteins and immune signaling pathways, all of which provide valuable insights into host-virus interactions.

## INTRODUCTION

Influenza viruses are an important health concern, causing seasonal epidemics and posing risks of pandemics (1–4). Identifying the host and virologic factors that contribute to influenza severity is critical to influenza control (5, 6). Wild aquatic birds are the natural hosts for influenza A viruses (IAVs) (7). Since avian IAVs rarely infect humans directly, it is widely believed that human IAVs emerged through pigs, which can be infected by both avian and human IAVs (8). For example, the 2009 H1N1 pandemic virus (pH1N1) was transmitted to humans from pigs infected with a mix of avian, swine, and human IAVs (9). As cases of IAV infections in other mammals increase, it is important to investigate the tropism and susceptibility of the oral cavity and lymphoepithelial tissues, such as tonsils, as potential sites for IAV adaptation.

The tonsils are lymphoid organs located on either side of the back of the throat (10) that contain an abundance of immune cells, including T and B cells. Tonsils also contain actively differentiating epithelia, which are organized into stratified squamous epithelium and reticular crypts (11–13) that are rich in microvilli and microfold cells (10, 14). Previous findings (15) indicate roles for soft palate in influenza virus adaptation and transmission. The soft palate extends downward past the anterior and posterior palatine tonsils (16) suggesting that the tonsils may play a key role in this process. The reticular crypts are branched invaginations lined by stratified squamous epithelia that extend throughout the full thickness of the tonsils (14). These crypts represent a specialized compartment that is critical for immunologic functions and plays an important role in facilitating contact between environmental factors (including viruses and other microorganisms) and lymphoid tissues (12, 17). At the tonsillar surface, the stratified squamous epithelium differs from a simple epithelium by controlling tissue integrity and mediating intracellular signaling pathways. The stratified squamous epithelium also facilitates immunologic functions against pathogens (including those causing respiratory and gastrointestinal infections) by antigen sampling and the movement of lymphocytes, cytokines, and chemotactic molecules from the tonsils to other lymphoid organs (11, 18). Thus, the complex structure of the tonsils enables them to enhance several local and systemic immunologic functions, thereby contributing to both innate and adaptive immune responses involving both cellular and humoral components (11, 19, 20).

Human tonsillar lymphoid cells have been reported to be initial sites of replication for several viral pathogens, including polyomavirus (21), enterovirus (22), and Epstein–Barr virus (23, 24). Porcine tonsils are primary infection sites for foot and mouth disease virus, with high levels of viral shedding into the environment due to extensive viral amplification at this site (25, 26).

Tonsillar epithelial cells are rich in cytokeratins (CKs) (27), especially CK5 and CK14, which are expressed on the epithelial surface, and CK19, CK8/18, and CK8, which are specific for crypt epithelial cells (12, 13). CKs are structural proteins that maintain cell integrity, prevent viral entry (28), and have roles in cell adhesion (29), signaling pathways, innate immune responses, and various cellular functions, including apoptosis (29–37). Some viruses (e.g., human papillomavirus and herpes simplex virus) target epithelial cells and modulate cytokeratin expression, potentially impacting tissue homeostasis and cancer progression (28, 36, 38). Additionally, CK8/18 serves as a useful marker for detecting microfold cells, which play a role in antigen uptake and presentation by the immune system (37, 39). Normal tonsillar crypt epithelium is rich in CK19, with its increasing expression reported to be a diagnostic marker for poor cancer prognosis (29). Despite the significant role of tonsillar epithelial cells in responding to other infections, the specific impact of these cells in responding to influenza virus infections is not well documented. Understanding the dynamic interaction between tonsillar epithelial cells and influenza viruses can inform control strategies.

To test our hypothesis that the tonsils play a role in influenza virus infection, we developed a differentiated *in vitro* model of human tonsil epithelial cells (HTECs). By using an air–liquid interface (ALI) culture, we were able to investigate the expression of sialic acids (SAs) on HTECs, the tropism and infectivity of different strains of influenza viruses in these cells, the impact of the infection on cytokeratin expression, and the resulting cytokine responses.

## RESULTS

### Morphological examination of differentiated HTEC cultures

**Fig 1:**
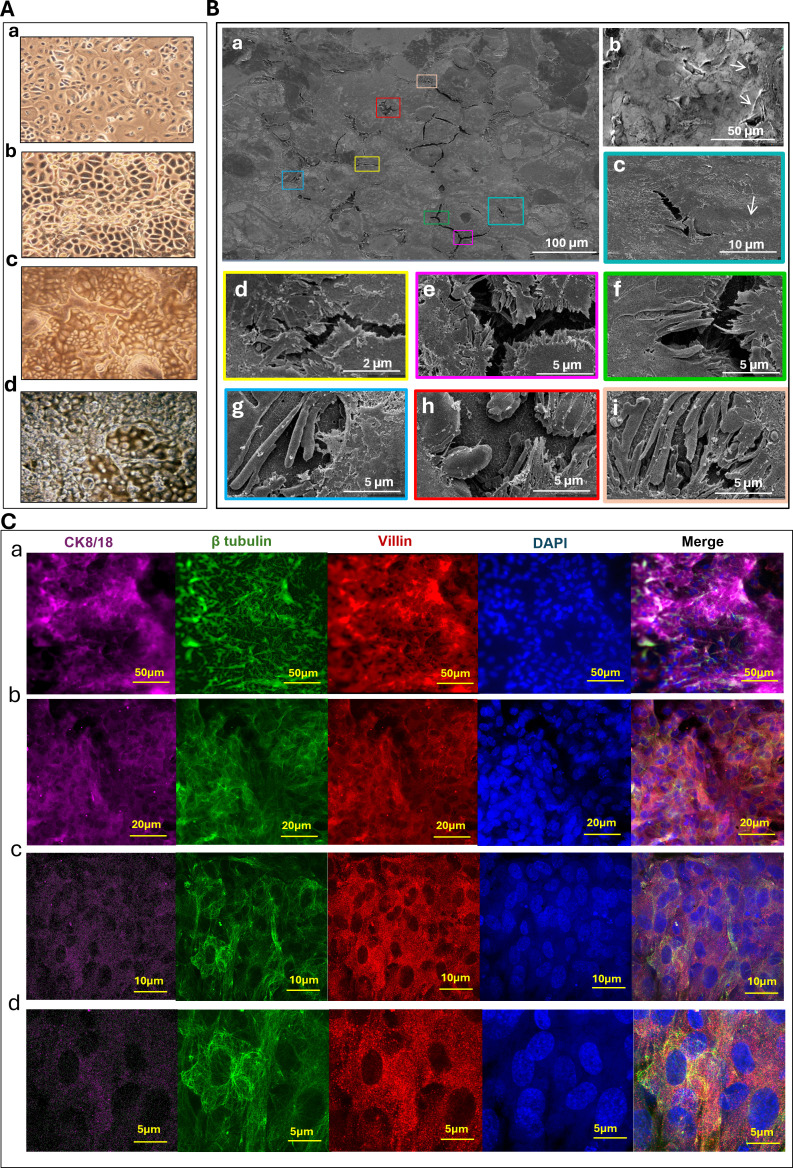
Characteristics of well-differentiated HTECs. (**A**) Phase-contrast microscopy images of primary HTEC cultures at different time points during the differentiation process (20× magnification): (A.a) day 0 at the ALI, (A.b) day 5 at the ALI, (A.c) day 10 at the ALI, and (A.d) day 30 at the ALI, revealing well-differentiated epithelial cells of different sizes, types, and cytoplasm-to-nucleus ratios. (**B**) SEM provided details of differentiated HTEC characteristics. (B.a) The apical surface of the HTECs. Note the heterogeneous cell sizes and shapes, representing squamous surface epithelial cells disrupted by reticulated crypts (detailed in insets). (B.b) Increased magnification showing structures resembling cilia (arrows). (B.**c–B.i**) Areas of higher magnification (color-matched to larger figure) highlighting tight junctions between cells (B.c; arrow) and crypts rich in microvilli (d–i; scale bar: 2, 5, or 10 µm, as indicated). Note the mesh-like arrangement of crypts. (**C**) Confocal immunofluorescence imaging of the HTEC culture apical surface. HTECs were fixed and stained at 15 days in culture with monoclonal antibodies against villin (Alexafluor594; red) as a marker for microvilli, and intracellular β-tubulin (Alexafluor488; green) associated with cytoskeletal microtubules and sparse cilia. The isotype background is shown in Fig. S1. Scale bars represent 50 µm in panels (C.a; 20× magnification), 20 µm in (C.b; 40×), 10 µm in (C.c; 63×), and 5 µm in (C.d; 68×). Additional fields are given in Fig. S1. Panel (C.d) displays a *Z*-stack (10 layers) orthogonal view, highlighting the surface localization of cilia (green) and microvilli (red). CK8/18 (Alexafluor647; magenta) is included as a cell marker. The complete *Z*-stack is provided as Video S1 and at a higher resolution in Video S2.

To explore the susceptibility of the tonsils to influenza virus infection and subsequent cytokine responses, we established a highly differentiated HTEC model. Phase-contrast microscopy images revealed that the HTECs in this system were heterogeneous, comprising cells of different sizes and shapes, representing squamous surface epithelial cells disrupted by reticulated crypts (Fig. 1A). Scanning electron microscopy (SEM) imaging (Fig. 1B) revealed that the apical surface of the differentiated HTECs was invaginated by crypts (Fig. 1B), had detectable cilia (Fig. 1Bb, arrows), and displayed abundant tight junction between cells (Fig. 1Bc, arrow). In fact, crypt-like features developed abundantly on the articulated curved surface membrane (Fig. 1Bd through f), as did microvilli (Fig. 1Bd through i). Overall, the HTEC model exhibits a highly differentiated microsystem of cells that mirrors the morphological features of *in vivo* tissue.

### Structural protein examination of differentiated HTEC cultures

**Fig 2:**
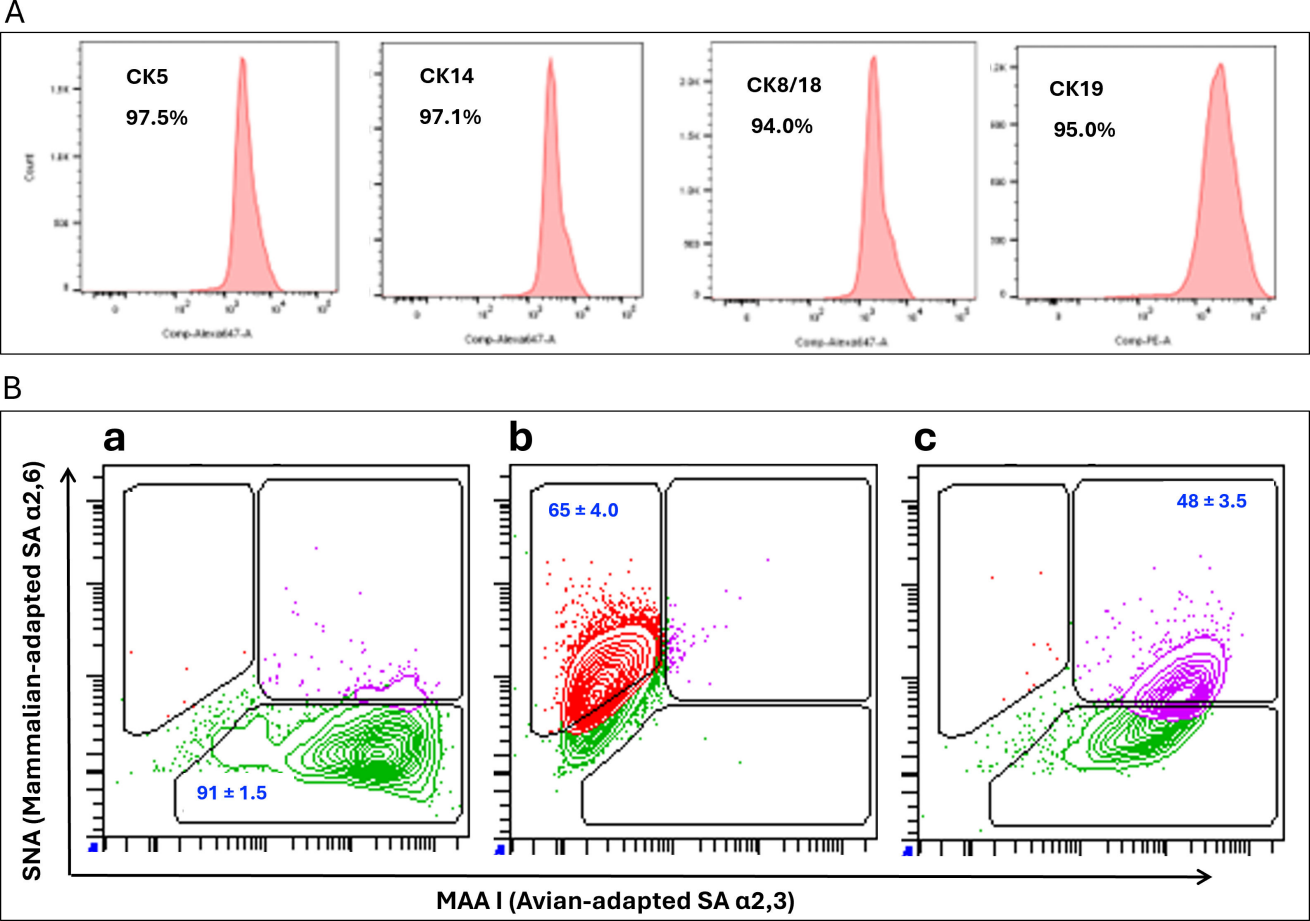
Distribution of CK and lectin staining in HTECs as determined by flow cytometry. (**A**) HTECs were fixed and permeabilized, then stained with monoclonal antibodies specific for CK5, CK14, CK8/18, and CK19 for flow cytometric analysis. Percentages give fraction positive for the indicated CK when gated against matched isotype controls. (**B**) HTECs were stained with MAA I-FITC lectin for avian-adapted α2,3-linked SA receptors or with SNA-BV786 lectin for mammalian-adapted α2,6-linked SA receptors. (B.a) MAA I lectin staining was nearly universal in more than five replicate experiments. (B.b) SNA lectin staining was more limited over the same experiments. (B.c) Dual staining showed that coincident expression of avian with mammalian-adapted SA in HTECs was robust. Gating is based on unstained and fluorescence-minus-one controls as shown in Fig. S2. Inset statistics give means ± SEM of the indicated populations from three replicate experiments.

To further characterize the HTEC model, we used immunofluorescence microscopy and flow cytometry to identify structural proteins that could be responsible for the structures seen in the scanning electron micrographs. Specifically, we probed for β tubulin (marking cilia), villin (marking microvilli), and several CKs. Immunofluorescence data showed that the HTECs expressed both villin and β tubulin (Fig. 1C; Fig. S1b and c). Furthermore, flow cytometry showed that HTECs uniformly and highly expressed CK5, CK14, CK8/18, and CK19 (Fig. 2A).

### Sialic acid receptor distribution in differentiated HTEC cultures

As influenza viruses utilize SA for adhering to cell surfaces (40), understanding SA distribution on HTECs could help define the susceptibility of these cells to different influenza virus strains. Sambucus nigra lectin (SNA), derived from elderberry bark, has a strong affinity for SA linked to the terminal galactose in α−2,6 linkages and a minimal affinity for α−2,3 linkages. In contrast, Maackia amurensis lectin (MAA 1/MAL I), extracted from Maackia amurensis seeds, has a high affinity for α−2,3 SA linkages (40, 41). To determine the SA distribution in well-differentiated HTECs, we used fluorescence-conjugated MAA I and SNA lectins to visualize α2,3-linked (avian-adapted influenza virus-preferred) and α2,6-linked (mammalian-adapted influenza virus-preferred) SA receptors, respectively, by flow cytometry (42) (Fig. 2B). This showed that the vast majority of HTECs stained for SA utilized by avian-adapted influenza viruses (91% ± 1.5%; Fig. 2Ba), while a smaller fraction was positive for mammalian-adapted SA (65% ± 4.0%; Fig. 2Bb). When combined, nearly half of cells (48% ± 3.5%) showed affinity for both lectins, suggesting that mammalian- and avian-adapted SA were simultaneously present on these cells (Fig. 2Bc). These data support a mechanism whereby HTECs could be infected by both mammalian- and avian-adapted influenza viruses.

### Influenza virus susceptibility and growth kinetics in differentiated HTEC cultures

**Fig 3:**
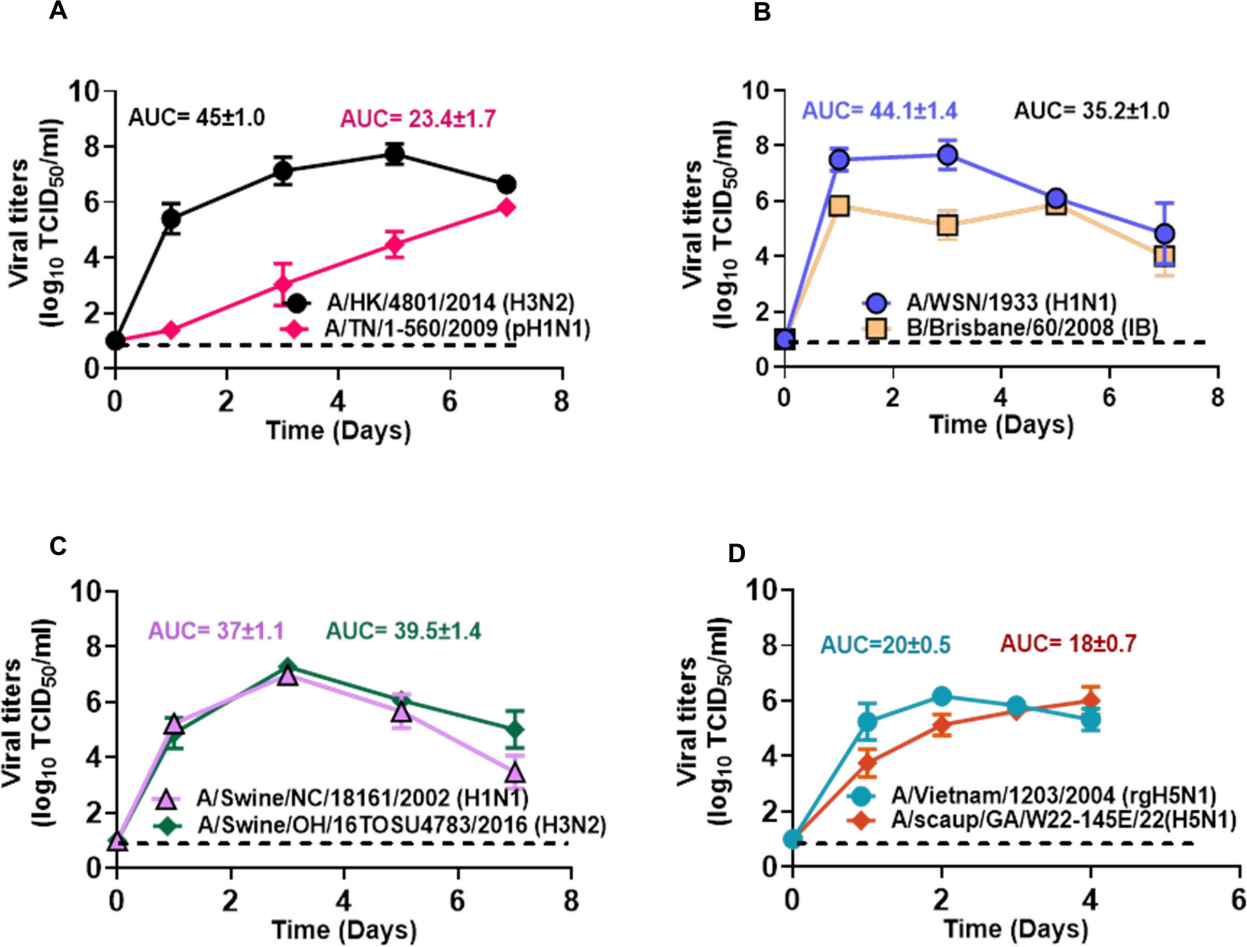
Influenza virus growth kinetics in HTECs. Well-differentiated HTECs were inoculated at a multiplicity of Infection (MOI) of 0.1 with the designated influenza viruses. (**A**) Human viruses A/TN/1–560/2009 (pH1N1) and A/HK4801/2014 (H3N2). (**B**) Human viruses A/WSN/1933 (H1N1) and B/Brisbane/60/2008 (IB). (**C**) Swine viruses A/Swine/NC/1816/2002 (H1N1) and A/Swine/OH/15TOSU4783/2016 (H3N2). (**D**) Strains A/Vietnam/1203/2004 (H5N1) and Avian A/scaup/GA/W22-145E/22 (H5N1). Viruses released from the cells were harvested with supernatants at the indicated time points and titrated to determine the TCID50 levels. The area under the curve (AUC) means ± SDs of three independent experiments, each using three HTEC cultures, are presented.

To determine whether well-differentiated HTECs can indeed support the replication of human, swine, and avian influenza viruses, we examined the growth kinetics of eight different strains (Fig. 3). All tested viruses replicated productively, albeit with different kinetics, in HTECs, reaching peak titers of approximately 6 × 10^6^ TCID50/mL. These data indicate that this model is supportive of and permissive for the growth of diverse influenza viral strains. The most delayed replication was observed for A/TN/1–560/2009 (pH1N1; Fig. 3A). The other seven strains tested, A/HK/4801/2014 (H3N2), B/Brisbane/60/2008 (IB), A/WSN/1933 (H1N1), A/swine/OH/16TOSU4783/2016 (H3N2), A/swine/NC/18161/2002 (H1N1), A/Vietnam/1203/2004 (rgH5N1), and A/scaup/GA/W22-145E/2022 (H5N1) replicated rapidly and attained measurable titers by 1-day post-infection (dpi). In fact, B/Brisbane/60/2008 (IB) and A/WSN/1933 (H1N1) achieved peak viral titers by 1 dpi (Fig. 3B). In contrast, A/HK/4801/2014 (H3N2) did not attain a peak titer until 5 dpi. Similarly, A/TN/1–560/2009 (pH1N1) virus replication was delayed even further, and while its titers continued to increase slowly, it did not produce a defined peak within the 7-day duration of this study (Fig. 3A). Both tested swine viruses replicated efficiently in differentiated HTECs and reached peak titers at 3 dpi (Fig. 3C). The HPAI A(H5N1) viruses also replicated efficiently in these cells (Fig. 3D) and caused robust cytopathic effect with destruction of the culture between 4 and 5 dpi. Overall, consistent with the presence of α2,3-linked and α2,6-linked SA receptors, HTECs support the replication of swine, avian, and human influenza viruses and may serve as important sites for viral replication within the respiratory tract.

### Scanning electron microscopy of infected differentiated HTEC cultures

We next used scanning electron microscopy to determine how A/TN/1–560/2009 (pH1N1) and A/Swine/OH/15TOSU4783/2016 (H3N2) infection affects the morphology of HTECs (Fig. S3 and S4, respectively). HTECs infected with A/TN/1–560/2009 (pH1N1) for 5 days, which had not yet reached peak supernatant titers (Fig. 3A), had intact cell membranes, although some cells had peeled layers, elongated cilia, and/or disrupted membranes (Fig. S3). Other notable observations were collapsed cells with exposed actin cytoskeleton and adjacent cells connected by filopodia. In HTECs infected with A/Swine/OH/15TOSU4783/2016 (H3N2), an abundance of collapsed cells was observed at 5 dpi, with many exhibiting disrupted/peeled membranes, condensed cell debris, profuse vacuoles, exposed actin cytoskeleton, and filopodia connections (Fig. S4). This increase in cellular damage is also consistent with the advanced stage of viral replication reported for this strain in Fig. 3C. These results show that influenza virus infection leads to morphological changes in HTECs, and these changes correlate with virus production in our model system.

### Cytokine and chemokine responses of infected differentiated HTEC cultures

**Fig 4:**
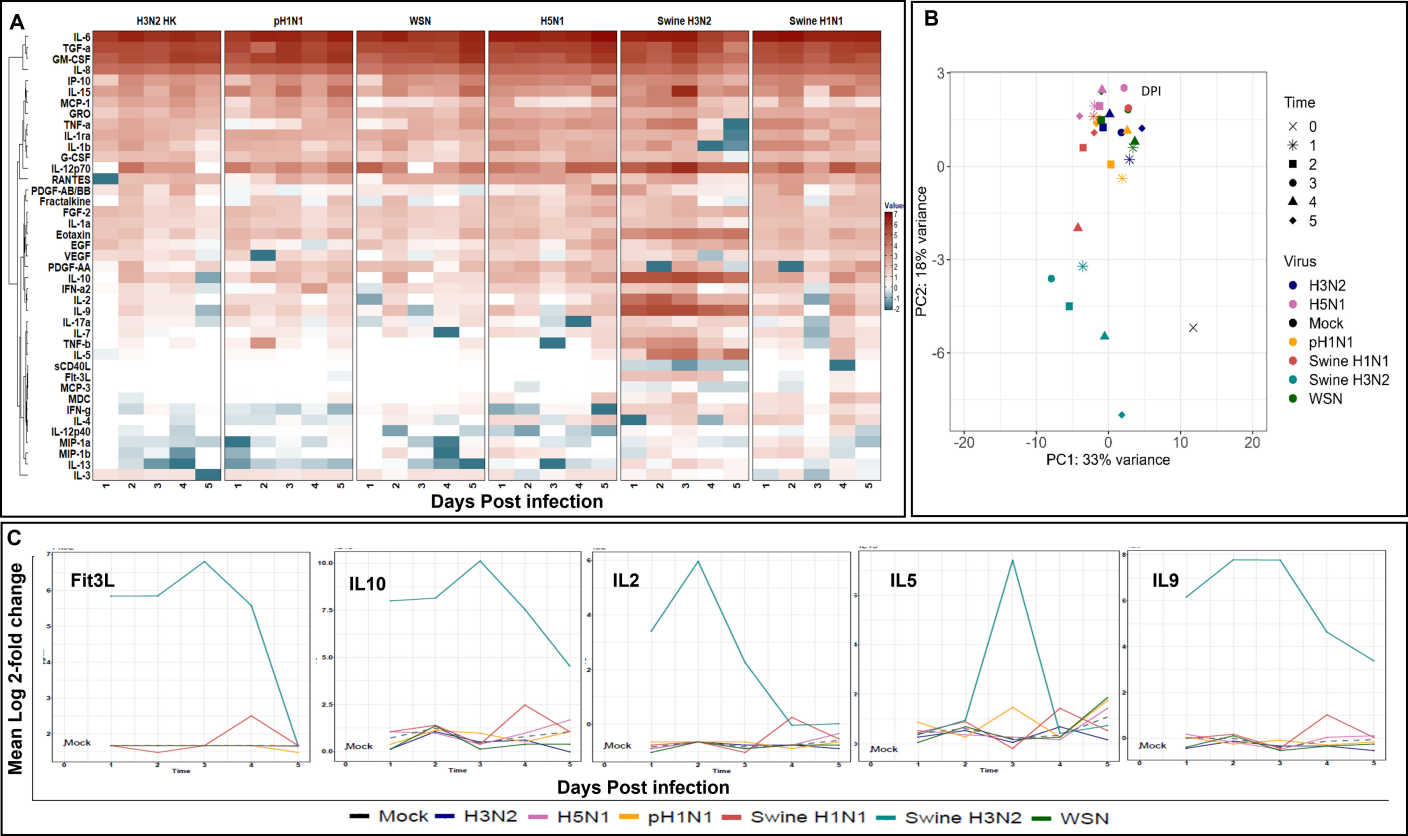
Longitudinal immune response profile of virus-infected HTECs. At the indicated times post viral infection, HTEC supernatants were collected and analyzed using a MILLIPLEX Human Cytokine/Chemokine/Growth Factor Panel. (**A**) Heat map showing the cytokine response in HTEC cultures to influenza virus infection over time. The colors shown represent the mean log_2_ fold changes in cytokine/chemokine concentration relative to mock-infected cells from two independent experiments that included three internal replicates, each. Analytes are grouped hierarchically based on similar expression patterns, as indicated by the clustering diagram at left. The complete linear regression on the log_2_ fold changes for each cytokine, with the virus and time as independent variables, including *P*-values, is given in Table S1. (**B**) Principal component analysis of log_10_-transformed cytokine immune responses in HTEC cultures by strain and time post-infection. Strains are denoted by color, and the time post-infection (0–5 days) is indicated by shape. Note the unique clustering of Swine H3N2 data points. (**C**) Detail of selected cytokines with distinctly elevated levels of expression in HTEC cultures infected with Swine H3N2 relative to the other viral strains tested.

In order to further explore the innate immune signaling of HTECs during influenza virus infection, we employed a MILLIPLEX Human Cytokine/Chemokine/Growth Factor Panel (HCYTA-60K Millipore) to test supernatants from separate HTEC cultures infected with each of six influenza viruses: A/HK/4801/2014 (H3N2), A/TN/1–560/2009 (pH1N1), A/swine/OH/16TOSU4783/2016 (H3N2), A/swine/NC/18161/2002 (H1N1), A/WSN/1933 (H1N1), and A/Vietnam/1203/2004 (H5N1). These results are summarized in Fig. 4A.

In brief, all influenza virus strains clearly exhibited rapid induction of numerous analytes within the first day of infection. These data have been presented as a heatmap (Fig. 4A) to illustrate the correlations between the samples and the cytokines. Throughout the 5-day course of influenza virus infection, several specific cytokines and chemokines were secreted by HTECs *in vitro*, regardless of the infecting viral strain. For example, both the tested neutrophil chemo-attractants GROa (also known as CXCL1) and IL-8 (also known as CXCL8) were upregulated as early as 1 dpi and stayed elevated through 5 dpi (Fig. 4A). Likewise, the proinflammatory cytokines IL-1a and IL-1ra were upregulated to a similar extent as GRO. In all, the most acute changes in expression uniform to all influenza virus strains were found in the increased secretion of TGFα, G-CSF, GM-CSF, IL-6, IL-8, IP-10, and IL-15.

In contrast, the secretion of some signaling molecules was strain-specific. For example, IFN-γ was at mock infection levels or below for the four human influenza viruses tested but elevated for the swine strains (Fig. 4A). This finding is interesting since IFN-γ is known to contribute to a Th1-type immune response in conjunction with IL-12p70 signaling (43). IL-12p70 was found in the supernatants of all influenza virus infections tested. At the same time, IL-4 was also present at lower levels in the swine influenza virus supernatants, suggestive of a Th2-type immune response (44). There were also several signaling molecules that dropped below mock-infected levels, including IL-12p40 and MCP-3.

To better quantify the differences observed from the heatmap analysis, we performed principal component analysis on all samples for all cytokines. Plots of the first two components showed a clear difference between swine H3N2 and all other viruses across each time point (Fig. 4B). We further tested these effects by performing linear regression on the log_2_ fold changes for each cytokine, with the virus and time as independent variables. These are assembled in Table S1, including *P*-values for each as a test of statistical significance for the clustering, relative to mock-infected controls. One cluster was unique to swine H3N2 infection and included Flt3L, IL-2, IL-10, IL-5, and IL-9. Each of these signaling molecules was induced at high levels in response to swine H3N2 infection in HTECs across the measured time points yet remained at mock-infected levels for all other viruses (Fig. 4C). Again, this cluster was separate from the group of cytokines noted above (G-CSF, TGFα, GM-CSF, IP-10, and IL-15, IL-8, and IL-6) that showed elevated responses similar for all viruses when compared with those in mock-infected cells (Fig. 4A). The differential cytokine responses elicited by various influenza virus strains in HTECs underscore the complexity of the host-pathogen interaction.

### Cytokeratins expression of infected differentiated HTEC cultures

The ability of HTECs to be infected by a wide array of influenza viruses implicates the tonsils as a potentially important site of influenza virus replication and early host responses. CKs are vital for cell signaling in early innate responses, such as those we documented with infected HTEC cultures in Fig. 4 (45–47). To assess the relationship between CK expression in HTECs and influenza virus infection, HTECs were infected with the human strains A/HK/4801/2014 (H3N2) or A/TN/1–560/2009 (pH1N1), or with the swine strains A/Swine/NC/1816/2002 (H1N1) or A/Swine/OH/15TOSU4783/2016 (H3N2), and analyzed for CK5, 14, 8/18, or 19 expression as well as influenza virus infection (by measuring FluNP-positive cells) via flow cytometry.

**Fig 5:**
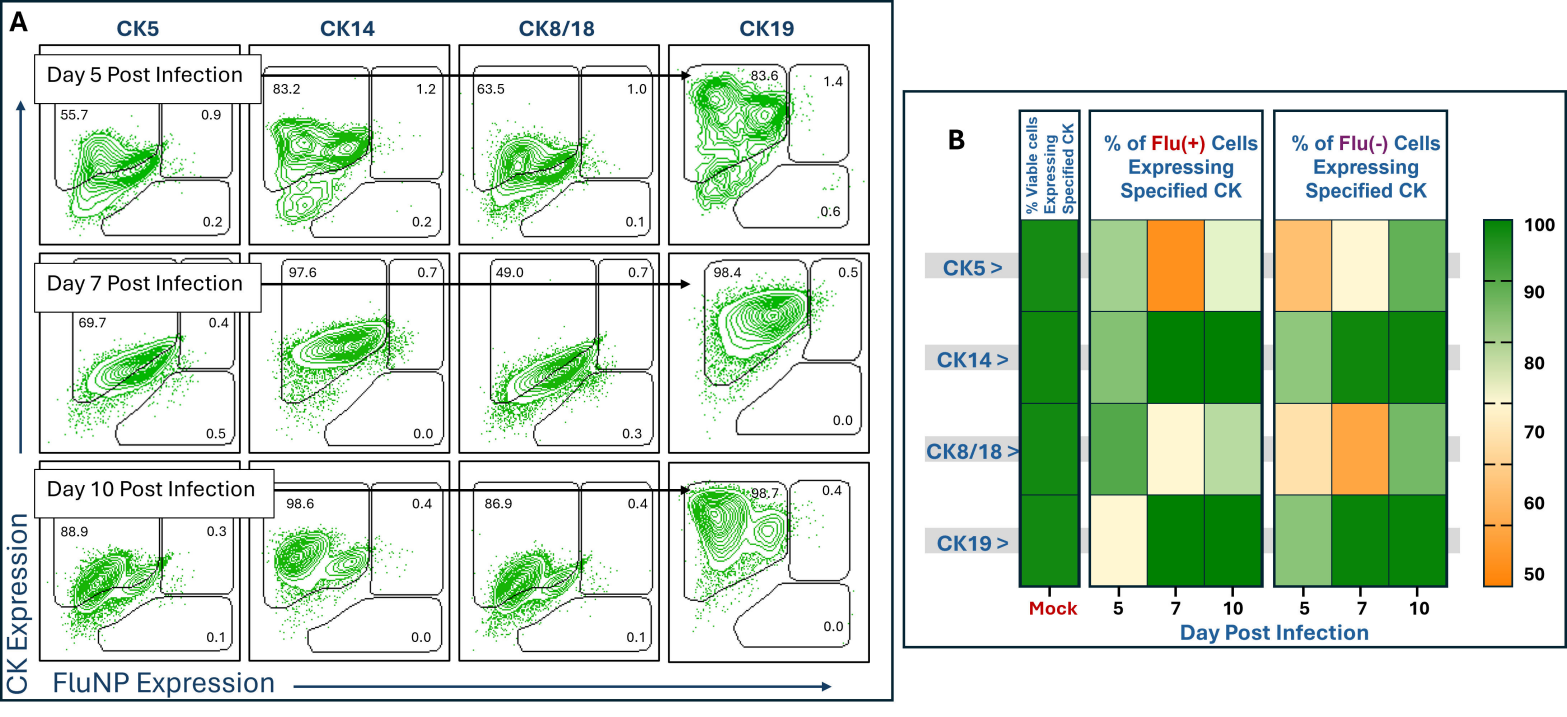
CK expression in pH1N1 influenza-infected HTECs. HTECs were infected with A/TN/1–560/2009 (pH1N1) at an MOI of 0.1. Infected HTECs were collected at 5, 7, and 10 dpi, then fixed, permeabilized, and stained with anti-influenza nucleoprotein monoclonal antibody (FluNP), as well as with monoclonal antibodies against either CK 5, CK14, CK8/18, or CK19, then analyzed by flow cytometry. (**A**) Representative flow plots showing the trend of decreased CK expression at early times (day 5) post-infection, and the subsequent return to nearly uniform CK expression as time of infection progressed. CK5 gives the most dramatic example of CK expression dynamics, while CK14 and CK19 show nearly complete return to mock-infection levels of CK expression. Gates were based on unstained, isotype, and fluorescence-minus-one controls (Fig. S5). (**B**) Heatmap summary of the data shown in (**A**). Percentages are the fraction of viable HTECs from either the FluNP(+) or FluNP(−) cohorts within the culture that expressed each CK at the time point assayed. While both cohorts experienced decreased CK expression, note the pronounced decrease in CK expression in uninfected cells, especially for CK5 and CK8/18 (*n* = 6 inserts/each virus/each time point).

As shown in Fig. 5, HTEC cultures infected with A/TN/1–560/2009 (pH1N1) showed a lower number of FluNP(+) cells, consistent with its slower replication dynamics relative to the other viruses tested (Fig. 3). Interestingly, despite this low level of infection, the frequency of CK-expressing cells had decreased noticeably at 5 dpi for all four CK assayed. This was distinct in both FluNP(+) and FluNP(−) populations, relative to mock-infected cultures; however, it was most pronounced among cells not infected with virus (Fig. 5B). This fluctuation affected CK5 and CK8/18 more prominently than CK14 and CK19, which had returned to nearly mock-infected frequencies by 10 dpi. The percentage of CK(+) cells had increased for CK5 and CK8/18 by this time point as well but not to the same extent (Fig. 5B).

**Fig 6:**
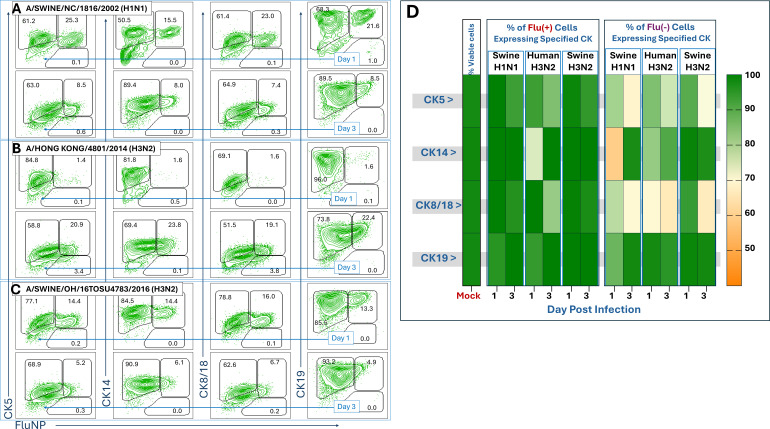
CK expression in HTECs infected with three different strains of influenza. (**A–C**) HTECs were infected with A/Swine/NC/1816/2002 (sH1N1), A/HONG KONG/ 4801/2014 (H3N2), or A/Swine/OH/ 15TOSU4783/2016 (sH3N2), at an MOI of 0.1. Infected HTECs were collected at 1 or 3 dpi and assessed by flow cytometry, as described for Fig. 5. Specific CK expression is given vertically, and FluNP expression is shown horizontally. Fluorescence-minus-one gating plots are given in Fig. S6. (**D**) Heatmap summary of data from A–C showing the fraction of total viable HTECs from either the FluNP(+) or FluNP(−) cohorts within the culture that expressed each CK at the time point assayed. Notably, CK expression decreased in both cohorts but was more pronounced in uninfected HTECs (*n* = 6 inserts/each virus/each time point).

Similarly, the three influenza strains specified in Fig. 6 each showed remarkable decreases in the frequency of CK(+) cells, again most notably among the FluNP(−) cohort. This phenomenon was most pronounced with cultures infected by the Swine H1N1 strain but repeated reliably with each of the other two strains tested. Again, CK5 and CK8/18 were the most affected, while CK19 saw only a minimal disruption (Fig. 6D). In contrast, cells expressing FluNP, and presumably actively infected with influenza virus, showed much smaller disruptions in CK expression across all three of these viruses and at both times assessed (Fig. 6D). This modulation of CK(+) cells that are negative for influenza NP compared to infected cells in the same culture suggests a possible role for paracrine signal inhibition from influenza virus-infected cells affecting expression of these CKs (Fig. 5B and 6D).

### Cytokeratin-cytokine correlations in infected differentiated HTEC cultures

**Fig 7:**
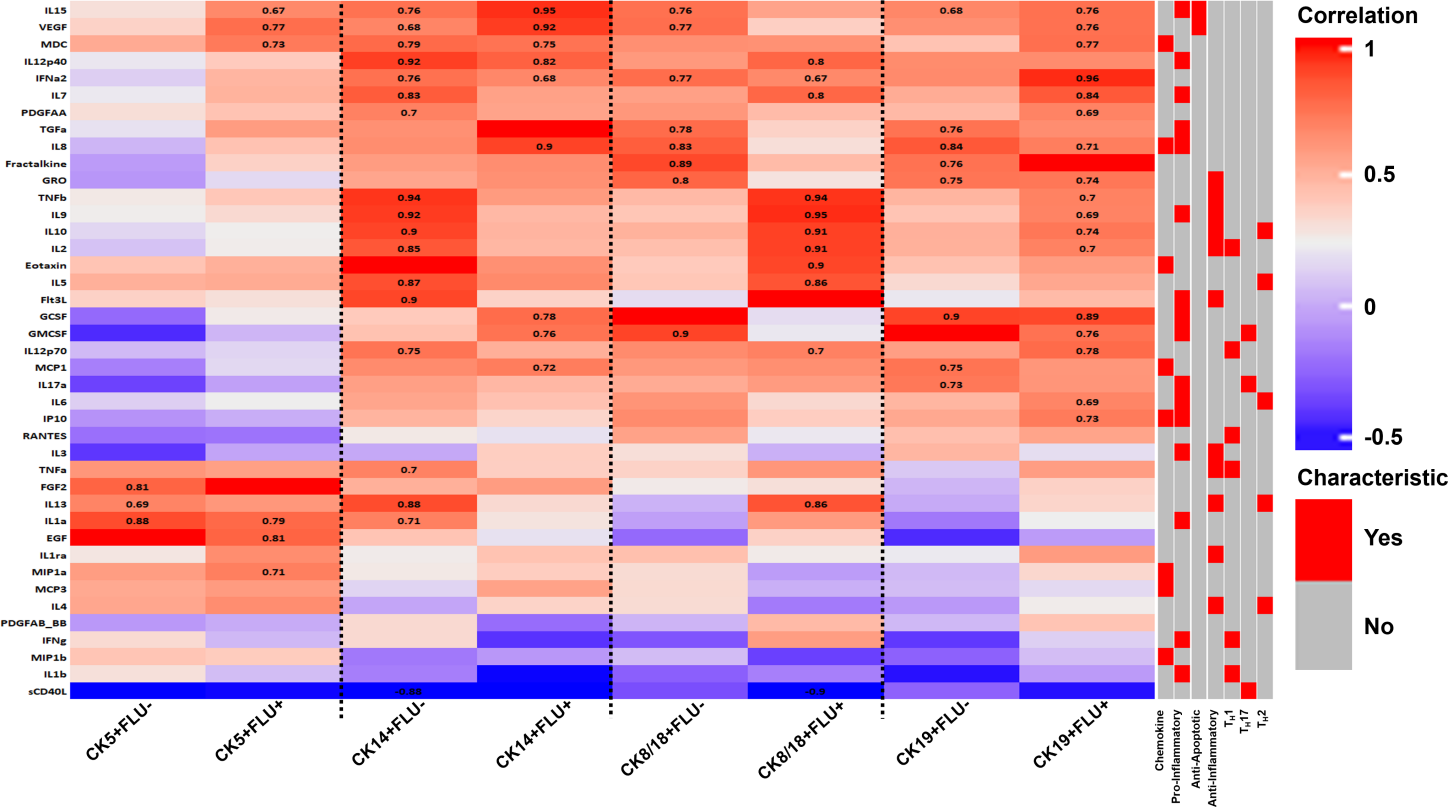
Correlations between cytokine production and influenza vs CK populations after influenza infection of HTECs. Data from Fig. 4 to 6 were combined to determine correlations between cytokine expressions, influenza infection (FLU+ or FLU−), and specific CK expression in HTEC cultures. The data include all influenza strains and time points after infection. Colors represent the correlation coefficient as determined by a Spearman correlation, and numbers mark those with statistical significance (*P* < 0.1). Cytokines are grouped by similar overall correlation patterns. Gray and red bars at the right highlight different general immunological characteristics.

In order to more formally test the possibility that paracrine signaling during influenza virus infection leads to modulation of CK expression in HTECs, we performed a correlation analysis between the cytokine data reported in Fig. 4 and the CK expression data given in Fig. 5 and 6. We combined data from strains and time points in this exercise to create an overview of interactions that could be detected between cytokine and CK expression patterns according to influenza-infected (FLU+) or non-infected (FLU−) populations expressing each CK. These data were then analyzed via a Spearman correlation (48) to visualize any relationships (Fig. 7).

Overall, the vast majority of the statistically significant correlations (*P* < 0.1) were positive, with only sCD40L levels showing a negative correlation for CK14+FLU− and CK8/18+FLU+ populations. When comparing major trends between cytokeratins, CK8/18 and CK19 were the most similar, including the production of the anti-inflammatory cytokines IL-2, IL-9, IL-10, and TNF-β among influenza-infected (CK+FLU+ ) populations. Interestingly, CK14 expression frequently showed an inverted correlative cytokine expression pattern relative to CK8/18 and CK19, with the same anti-inflammatory cytokines noted above more prominently produced by CK14-expressing cells not infected by virus (CK+FLU−). Several cytokines of different characteristics (chemokines, pro-inflammatory, anti-inflammatory, T_H_1, and T_H_2) showed a similar inverse relationship between these three CK-expression characteristics, including TNF-α, IL-13, IL-1a, Flt3, IL-12p70, IL-7, and IL-5. Conversely, IL-15, VEGF, MDC, IL-8, G-CSF, and GM-CSF shared a positive correlation among both CK14 and CK19 but not with CK8/18 expression within virally infected cells. Considering the shared CK modulation between CK14 and CK19 in response to influenza virus infection (Fig. 5B and 6D), it is tempting to consider that one or more of the shared cytokine correlations noted here might provide a mechanistic clue to this viral response.

Cytokine correlations with CK5 expression differed the most compared to the other cytokeratins. Most notably, none of the anti-inflammatory cytokines noted above in relation to the other three CK showed significant correlations with CK5, in either the FLU+ or FLU− cohorts. CK5 expression did show similarities in correlation among the chemokine and anti-apoptotic cytokines IL-5, MDC, and VEGF for FLU+ populations as in CK14 and CK19 but not with CK8/18. CK5 also shared similar correlations with CK14 for IL-1a and IL-13 in FLU− populations; however, beyond these highlights, the expression of CK5 did not correlate with many of the cytokine expressions exhibited with other cytokeratins, regardless of influenza positivity of the cells. In fact, CK5+FLU+ populations were the only ones tested to correlate with EGF and MIP1a production.

Further analysis of cytokine correlations with CK expression between FLU+ and FLU− populations may indicate which, if any, cytokines or inflammatory pathways could be responsible for the modulation of CK expression we detected. In general, our observations imply that, except among CK14-expressing subsets, more cytokines positively correlated with flu-infected (FLU+) populations than they did with the non-infected subsets. These observations support the notion that infection led to cytokine expression, which in turn triggered the reduction of CK expression in uninfected cohorts within the culture, via paracrine signaling pathways.

## DISCUSSION

Influenza viruses are transmitted by respiratory routes in humans and by the fecal–oral route in many avian species. The tonsils of humans are located at the entrance to both the respiratory and digestive tracts, whereas avian tonsils are located only in the digestive tract. This difference has suggested the role of tonsils in influenza virus infection (10, 49, 50). Previous studies have reported that the soft palate is an important site of adaptation for mammalian transmissible influenza viruses. Therefore, exploring the oropharyngeal region is implicated in better understanding of the characteristics that make this a key site for virus evolution and selection of airborne-transmissible influenza viruses (15). Our study has contributed to this endeavor by highlighting several characteristics of human tonsil epithelial cells that influence influenza virus infection.

To do this, we optimized an *in vitro* model system where primary HTECs maintained in an air–liquid interface culture system could differentiate to create secondary features reminiscent of *in vivo* tonsillar tissue, including structures resembling crypts (Fig. 1B). Cilia and villi were also detected by electron microscopy (Fig. 1B), and their presence was substantiated by immunofluorescence staining for β-tubulin and villin, respectively (Fig. 1C; Fig. S1b and C). Subsequent flow cytometry showed that HTECs in this culture system also expressed high levels of CKs, including CK8/18 and CK19 (Fig. 2A), shown in other systems to be predominantly expressed in crypt epithelial cells (12, 13). CK5 and CK14, indicative of surface epithelial cells (12, 24, 51), were also detected at high levels (Fig. 2A).

Sialic acid receptors are crucial for influenza virus cellular attachment and entry (52). Avian influenza viruses preferentially bind α2,3-linked SA, whereas mammalian influenza viruses preferentially bind α2,6-linked SA (40, 42, 53, 54). Acquiring mutations that foster switching of binding specificity between α2,3-linked and α2,6-linked SA receptor preferences is key for avian influenza viruses to transmit efficiently in humans (52, 55, 56). Both α2,6-linked and α2,3-linked SA receptors were found in our HTEC model (Fig. 2B). Consistent with this, our HTECs were permissive for the growth of different influenza strains from mammalian and avian hosts, albeit with heterogeneity in virus infectivity and growth properties (Fig. 3).

The observation that HTECs possess a higher proportion of α2,3-linked SA receptors than α2,6-linked SA receptors is intriguing. This finding aligns with the results of previous research indicating that avian influenza viruses can infect humans, even when the viral surface proteins possess a receptor-binding preference for α2,3-linked SA receptors (40, 55). Matrosovich et al. (57) reported that differentiated cultures of human tracheobronchial epithelium predominantly express α2,6-linked sialic acids on nonciliated cells, while ciliated cells express α2,3-linked sialic acid receptors at a density sufficient for avian virus entry and replication (57). This contrasts with our results in HTECs, which contain few ciliated cells, but is consistent with others (58) reporting that normal human primary bronchial epithelial cells (NHBE) express both forms of sialic acid, with α2,6-linked sialic acids being more abundant than α2,3-linked sialic acids (58).

Although we did not determine the detailed SA-binding preferences of the viral strains used in this study, it is tempting to postulate that subtleties in receptor preference coupled with viral polymerase functions may mediate the differences in virus growth kinetics and the subsequent cytokeratin and cytokine responses observed here. A more robust analysis of virus panels with known receptor specificities is required to further explore this possibility. Both avian viruses used in this study are highly pathogenic avian influenza (HPAI) strains and, therefore, raise pandemic concerns when considering adaptation to an α2,6 receptor preference (15, 40, 42, 54).

HPAI A(H5N1) viruses of clade 2.3.4.4b have been recently detected in milk and nasal swabs from infected cattle (since late March 2024), and cats that consumed milk from infected cattle have reportedly died, highlighting this strain’s pathogenicity (59, 60). Mice orally inoculated with the virus showed signs of illness and high virus titers in the respiratory organs, indicating potential infection via the pharynx, as well as moderate virus titers in several other organs (61). The respiratory and mammary glands of HPAI H5N1-infected dairy cattle are rich in SA, particularly the avian influenza virus-specific SA α2,3 (62, 63); however, little is known about the distribution of SA receptors in lymphoepithelium that are closely connected to mammary glands.

The data we report here show that HPAI A(H5N1) viruses of clade 2.3.4.4b replicated well in HTECs (Fig. 3D), which richly express both avian- and mammalian/human-like SA (Fig. 2B). Moreover, the coexistence of both avian and mammalian influenza virus receptors on a single-cell increases the risk of reassortment between viral strains (62, 63) as well as acquiring mammalian adaptation markers. Influenza viruses are transmitted by respiratory routes in humans and by the fecal–oral route in many avian species where they have the tonsils in the cecum (64–66). Cow tonsils are considered a specified risk material and must be removed from cattle of all ages in accordance with U.S. Department of Agriculture Food Safety and Inspection Service (FSIS) regulations due to potential infection with Bovine Spongiform Encephalopathy (67). Accordingly, it is important to highlight tonsils and lymphoepithelium as a potential shared tissue for viral adaptation, which may not necessarily be specific to any one animal group, such as swine, as has been previously proposed as mixing vessels for influenza virus pandemics (7, 8). To the best of our knowledge, our work reported here is the first to show this. The adaptation of HPAI H5N1 from birds to cows and subsequently to humans highlights the significance of cross-species transmission (68). The emergence of new variants occurs due to acquired mutations that enable H5N1 to infect mammals and enhance its transmissibility among humans (60). Therefore, it may be advisable to include tonsillar swabs in influenza virus surveillance efforts.

The replication of influenza viruses in other differentiated primary airway cells has been previously reported. pH1N1 virus can efficiently replicate in well-differentiated human bronchial epithelial (NHBE) cells as early as 30 hours post-infection (69, 70). Human influenza H3N2 viruses have also been shown to replicate efficiently in NHBE cells with kinetics slightly slower than that of pH1N1 viruses in one study (71), with this reversed in another (72), likely reflecting virus- and donor-specific effects. Similar to the latter study, we also observed a delayed replication of the pH1N1 virus in HTECs, which is a trend not observed in differentiated epithelial cultures derived from adenoid tissue (72). Other studies (73) reported that human H3N2 and avian H5N1 viruses can infect epithelial tissues of the tonsil, despite an apparent lack of avian receptors (40). This observation aligns with our data that both human H3N2 and avian H5N1 replicate efficiently in HTECs. However, it diverges from our findings, which indicate that both α2,3- and α2,6-linked sialic acids are present in HTECs, allowing for the replication of a broader range of influenza strains. In addition, Swine influenza viruses can also replicate in human bronchial epithelial cells (HBECs) to levels comparable to H1N1pdm09 (70). Furthermore, Calu-3 cells support robust replication of the HPAI-H5N1 and seasonal H3N2 viruses (74), which is consistent with our findings in HTECs.

Innate immune responses are rapidly activated following influenza virus infection (75). We also detected rapid induction of cytokine expression in virally infected HTEC cultures (Fig. 4A), suggesting that cytokine secretion from HTECs in response to viral challenge could impact early immune responses. Specifically, the abundant secretion of IL-6, TNF-α, and IL-8 would contribute to a robust pro-inflammatory environment, while a rapid innate immune response would be promoted by G-CSF- and IL-8-mediated recruitment of neutrophils and other granulocytes. Meanwhile, elevated IL-15 levels would encourage NK cell infiltration (76–78). The significant increase of certain cytokines in the tested swine H3N2, which is a human-like H3 IAV-S (clade 3.2010.1), could indicate a unique signaling pathway for this virus compelling more investigation. Certainly, the cytokine expression dynamics we observed would contribute to multiple cell signaling pathways. It is reasonable to expect that these, or other similar paracrine signals, could also direct the CK modulation we observed, although illuminating this, as well as any role it might play in an immune response, will require further studies.

Comparative studies indicate that HBECs possess a unique receptor distribution and immune response, impacting their capacity to support viral replication (58, 79–84). Gerlach et al. (69) reported early release of pro-inflammatory cytokines, including IFN-α, CCL2 (MCP-1), TNF-α, and CCL5 (RANTES), and changes in cytokine/chemokine levels of IL-6, IL-8, and GRO from well-differentiated NHBE cells when infected by pandemic viruses (69). This aligns with our findings in HTECs infected with H5N1, swine H3N2, and WSN, where we also observed early release of these cytokines, except with HTECs infected with pH1N1, which exhibited a delay release of both TNF-α and IFN-α, and in those infected with WSN, which experienced delayed MCP-1 production.

In other studies, H5N1 virus has been reported to induce IP-10, IFN-β, RANTES, and IL-6 mRNA production in human primary alveolar type II epithelial and HBEC cells (58, 81, 82). Likewise, fully differentiated NHBE cells infected with avian influenza viruses induce robust IP-10, IL-6, and RANTES responses early during infection compared to human H3N2 infection, as well as inhibition of the MCP-1 chemokine relative to mock-infected NHBE cells (58). In our HTEC model system, we also saw robust expression IP-10, RANTES, and MCP-1; however, MCP-1 secretion was released over time in WSN and swine H3N2 and declined rapidly after 24 h in H5N1-infected cells. Also, MCP-1 release fluctuated somewhat during both pH1N1 and swine H1N1 infections. RANTES also presented and expression dynamic in several strains, increasing with the time of infection with the human H3N2, pH1N1, and WSN strains but decreasing with time following swine H3N2 infection. Interestingly, we also found that several strains of influenza viruses induced significant anti-inflammatory cytokines, such as IL-10 and TGF-α, in well-differentiated HTECs (Fig. 4).

Our findings suggest that HTECs may exhibit cytokine response patterns reflective of the tissue’s specific role in immune regulation within the mucosal immune system. Importantly, tonsillar epithelial cells are part of Waldeyer’s ring, a specialized mucosal immune environment that is not commonly examined in influenza virus research. As a part of this structure, tonsils possess a distinct immune cell composition and cytokine milieu, which may prime these cells for specific responses to pathogens (11, 85). This aligns with our observation of heightened innate immune responses to swine H3N2 strains, suggesting that the tonsils provide early detection and response mechanisms against a wide variety of pathogens, including zoonotic viruses.

CKs play critical roles in cellular integrity and signaling (86). CK5 and CK14, expressed in basal epithelial cells, foster cell division and movement and contribute to epithelial repair following viral damage (87). CK8/18 and CK19, typically expressed in more differentiated cells, maintain the shape and structural integrity of the cell and protect cells under stress conditions (47, 88, 89), which may impact viral entry and replication. Furthermore, CK-expressing epithelial cells form a protective barrier against pathogens, including viruses (28, 30, 32, 33, 38). One striking observation from our study was the overall decrease in CK5 and CK8/18 positive cells in virally infected cultures relative to mock (Fig. 5 and 6). Indeed, CKs have been reported to be important for human papillomavirus (HPV) infection. The HPV E6 and E7 proteins modulate CK expression in infected cells, affecting cell proliferation and transformation and promoting viral persistence and pathogenesis (27, 28, 90). Others have suggested that infection-induced rearrangement of CKs, some induced by downstream impacts of cytoplasmic RNA recognition, may be involved in apoptotic cell death during virus infection (75, 91).

CKs are also recognized for their involvement in cell signaling pathways that govern immune responses during viral infection, including triggering inflammatory cytokine release (89, 92, 93) and inflammatory responses that can affect disease progression, viral latency, and assembly (91, 94). While the specific interactions between influenza viruses and CKs have not yet been well documented, it is reasonable to postulate that CKs rearrangement and/or phosphorylation may impact influenza viral entry and the assembly of viral replication complexes. Our finding of the changes in cytokeratin expression levels during influenza virus infections could also be indicative of cellular stress or disrupted cell-cell adhesion and integrity, which are common in microbial infections (28, 32). Here, the changes in CK expression, including in uninfected HTECs, might be in response to influenza virus disrupting normal cell function and differentiation processes.

Influenza viruses cause significant cellular changes leading eventually to apoptosis. They also activate multiple signaling pathways, including those related to interferon-stimulated genes, which play a critical role in cytokine production (75, 82, 92, 93). CK involvement has also been reported in cell signaling pathways during viral infection which triggers inflammatory cytokine release (88, 91, 95). Likewise, the CK modulation we report here may contribute to the release of pro-inflammatory cytokines, such as the IL-6 and TNF-α production we noticed in all strains tested (Fig. 4). We addressed this possibility by conducting a correlation analysis of cytokine data from influenza virus strains alongside corresponding CK expression data and probed for potential relationships between specific CKs and cytokines in both influenza-infected and uninfected cells.

One finding from this analysis was similarities in cytokine and CK expression correlation between CK8/18 and CK19 expressing cells, regardless of viral infection status (Fig. 7). It has been reported that the CK8/18 and CK19 work together to maintain the structural integrity and mechanical properties of epithelial cells, enhancing their resilience under stress (96). These connections may relate to the similarities we noted. Beyond this, it is noteworthy that positive expression correlations between CK and cytokines were most pronounced among influenza-infected cells in these studies, except with regard to CK14 (Fig. 7); however, CK expression was most dramatically affected in cells that were not yet virally infected (Fig. 5 and 6). At its surface, this relationship suggests a pathway whereby influenza-infected cells are secreting cytokines (and other molecules) that stimulate healthy neighboring cells to modulate their cytokeratins.

The advantage of this function, if any, to the immune response will need further study to understand; however, one clue may come from work focused on the influenza NS1 protein. This molecule is known to antagonize host innate immunity and interferon pathways by interacting with certain cellular proteins (97). Specifically, the small heat shock protein Hsp27 (HSP27) significantly impacts the assembly dynamics and structure of CK8/CK18 networks (89), and in separate studies, HSP27 has been shown to play a role in the innate immunity of influenza-infected cells by enhancing the inhibitory effect of NS1 on IFN-β expression (98). Alternatively, the breakdown of cytokeratin networks during apoptosis can influence the release of pro-inflammatory cytokines, thereby affecting the immune response to infection. This convergence of evidence suggests that cytokeratins not only serve structural roles but also actively participate in signaling pathways that can enhance or inhibit viral replication. At least, the changes in CK expression in healthy un-infected cells coexisting with influenza virus-infected cells we observed suggest the presence of a paracrine signal inhibition mechanism affecting CK expression. A further role for this in the immune response may be implied by previous work showing that immune cells can recognize altered CK patterns, and this recognition process influences their recruitment and activation cytokines (46). However, these interactions are multifaceted, and further research is needed and warranted to fully elucidate these aspects of influenza virus infection.

In conclusion, the HTEC culture system that we have generated provides a valuable *in vitro* model for studying the cellular tropism and infectivity of influenza viruses in humans. The ability of HTECs to support the replication of human, avian, and swine influenza viruses suggests that the tonsils may be important sites for viral replication, dissemination, and genetic reassortment. Moreover, investigating cytokeratin-related pathways could yield insights toward enhancing host defenses and limiting viral spread. Further research toward understanding the precise mechanisms underlying these interactions and their implications for influenza transmission, host immunity, and the development of targeted interventions is necessary.

## MATERIALS AND METHODS

### Air–liquid interface culture system

Primary HTECs were purchased from ScienCell Research Laboratories (Carlsbad, CA). According to the information provided by the company, the cells were collected from 22 children during tonsillectomies and pooled for the isolation of the primary cell line. The primary cells were certified as free from viruses or bacterial infection. HTECs were seeded on Transwell-COL collagen-coated polycarbonate membrane inserts (24-well, 0.4 µm pore size; Corning Costar) at a density of 2.5 × 10^5^ cells/well. Cells were then cultured in two different differentiation media: TEpiCM, consisting of 500 mL of basal medium, 5 mL of tonsil epithelial cell growth supplement with 5 mL of penicillin/streptomycin; and TEpiCM-ALI modified medium for differentiation (TEpiCGS, Cat. No. 2572). Once cells were confluent, cultures were continued at the ALI by removing the growth medium from the apical surface and replacing the basal medium every 2 days with Modified TEPiCM-ALI supplemented with hydrocortisone and heparin (STEMCELL technologies) according to the manufacturer’s instructions, as previously described (99, 100). At complete confluence, HTECs could be maintained under ALI conditions for 4–5 weeks at 37°C in a humidified 5% CO_2_ incubator, in accordance with previously described protocols (101).

### Fluorescence microscopy

Transwell membranes containing HTECs were fixed and permeabilized for 10–15 min with 100% chilled acetone then blocked with 5% bovine serum albumin (BSA). Unconjugated antibodies against Villin or β-tubulin (Invitrogen) were diluted in phosphate-buffered saline (PBS) with 2% BSA at titrated dilutions (typically 1:200) and incubated for 1 h at room temperature. The slides were then washed three times with PBS and incubated with the indicated secondary antibody. These included goat anti-mouse or anti-rabbit antibodies conjugated to Alexa 488 (Life Technologies; 1:5,000 dilution).

### Determination of sialic acid and cytokeratin distribution in HTECs by flow cytometry

Uninfected HTECs were first stained with biotinylated SNA lectin SA and MAA I FITC-conjugated lectin (Vector Laboratories). Biotinylated SNA was then visualized with a secondary streptavidin-BV786 (BD Biosciences). Cells were also separately fixed, permeabilized, and stained with the following conjugated monoclonal antibodies (Abcam) to measure CK expression: Alexa 647 anti-CK5 (ab193895), Alexa 647 anti-CK14 (ab192056), Alexa 647 anti-CK8/18 (ab17139), and PE anti-CK19 (ab216705). For all staining procedures, dead cells were excluded by Live-Dead Fixable Blue (ThermoFisher) staining according to the manufacturer’s protocol. Cell populations were gated based on unstained controls for the lectin evaluation and on matched isotype controls, as well as fluorescence-minus-one (FMO) staining, for the CK staining. All flow cytometric data were acquired using a FACSymphony A5 cytometer (BD) and analyzed using either FACSDiva or FlowJo software (BD).

### Virus replication kinetics

Primary HTECs were grown at the ALI and infected with different influenza viruses at an MOI of 0.1. Four human influenza viruses (A/HK/4801/2014 [H3N2], B/Brisbane/60/2008 [IB], A/TN/1–560/2009 [pH1N1], and A/WSN/1933 [H1N1]), two swine influenza viruses (A/swine/OH/16TOSU4783/2016 [H3N2] and A/swine/NC/18161/2002 [H1N1]), and two avian influenza viruses (A/Vietnam/1203/2004 [rgH5N1] and A/scaup/GA/W22-145E/2022 [H5N1]) were used.

Viruses used in this study were wildtype except for IB, H1N1, and rgH5N1, which were generated by reverse genetics. Approximately 6 × 10^5^ cells/well were washed three times with PBS and inoculated with 100 µL RPMI medium containing influenza viruses at an MOI of 0.1 from the apical side. After 2 h of incubation at 37°C, the HTECs were rinsed with PBS three times to remove unbound viral particles, and fresh TEpiCM-ALI supplemented with 0.3 µg/mL of TPCK-trypsin was added to the basal side. Infected HTECs were maintained under ALI conditions at 37°C in 5% CO_2_. At the indicated time points, 100 µL of RPMI medium with TPCK-treated trypsin (supplemented with 0.3 µg/mL of TPCK-trypsin) was added to each Transwell from the apical surface, incubating for a further 30 min at 37°C, then collecting for virus titration by TCID_50_ assay.

### Determination of viral replication dynamics in HTECs by flow cytometry

HTECs were grown at the ALI and infected with different influenza viruses at an MOI of 0.1. Two human influenza viruses (A/HK/4801/2014 [H3N2] and A/TN/1–560/2009 [pH1N1]) and two swine influenza viruses (A/swine/OH/16TOSU4783/2016 [H3N2] and A/swine/NC/18161/2002 [H1N1]) were used for this study. Cells were collected at the specified time points for staining with the indicated antibodies against CKs and influenza nucleoprotein (FluNP) as follows: infected HTECs were dissociated by scrapping, pipetting, and filtration. Cells were then stained with fixable live-dead blue (ThermoFisher) according to the manufacturer’s protocol, then fixed, and permeabilized using the BD Cytofix/Cytoperm kit and protocol. Next, cells were stained with FITC-conjugated anti-FluNP monoclonal antibody and with one of four conjugated anti-CK antibodies (PE anti-CK19, Alexa 647 anti-CK5, Alexa 647 anti-CK14, and Alexa 647 anti-Ck8/18). Flow cytometric analysis was followed using a BD FACSymphony A5 for data acquisition and FASCDiva or FlowJo software for analysis. Cell populations were gated for CK and NP expression based on isotype and FMO controls.

### Scanning electron microscopy

SEM was performed as described previously (14). Briefly, cells grown in culture on membranes were fixed with 2.5% glutaraldehyde for 24 h and then treated with 1% osmium tetroxide for 2 h. This was followed by dehydration in an ascending dilution series of ethanol and drying with a Polaron E3000 critical point dryer. The cells were then adhered to stubs, sputter coated, and examined with a Zeiss DSM940 scanning electron microscope.

### Cytokine quantification

Forty-eight cytokines, chemokines, and growth factors were measured in the supernatants from HTECs infected with one of six selected viruses: A/HK/4801/2014 (H3N2), A/TN/1–560/2009 (pH1N1), A/WSN/1933 (H1N1), A/swine/OH/16TOSU4783/2016 (H3N2), A/swine/NC/18161/2002 (H1N1), and A/Vietnam/1203/2004 (rgH5N1). At the indicated time points of infections, 100 µL of RPMI medium was added to each Transwell insert, incubated for 30 minutes, and then the media and cells were collected. Multiplexed cytokine assays were performed using a Bio-Plex Pro Human cytokine screening panel (Bio-Rad) containing 48 human cytokines according to the manufacturer’s instructions, and the spectral intensities were quantified with a Luminex instrument. Cytokine concentrations were calculated by interpolating values from a standard curve via curve fitting. Samples with values below the detection limit were assigned a value of 1 to enable the use of a log scale for calculating fold changes and graphing.

### Statistical analyses

All data are shown as the mean ± SD unless stated otherwise. Data analyses were performed with GraphPad Prism 10 software and R version 4.1.0. All cytokine data were normalized and log2 transformed. Principle component analysis was applied for dimensional reduction and data visualization of cytokine responses. The changes in cytokine response relative to that in mock-infected cells were modeled by using a Gaussian generalized linear model in R (https://www.r-project.org).

### Biocontainment

This study utilized USDA-classified select agents. During the time in which this study was conducted, A(H5N1) viruses were subject to the requirements discussed in 9 CFR Parts 121 (Possession, Use, and Transfer of Select Agent Toxins) and 122 (Importation and Transportation of Controlled Organisms and Vectors).

## Data Availability

All data used for analysis and figure generation are available publicly at https://github.com/kvegesan-stjude/jvidata_faten. Raw FCS data are available from the authors upon request.

## SUPPLEMENTAL MATERIAL

File S1 jvi.01460-24-s0001.pdf Longitudinal cytokine values based on influenza virus strain infection.

Supplemental figures jvi.01460-24-s0002.docx Figures S1 to S6 and legends for all supplemental material.

Table S1 jvi.01460-24-s0003.docx Linear regression on log2 fold changes for each cytokine, with virus and time as independent variables.

Video S1 jvi.01460-24-s0004.mp4 Complete Z-stack from top to bottom (stalk of 10 layers; step size 412 nm).

Video S2 jvi.01460-24-s0005.mp4 Complete Z-stack from bottom to top (stalk of 12 layers; step size 412 nm).

ASM does not own the copyrights to Supplemental Material that may be linked to, or accessed through, an article. The authors have granted ASM a non-exclusive, world-wide license to publish the Supplemental Material files. Please contact the corresponding author directly for reuse.
