# Supplementary material for: Modulation of cytokeratin and cytokine/chemokine expression following influenza virus infection of differentiated human tonsillar epithelial cells
Source: J Virol. 2025 Jan 10;99(2):e01460-24. doi: 10.1128/jvi.01460-24 (PMC11852761; doi:10.1128/jvi.01460-24)
Supplement: File S1 — Longitudinal cytokine values based on influenza virus strain infection. [file jvi.01460-24-s0001.pdf]

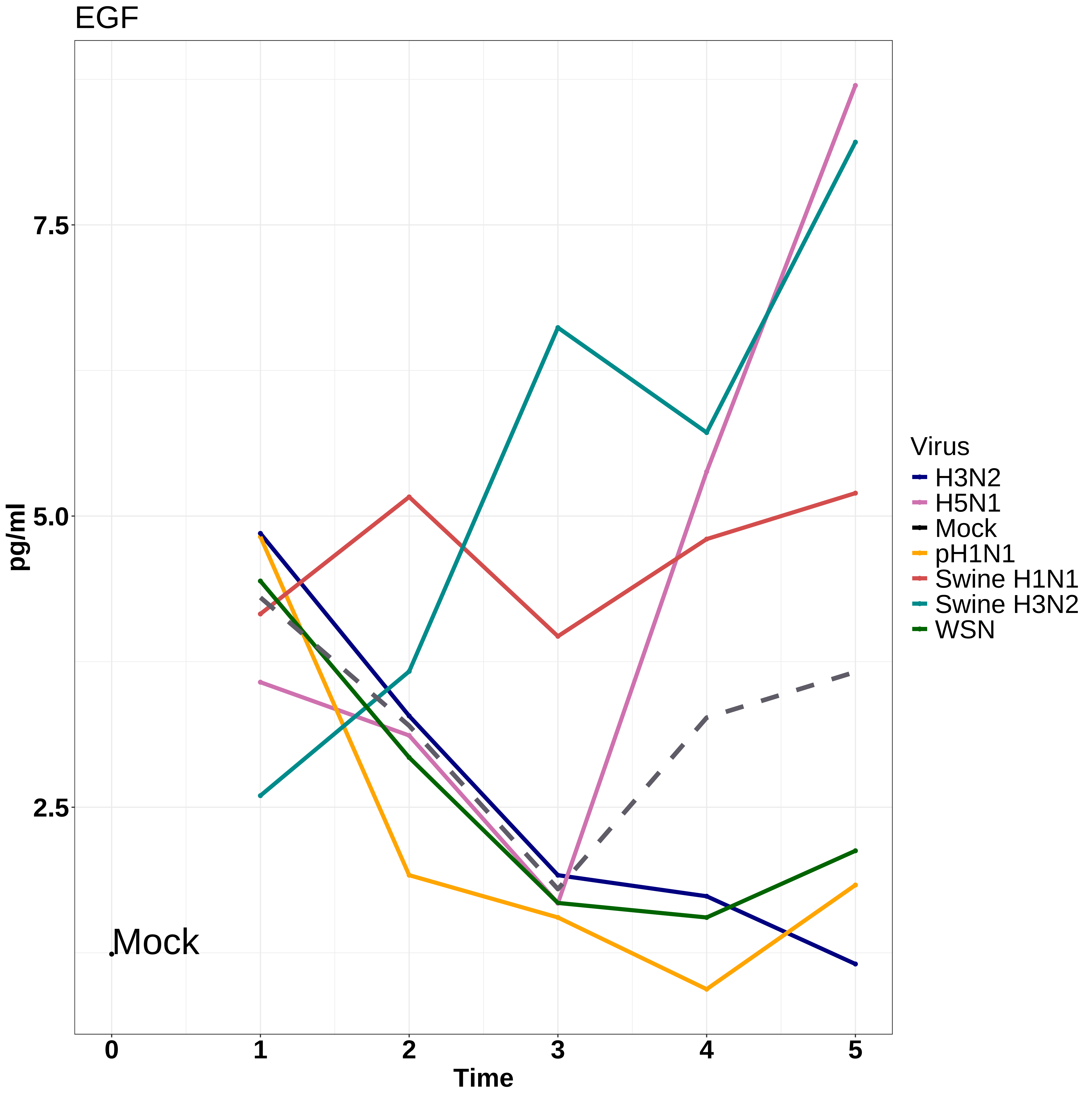
EGF
7.5
5.0
2.5
pg/ml
0
1
2
3
4
5
Time
Mock
Virus
H3N2
H5N1
Mock
pH1N1
Swine H1N1
Swine H3N2
WSN

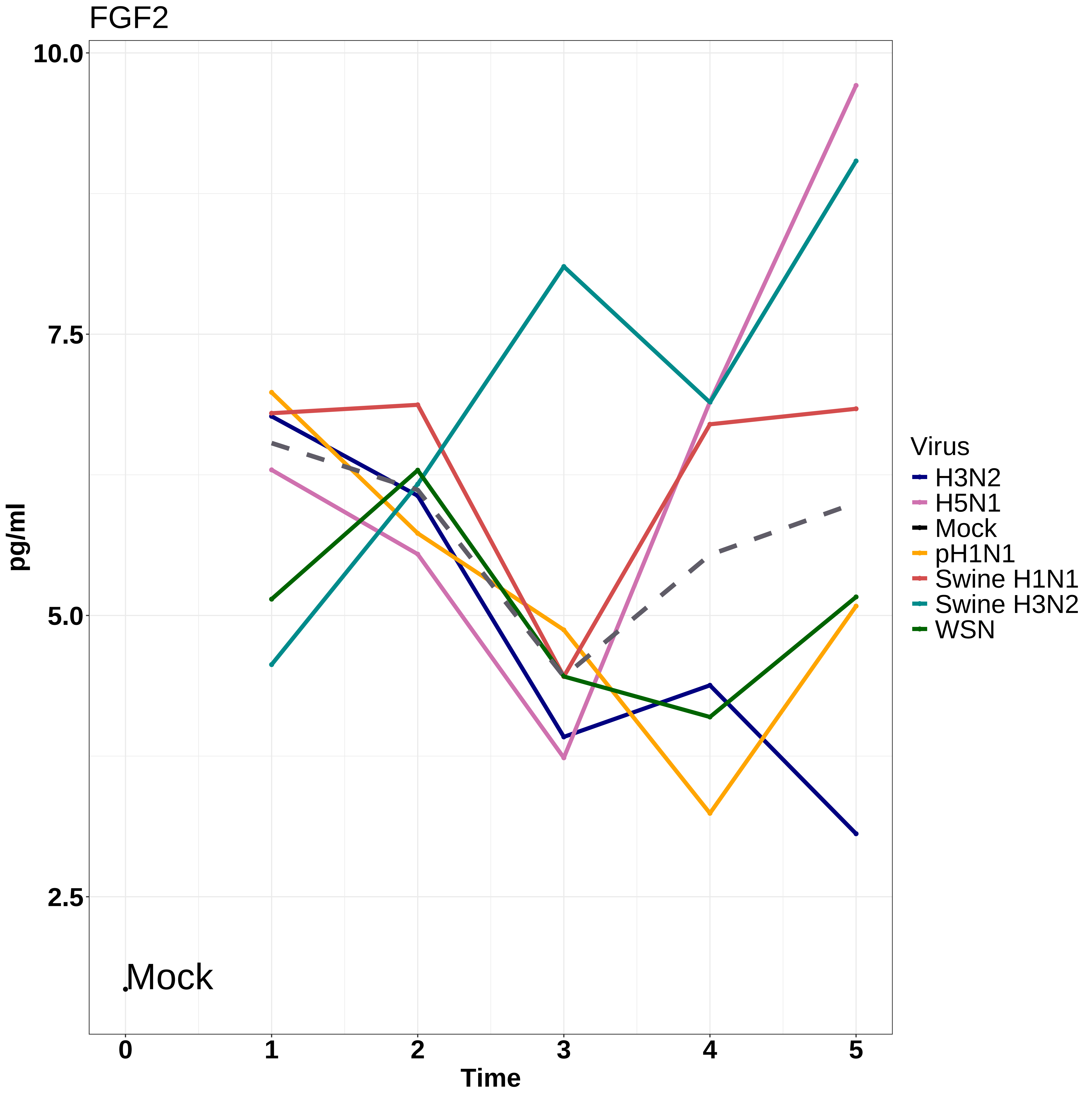
FGF2
10.0
7.5
5.0
2.5
pg/ml
0
1
2
3
4
5
Time
Mock
Virus
H3N2
H5N1
Mock
pH1N1
Swine H1N1
Swine H3N2
WSN

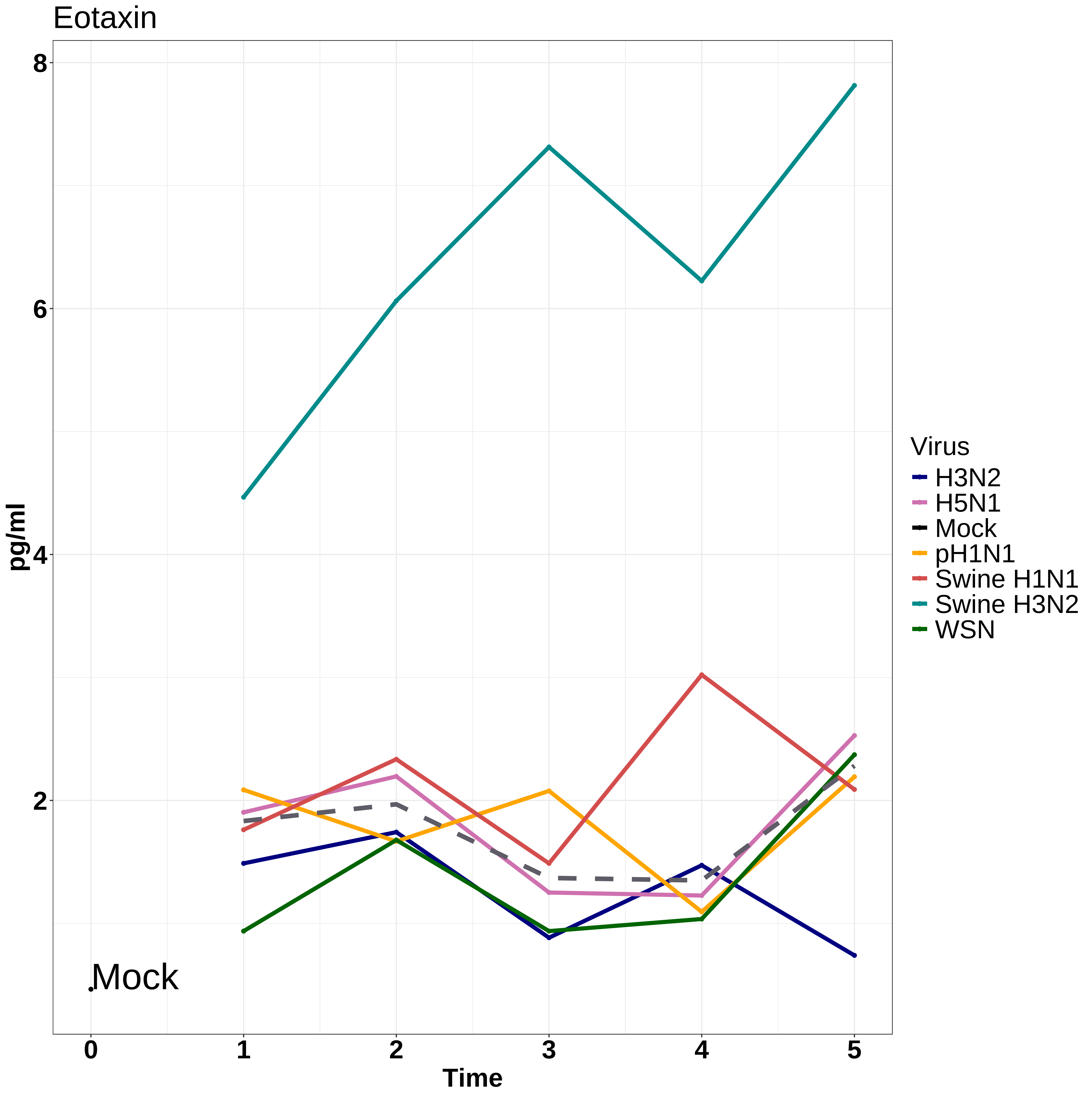

Eotaxin
8
6
4
2
pg/ml
Mock
0
1
2
3
4
5
Time
Virus
H3N2
H5N1
Mock
pH1N1
Swine H1N1
Swine H3N2
WSN

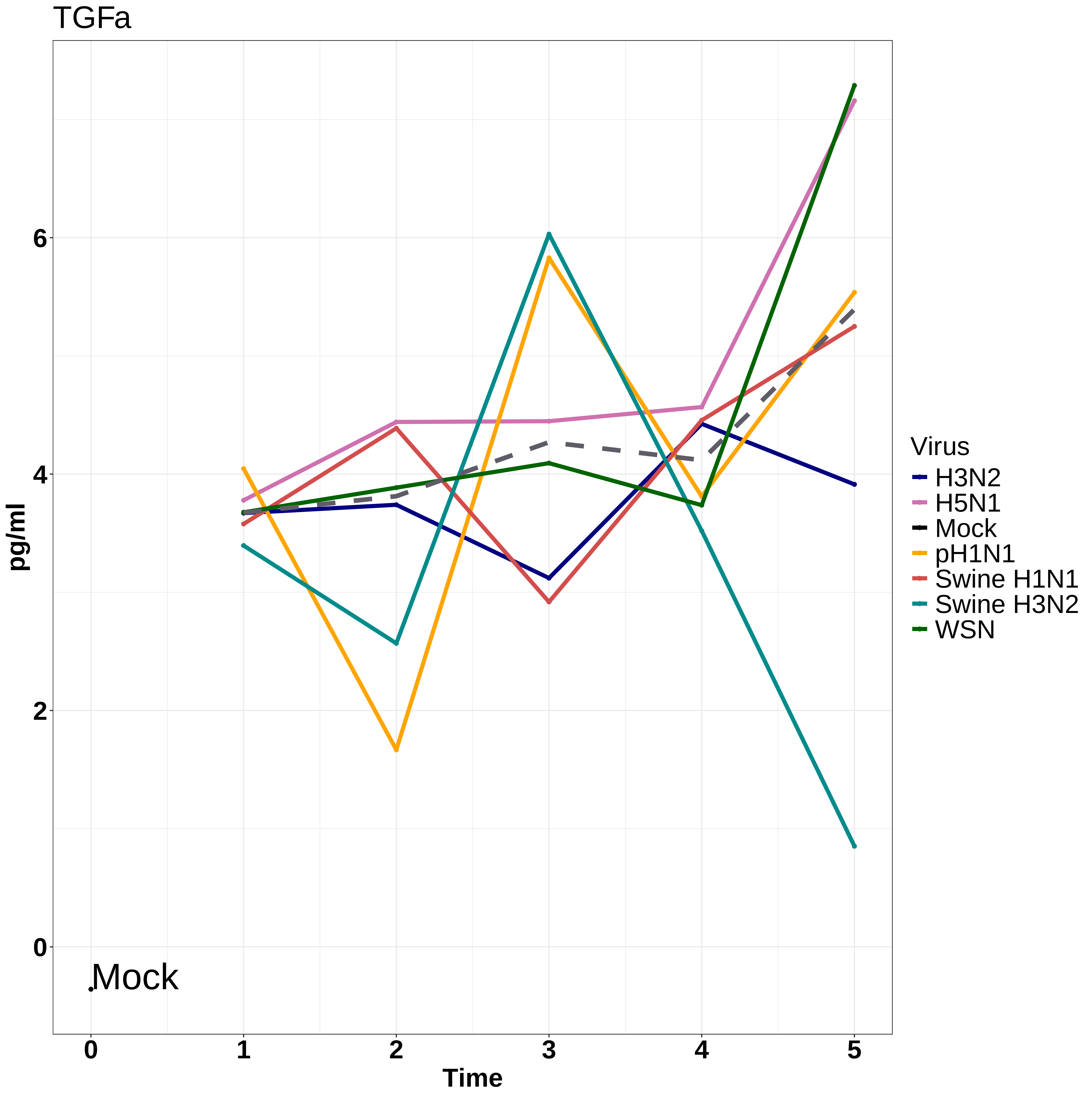

TGFa
pg/ml
6
4
2
0
0
1
2
3
4
5
Time
Mock
Virus
H3N2
H5N1
Mock
pH1N1
Swine H1N1
Swine H3N2
WSN

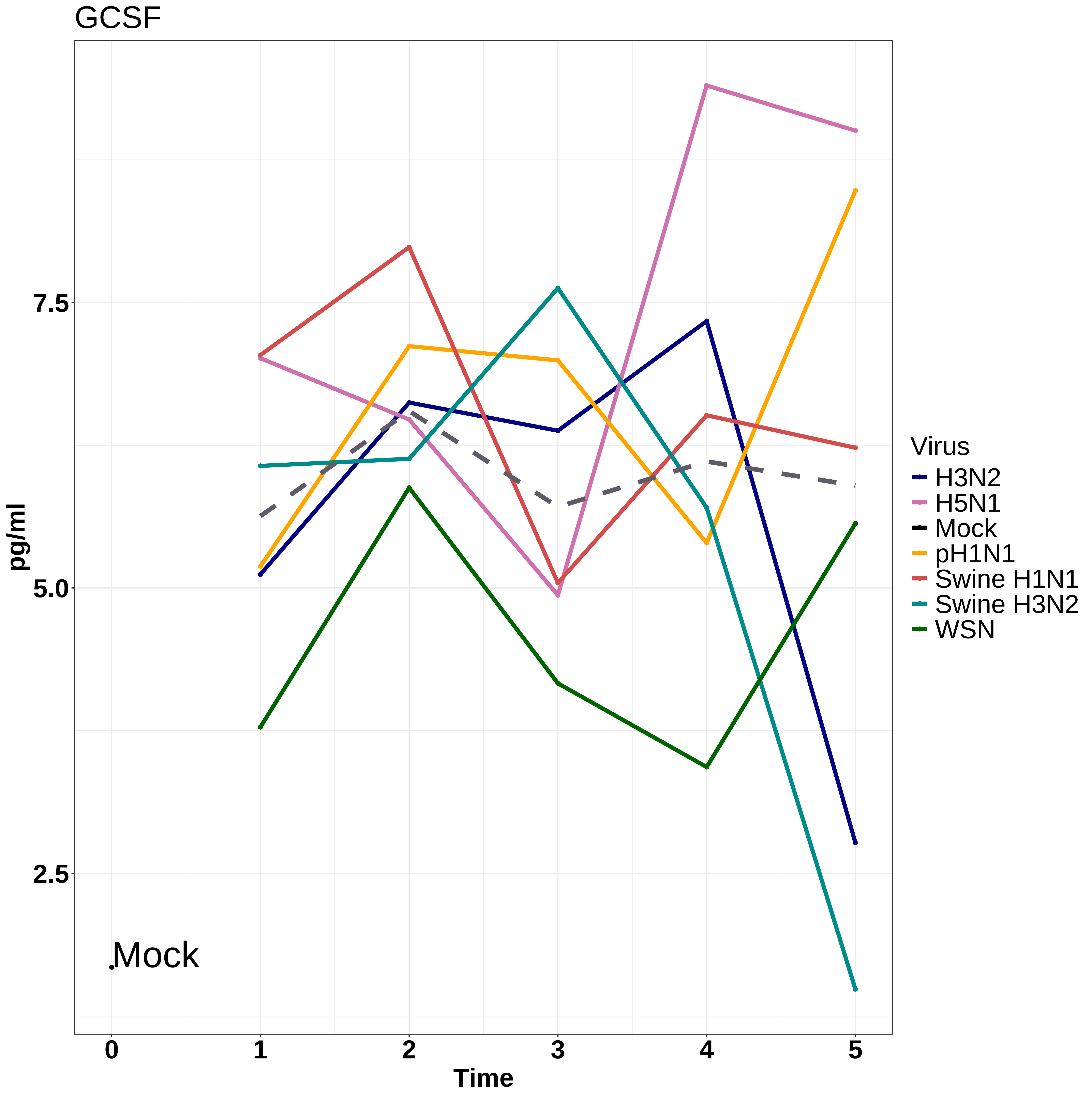
GCSF
pg/ml
7.5
5.0
2.5
0
1
2
3
4
5
Time
Mock
Virus
H3N2
H5N1
Mock
pH1N1
Swine H1N1
Swine H3N2
WSN

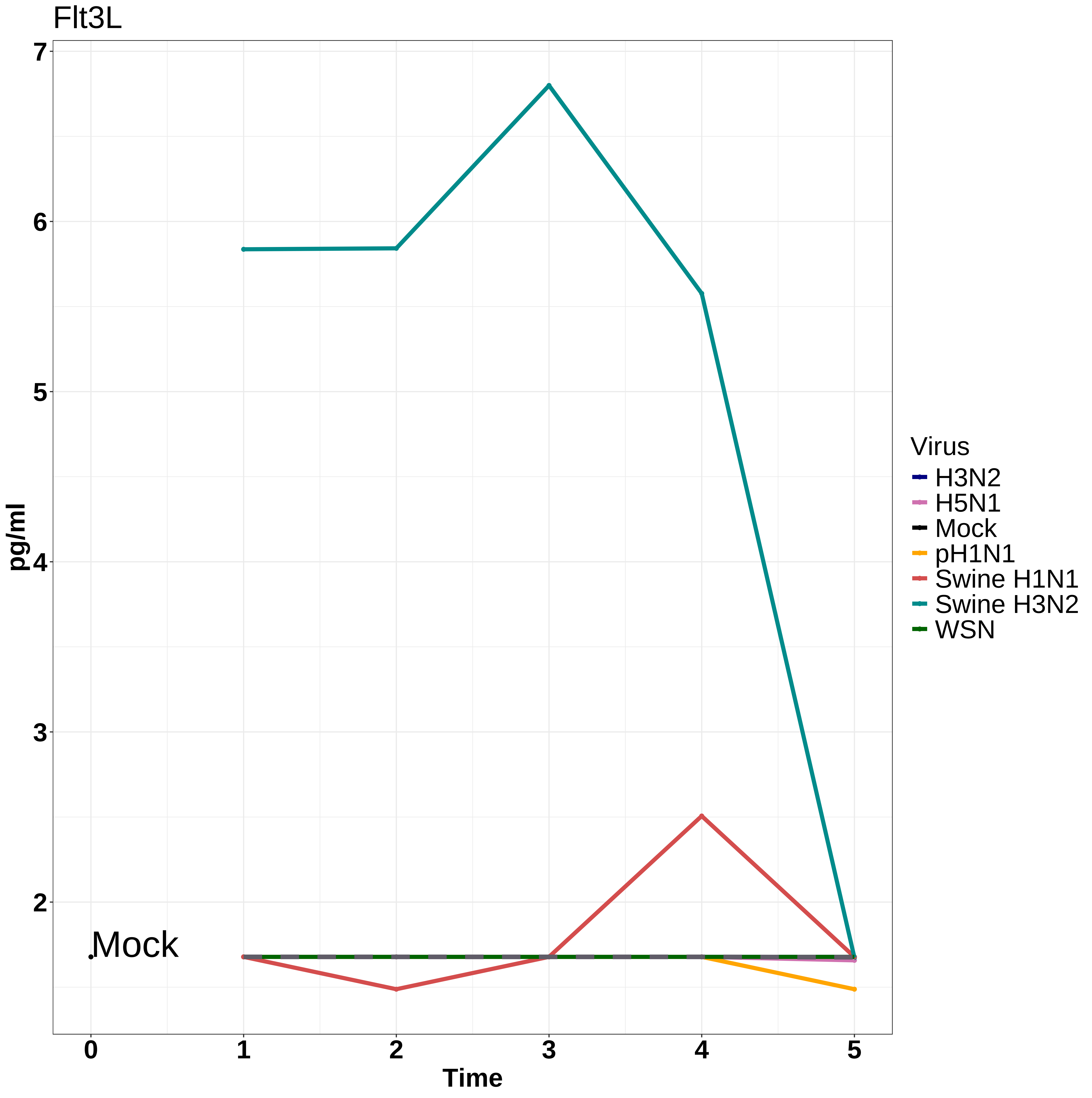

Flt3L
pg/ml
Time
Mock
Virus
H3N2
H5N1
Mock
pH1N1
Swine H1N1
Swine H3N2
WSN

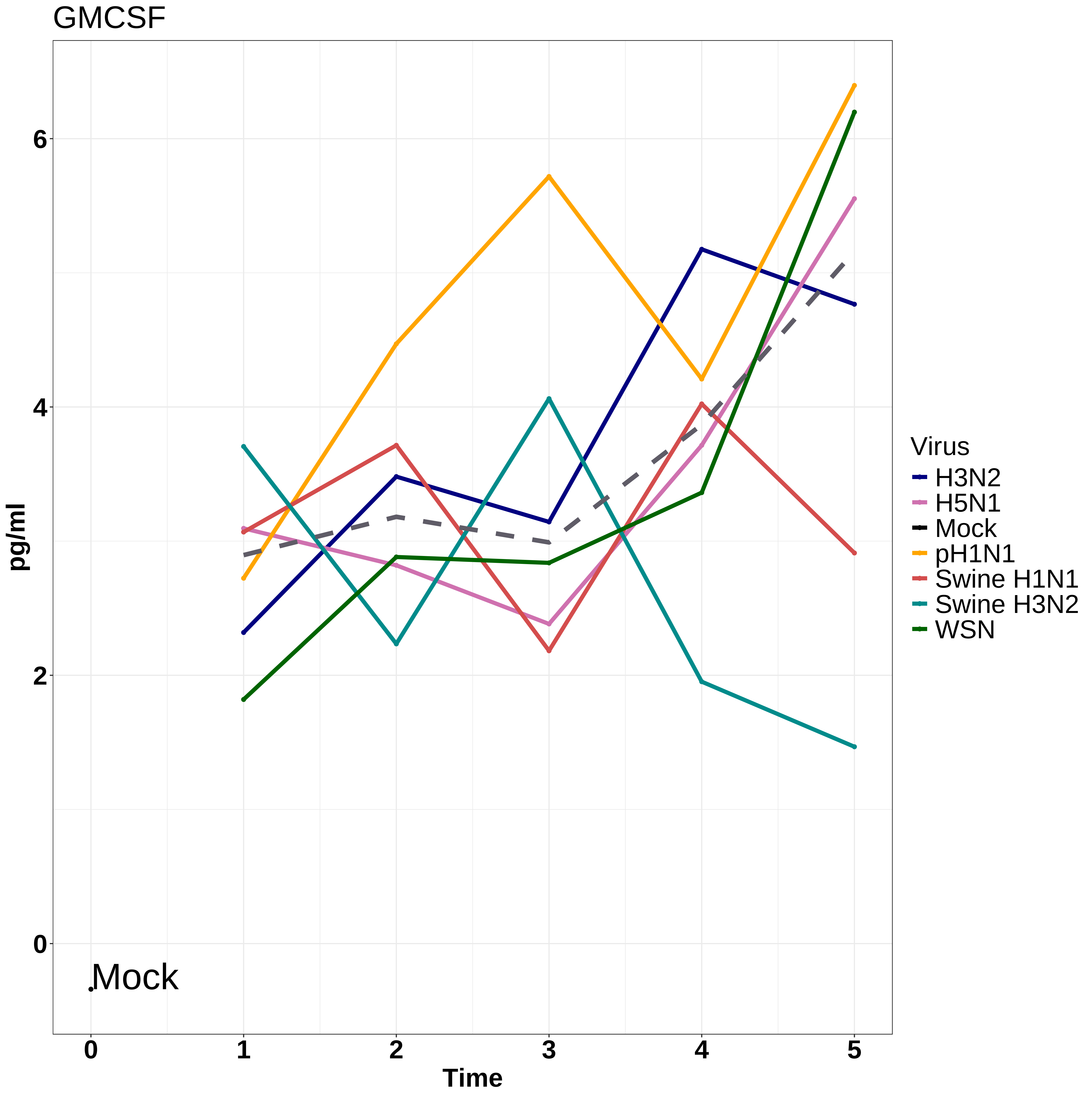
GMCSF
pg/ml
Time
0
2
4
6
0
1
2
3
4
5
Mock
Virus
H3N2
H5N1
Mock
pH1N1
Swine H1N1
Swine H3N2
WSN

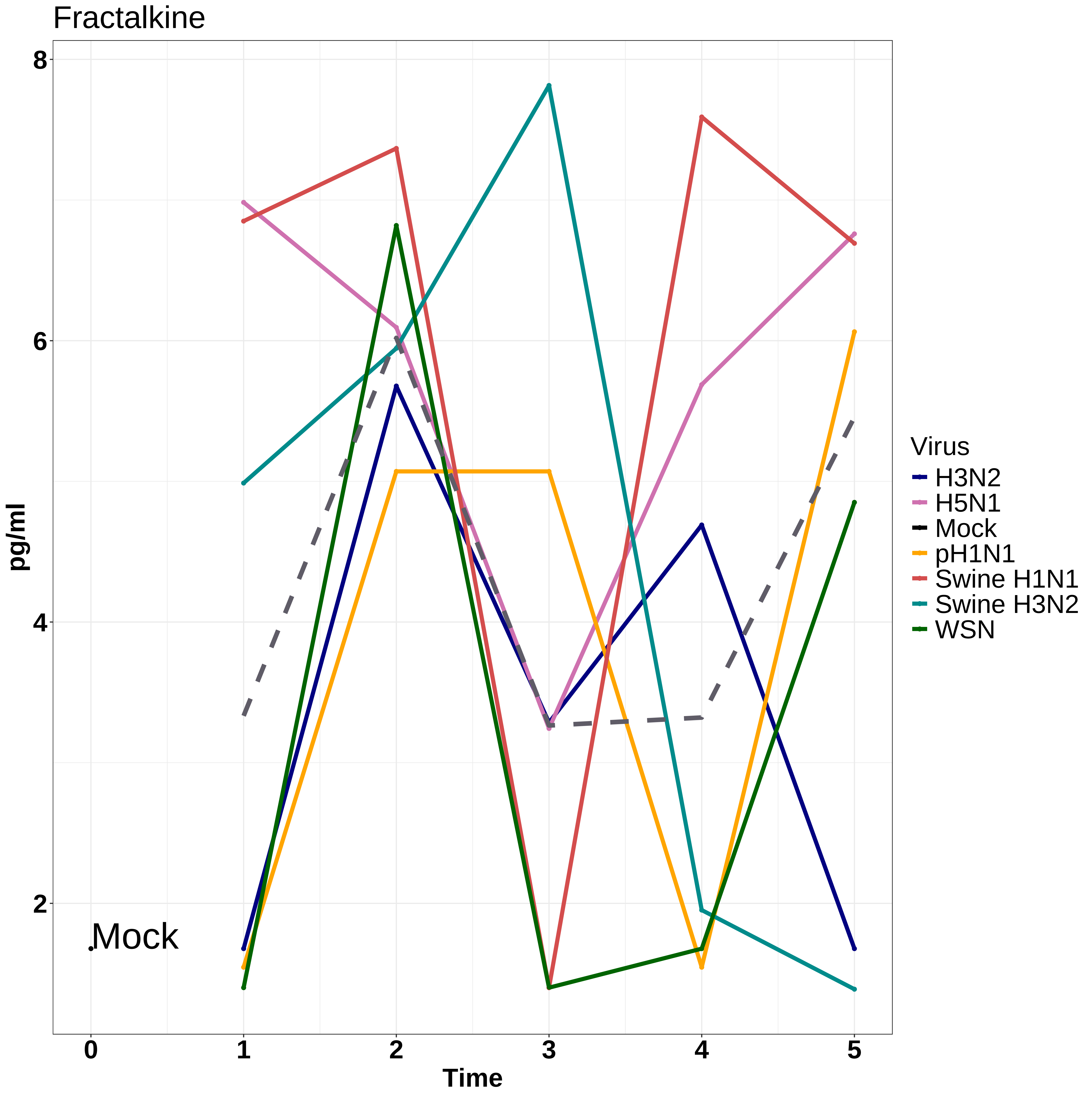

Fractalkine
pg/ml
8
6
4
2
Mock
0
1
2
3
4
5
Time
Virus
H3N2
H5N1
Mock
pH1N1
Swine H1N1
Swine H3N2
WSN

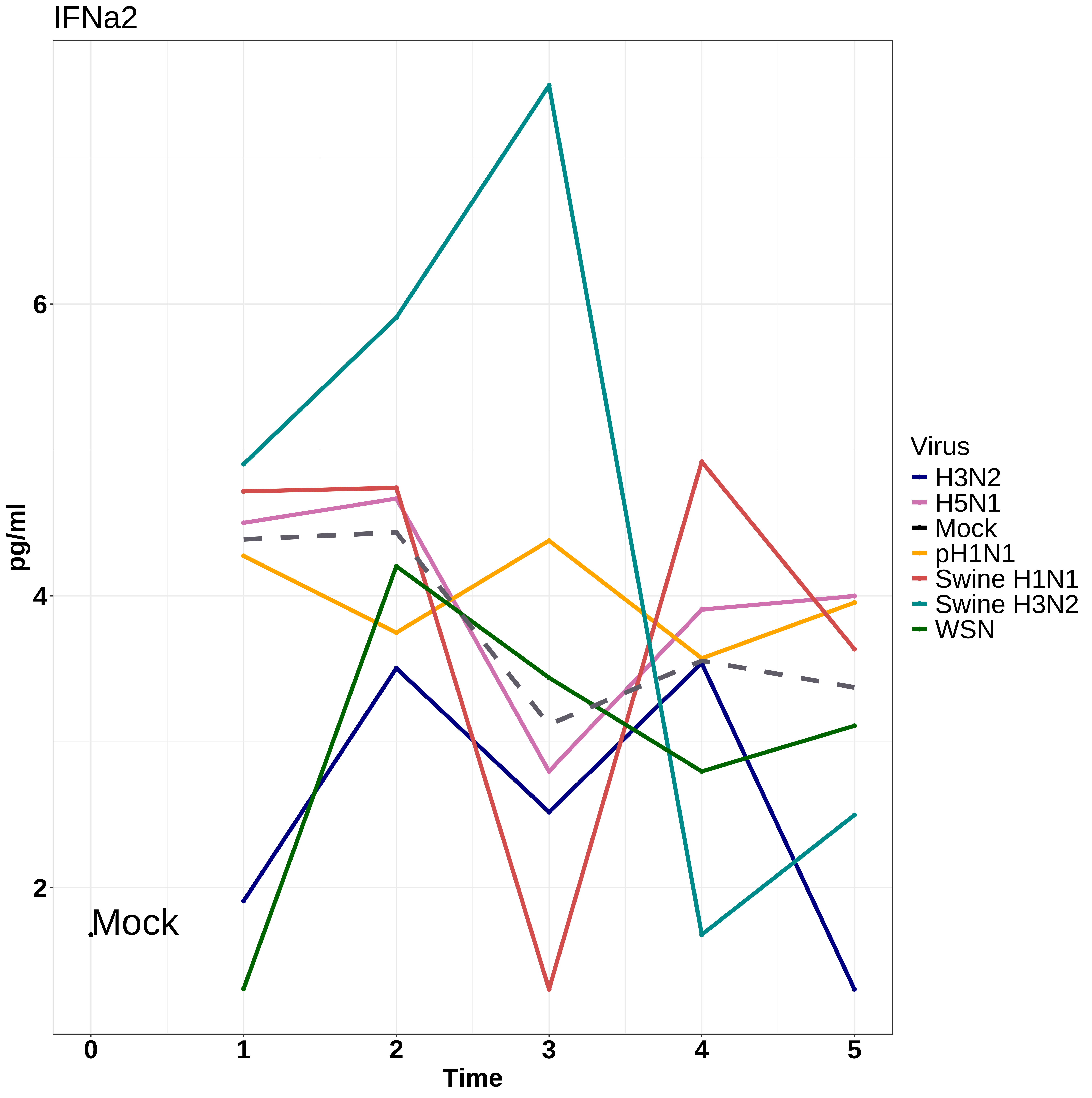
IFNa2
pg/ml
6
4
2
0
1
2
3
4
5
Time
Mock
Virus
H3N2
H5N1
Mock
pH1N1
Swine H1N1
Swine H3N2
WSN

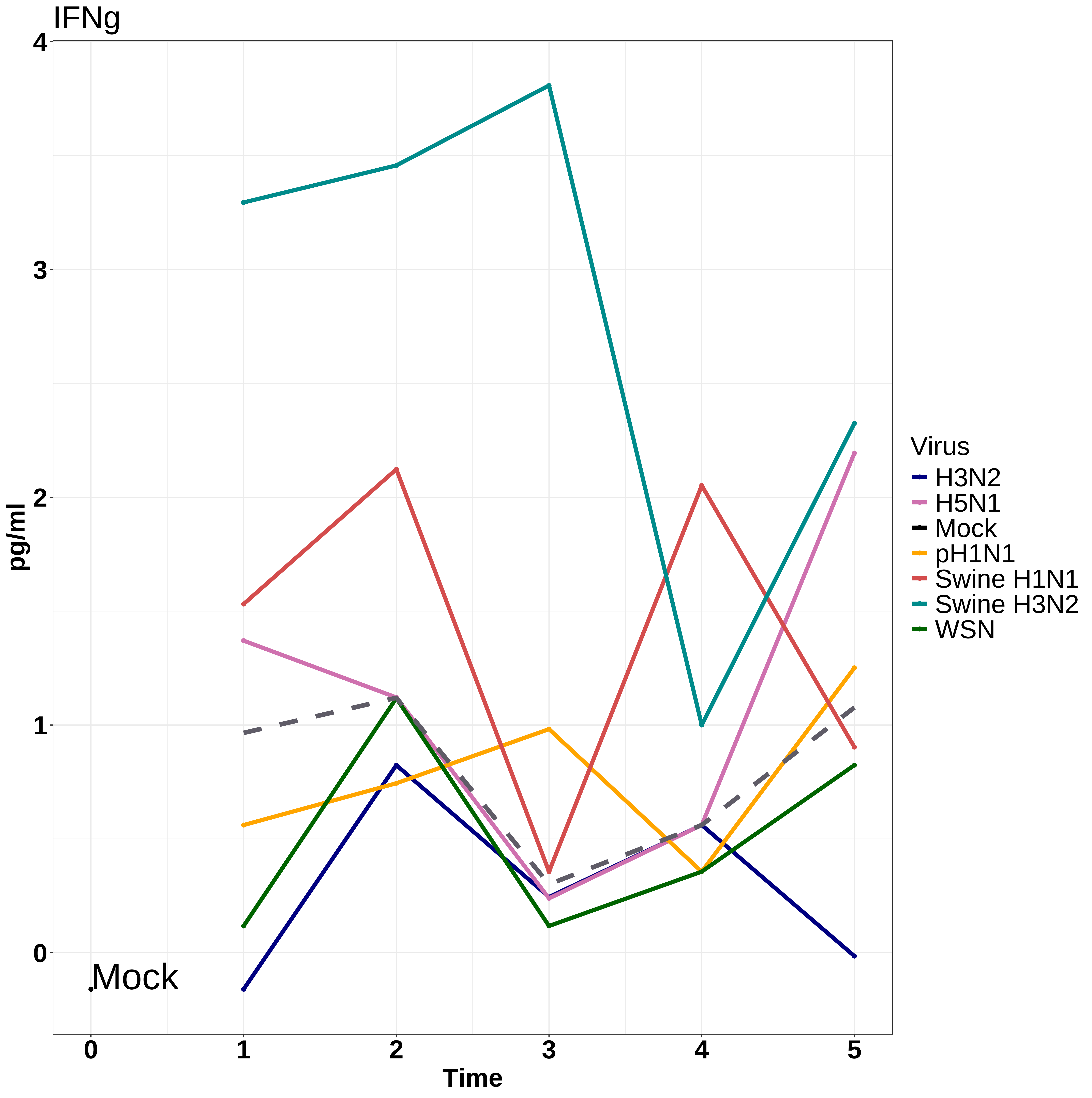
IFNg
pg/ml
Time
0
1
2
3
4
5
Mock
Virus
H3N2
H5N1
Mock
pH1N1
Swine H1N1
Swine H3N2
WSN

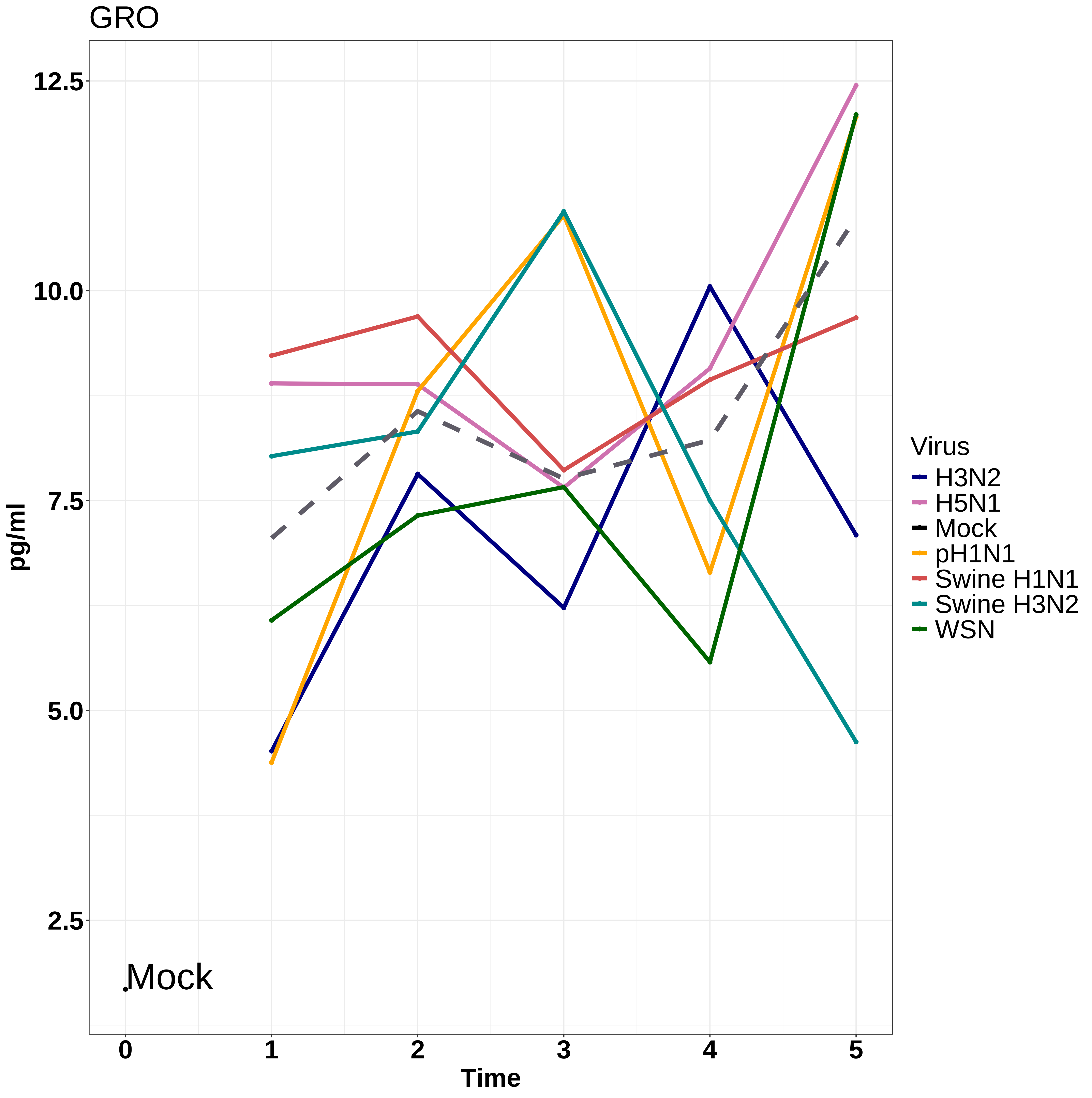
GRO
12.5
10.0
7.5
5.0
2.5
pg/ml
0
1
2
3
4
5
Time
Mock
Virus
H3N2
H5N1
Mock
pH1N1
Swine H1N1
Swine H3N2
WSN

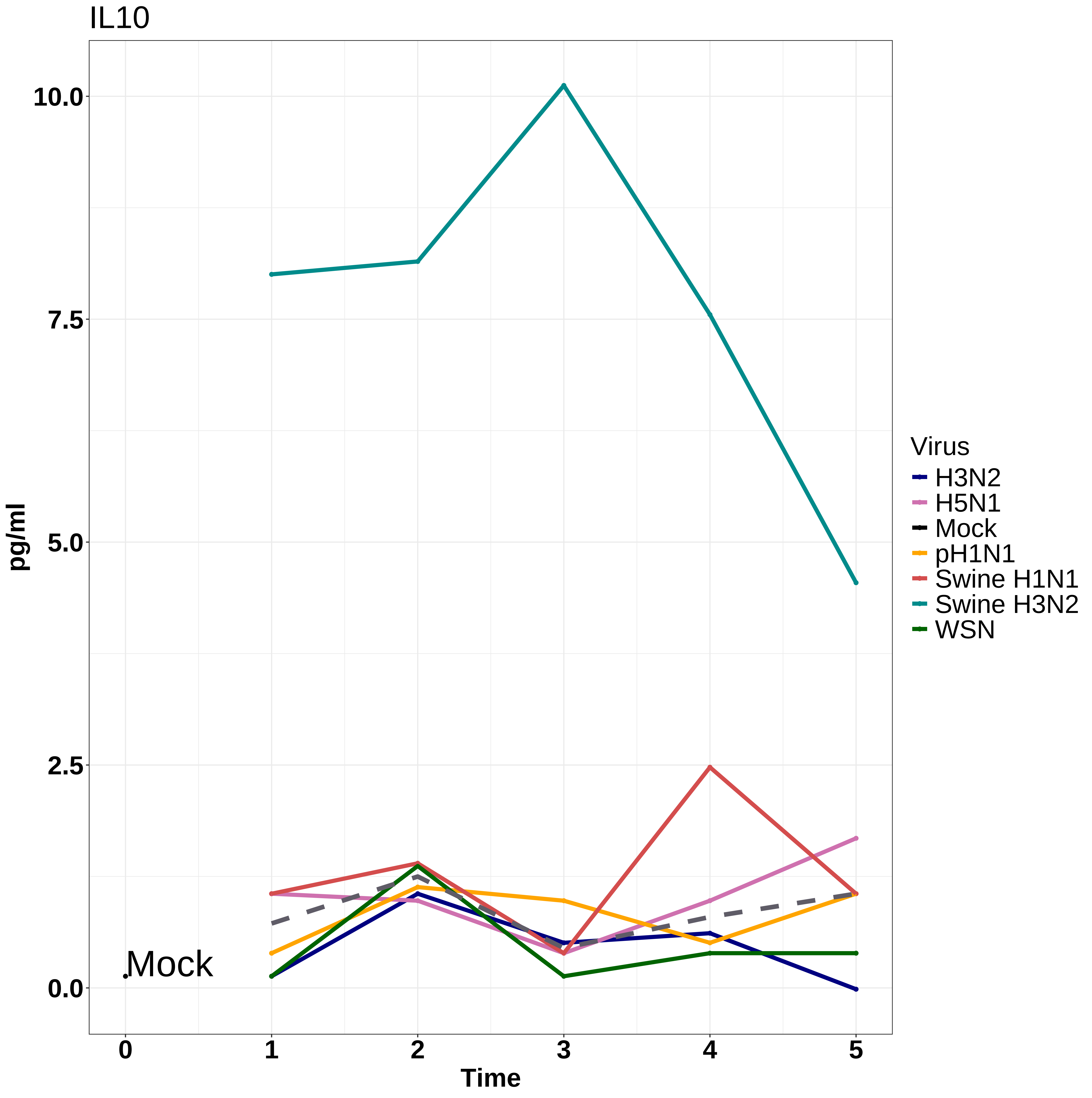
IL10
10.0
7.5
5.0
2.5
0.0
pg/ml
Mock
0
1
2
3
4
5
Time
Virus
H3N2
H5N1
Mock
pH1N1
Swine H1N1
Swine H3N2
WSN

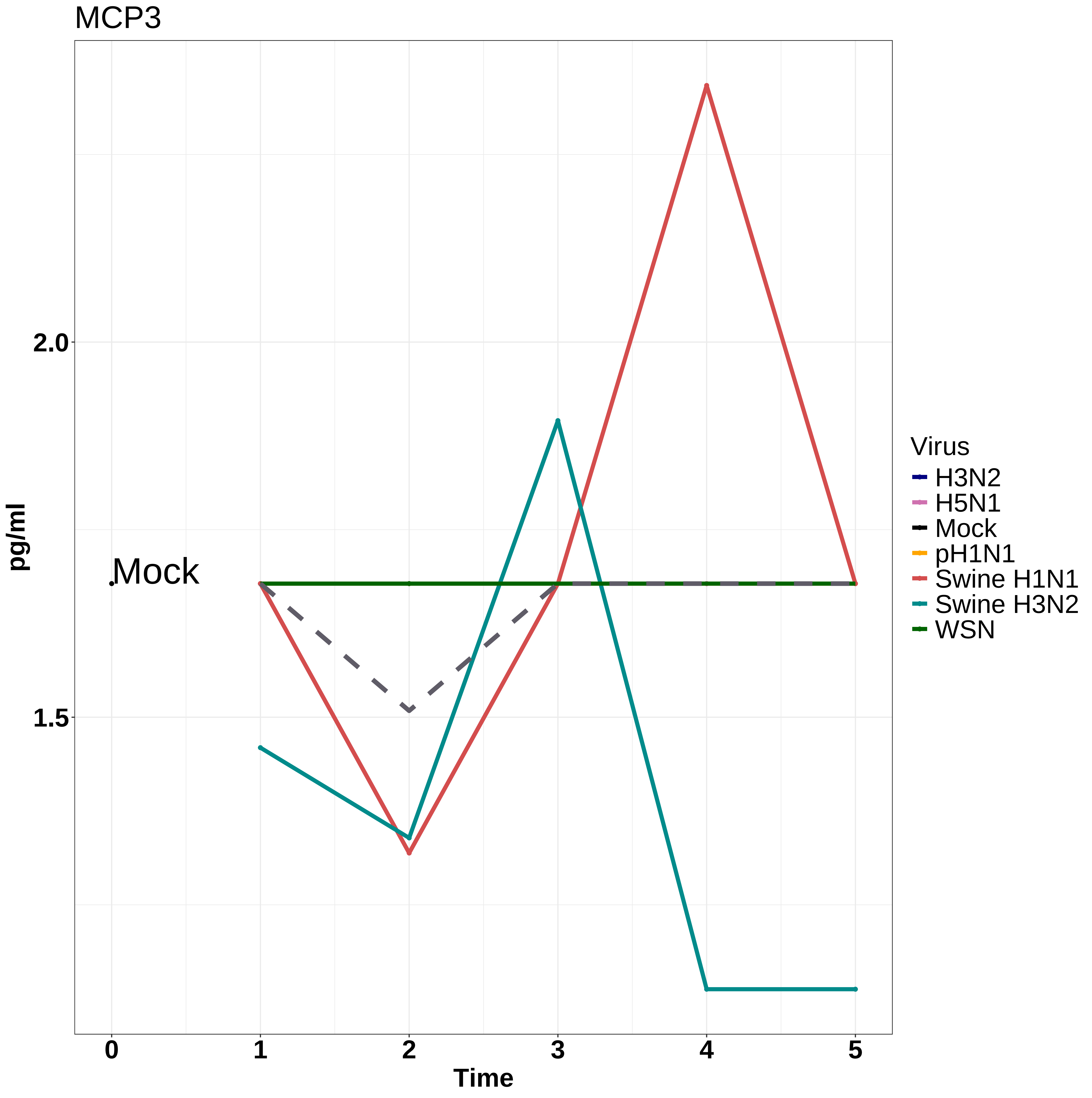
MCP3
pg/ml
2.0
1.5
Mock
0
1
2
3
4
5
Time
Virus
H3N2
H5N1
Mock
pH1N1
Swine H1N1
Swine H3N2
WSN

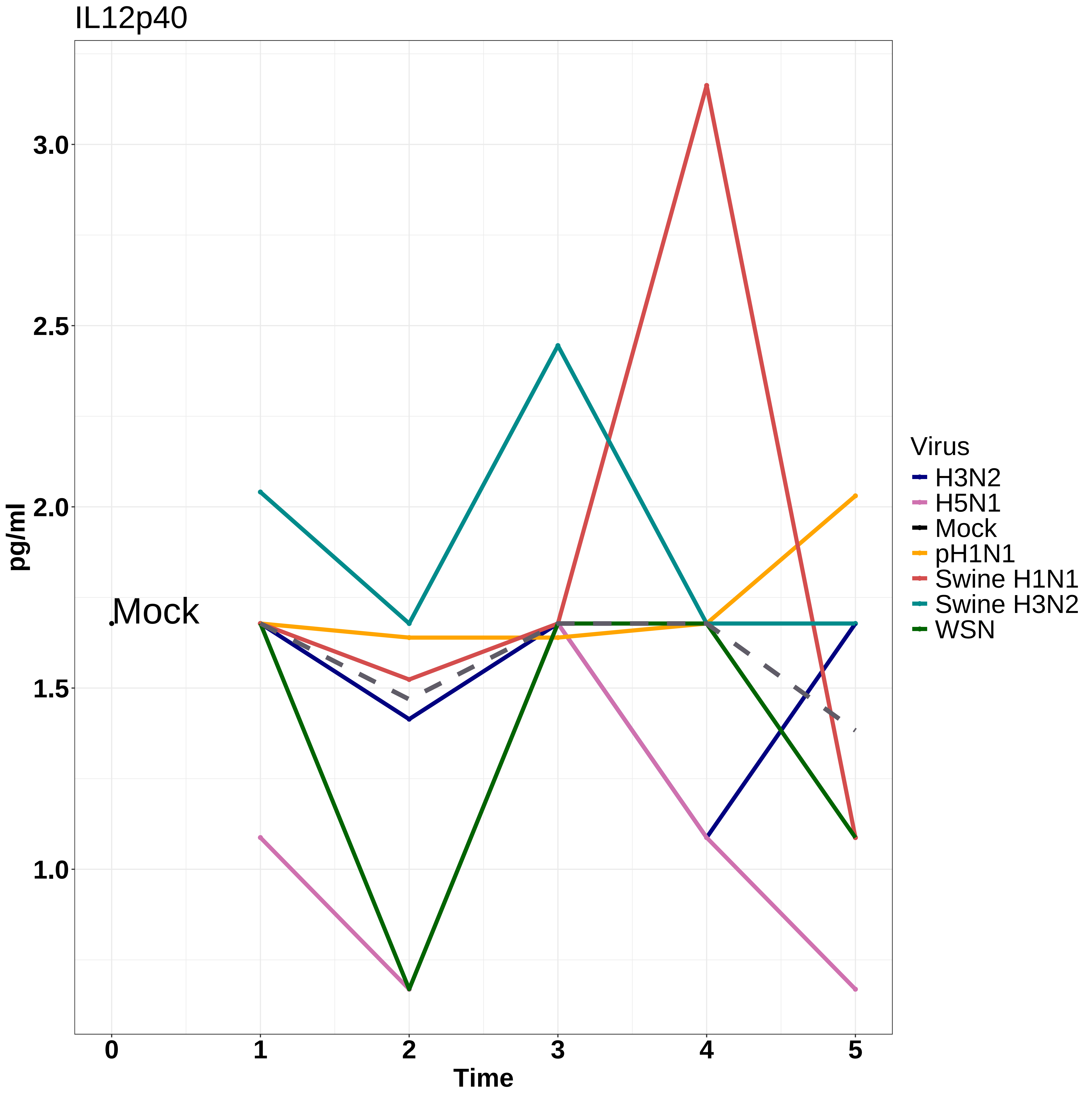
IL12p40
pg/ml
3.0
2.5
2.0
1.5
1.0
Mock
0
1
2
3
4
5
Time
Virus
H3N2
H5N1
Mock
pH1N1
Swine H1N1
Swine H3N2
WSN

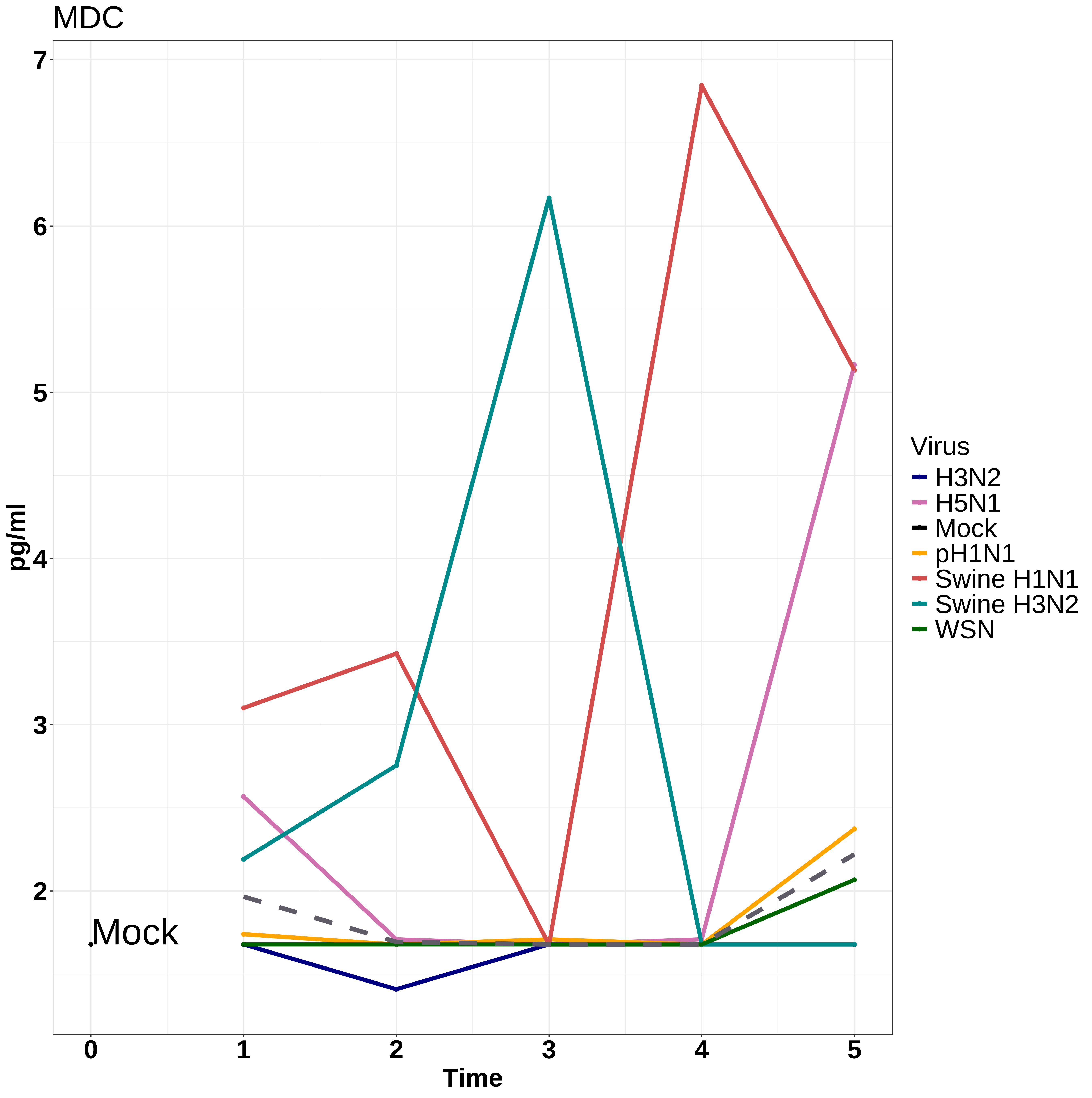
MDC
pg/ml
7
6
5
4
3
2
Mock
0
1
2
3
4
5
Time
Virus
H3N2
H5N1
Mock
pH1N1
Swine H1N1
Swine H3N2
WSN

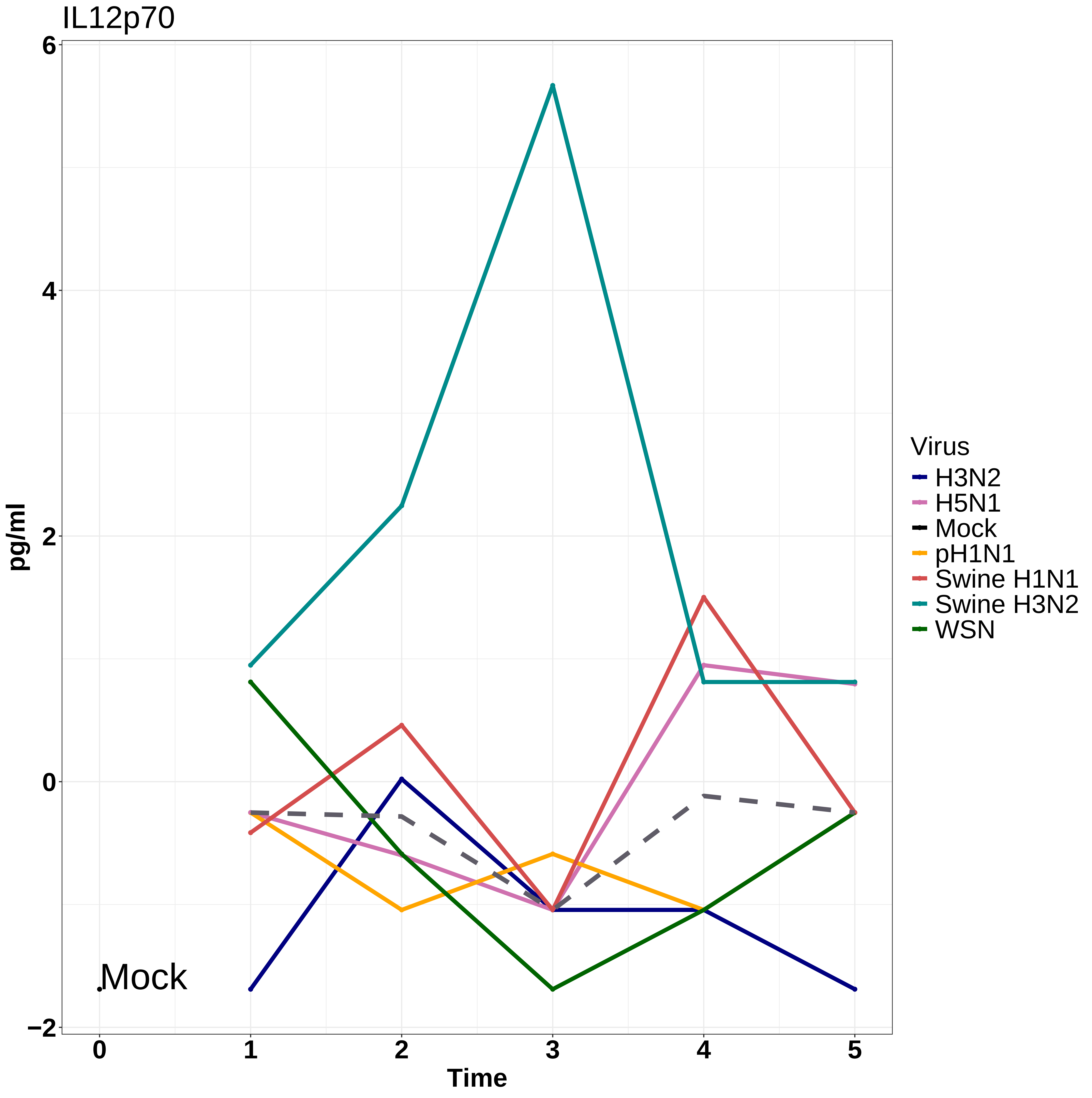
IL12p70
pg/ml
6
4
2
0
−2
Time
0
1
2
3
4
5
Mock
Virus
H3N2
H5N1
Mock
pH1N1
Swine H1N1
Swine H3N2
WSN

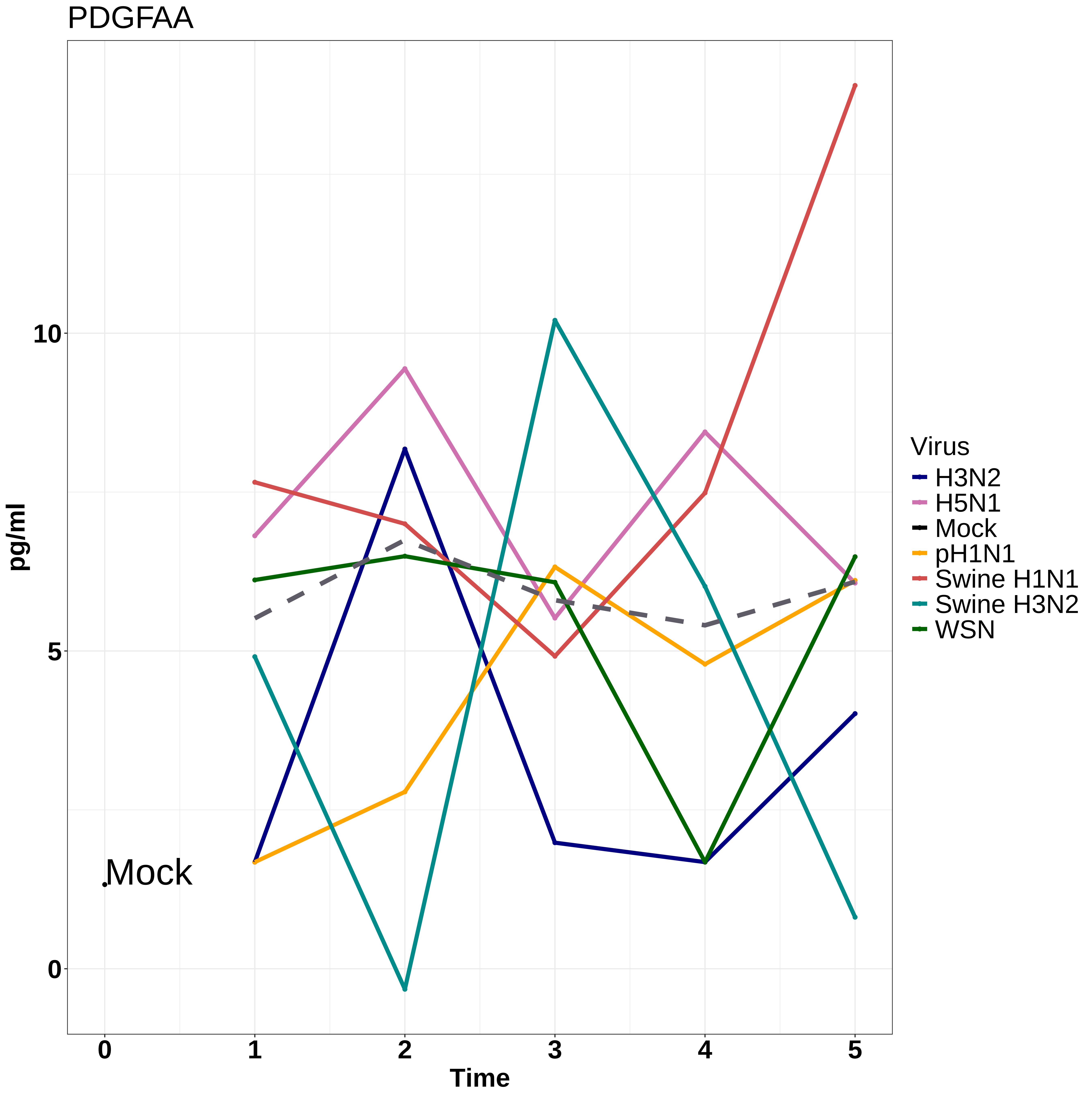

PDGFAA
pg/ml
10
5
0
Mock
0
1
2
3
4
5
Time
Virus
H3N2
H5N1
Mock
pH1N1
Swine H1N1
Swine H3N2
WSN

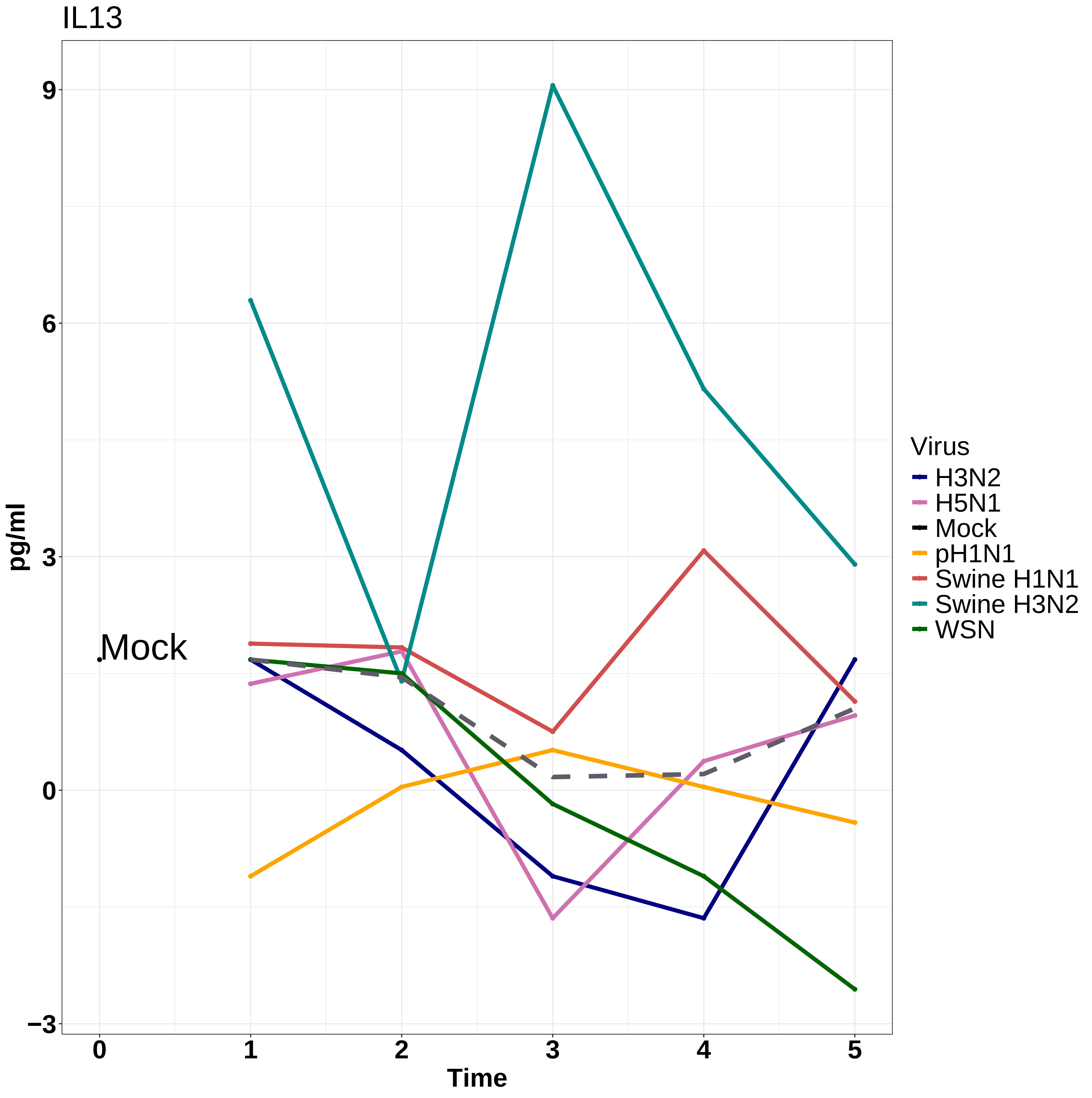

IL13
9
6
3
0
−3
pg/ml
0
1
2
3
4
5
Time
Mock
Virus
H3N2
H5N1
Mock
pH1N1
Swine H1N1
Swine H3N2
WSN

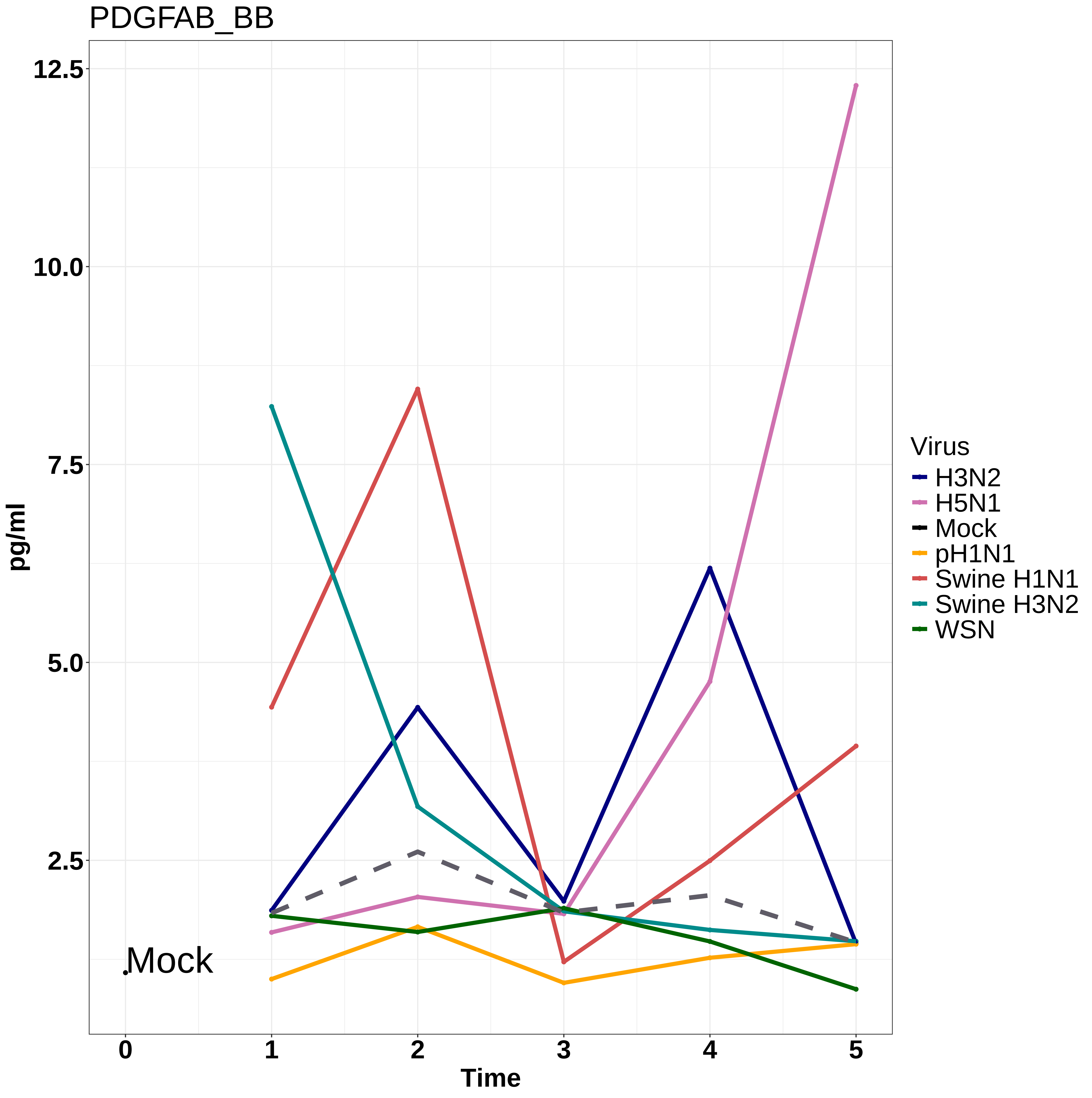
PDGFAB_BB
pg/ml
12.5
10.0
7.5
5.0
2.5
0
1
2
3
4
5
Time
Mock
Virus
H3N2
H5N1
Mock
pH1N1
Swine H1N1
Swine H3N2
WSN

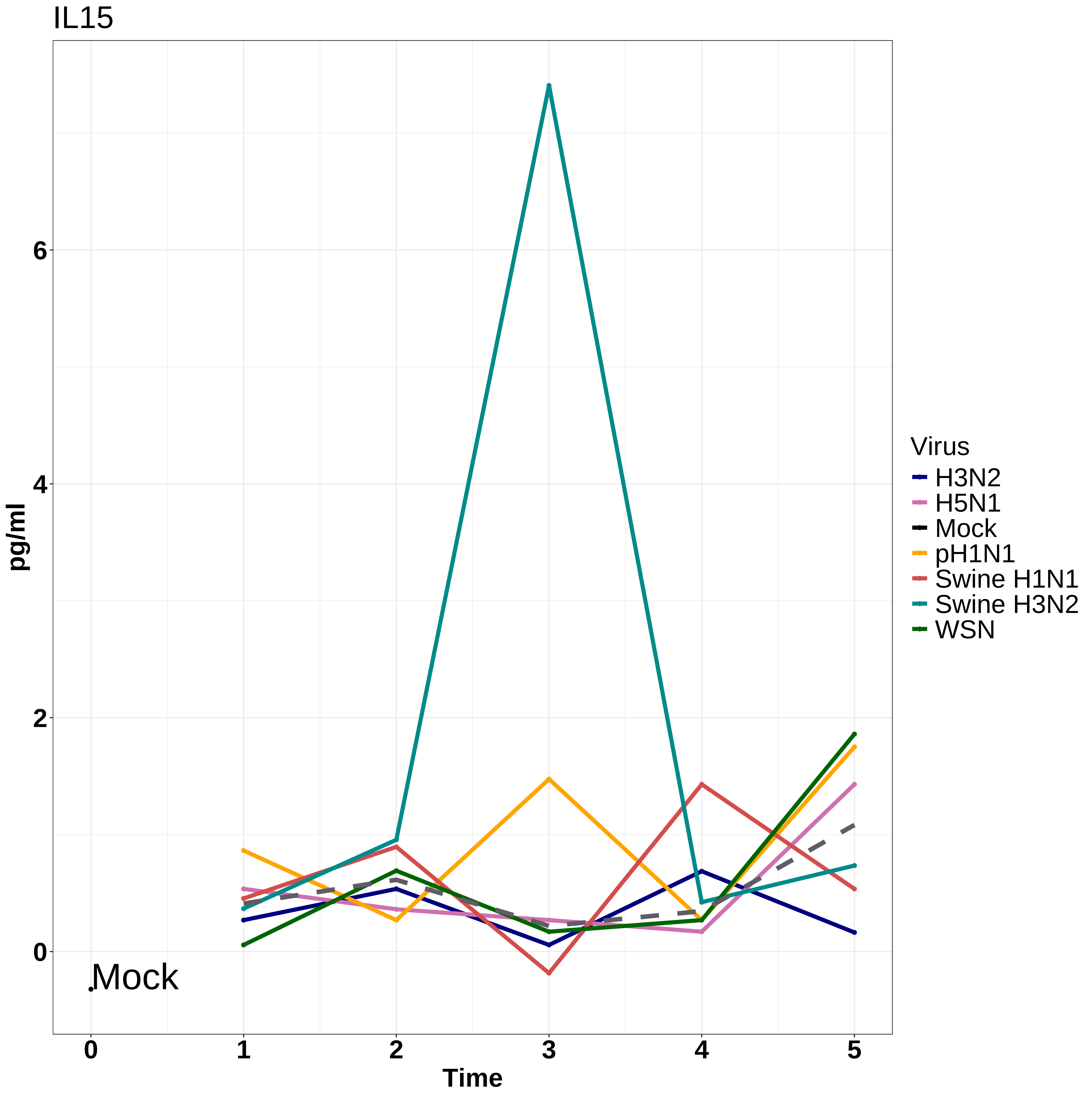
IL15
pg/ml
6
4
2
0
Mock
0
1
2
3
4
5
Time
Virus
H3N2
H5N1
Mock
pH1N1
Swine H1N1
Swine H3N2
WSN

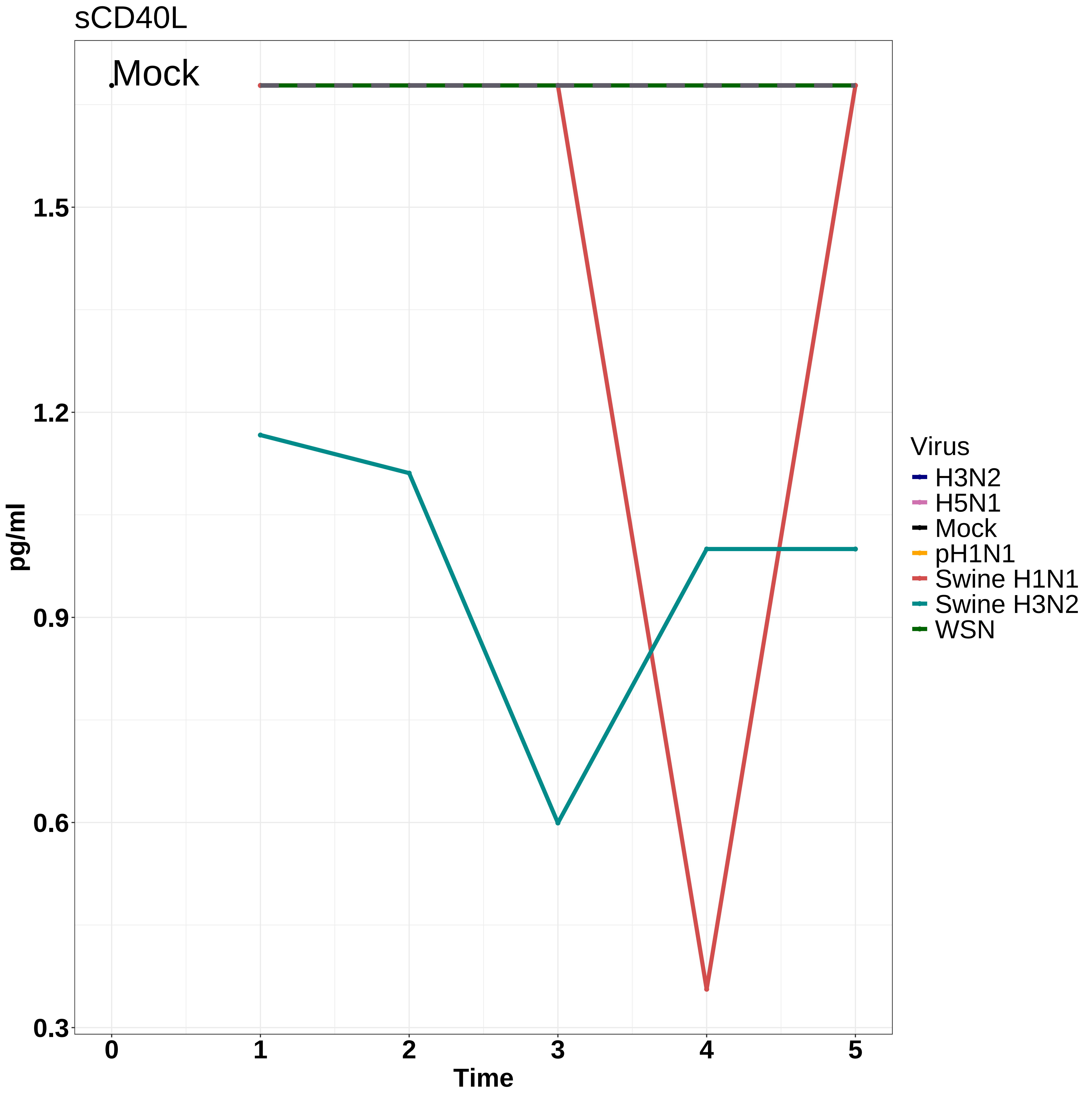

sCD40L
Mock
1.5
1.2
0.9
0.6
0.3
pg/ml
0
1
2
3
4
5
Time
Virus
H3N2
H5N1
Mock
pH1N1
Swine H1N1
Swine H3N2
WSN

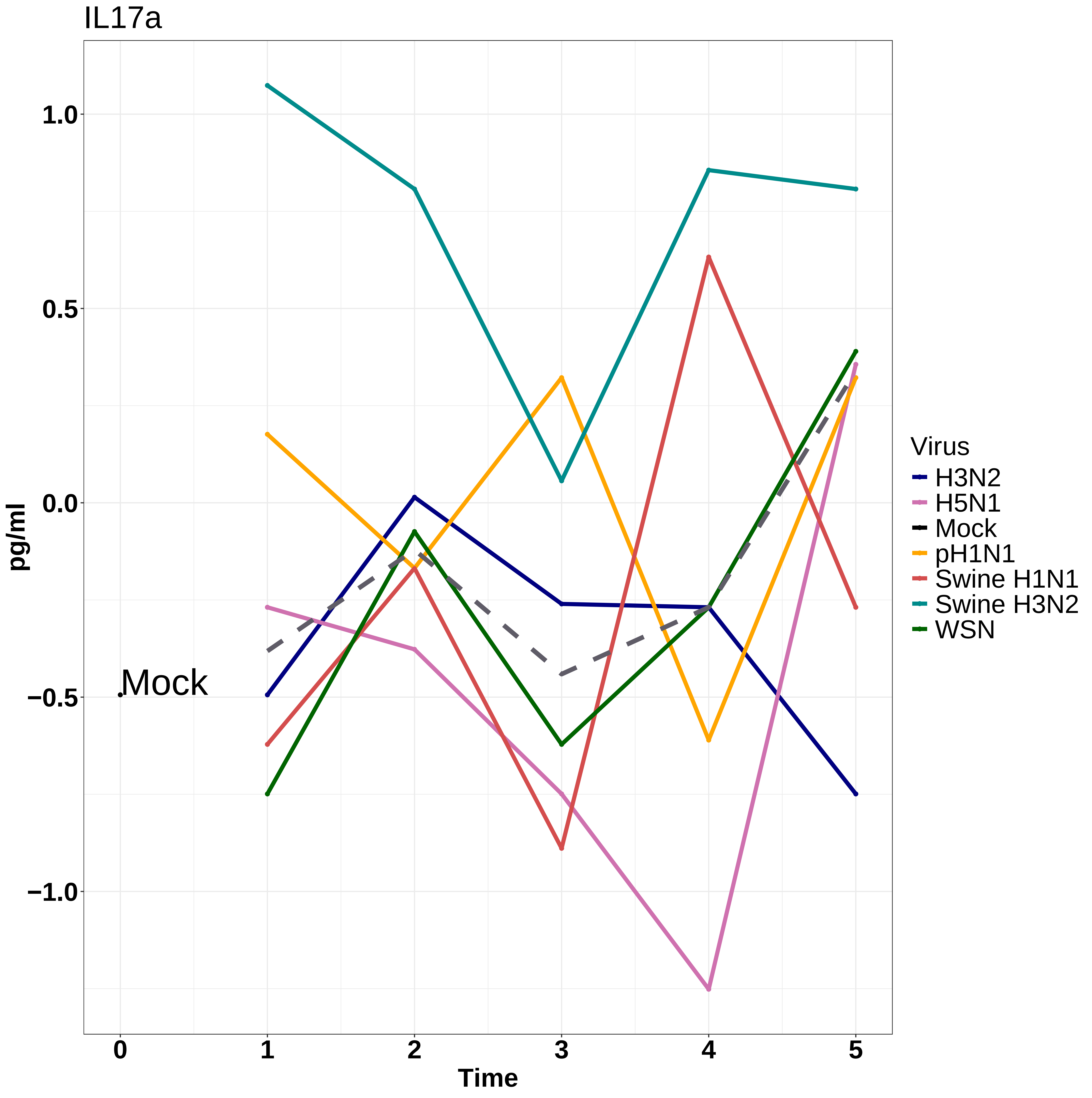

IL17a
pg/ml
1.0
0.5
0.0
−0.5
−1.0
Mock
0
1
2
3
4
5
Time
Virus
H3N2
H5N1
Mock
pH1N1
Swine H1N1
Swine H3N2
WSN

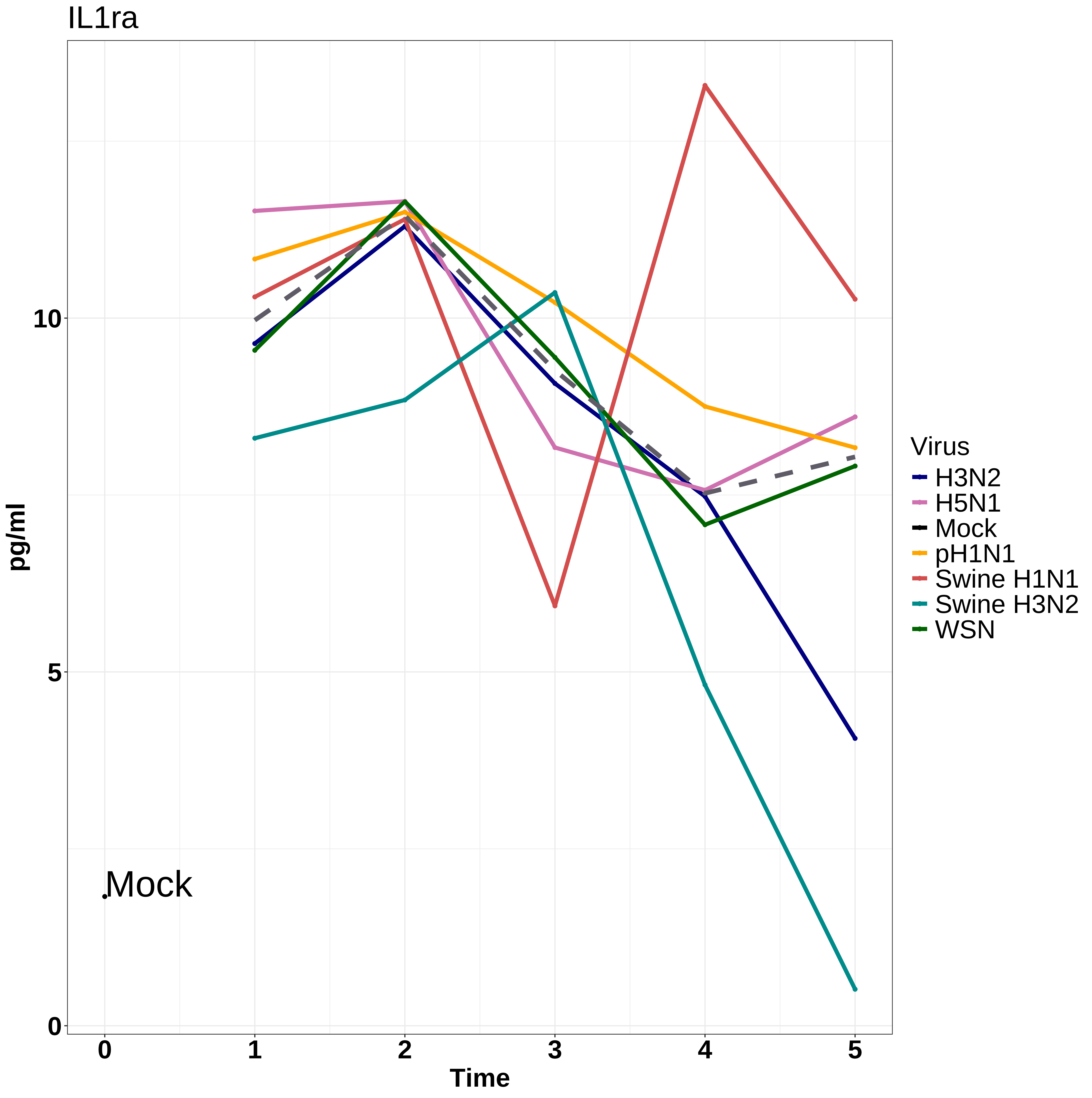

IL1ra
pg/ml
10
5
0
Time
0
1
2
3
4
5
Mock
Virus
H3N2
H5N1
Mock
pH1N1
Swine H1N1
Swine H3N2
WSN

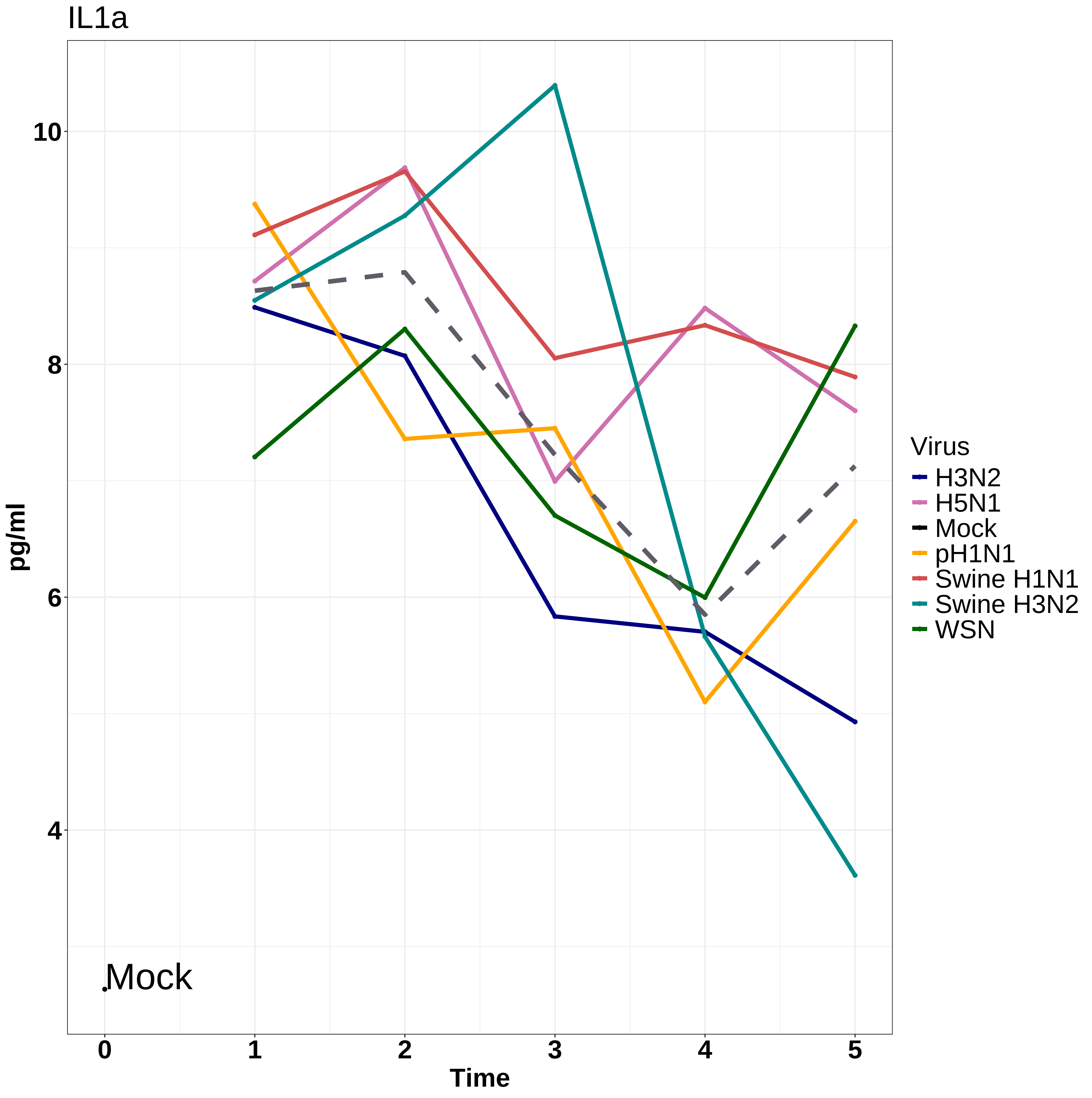
IL1a
10
8
6
4
pg/ml
0
1
2
3
4
5
Time
Mock
Virus
H3N2
H5N1
Mock
pH1N1
Swine H1N1
Swine H3N2
WSN

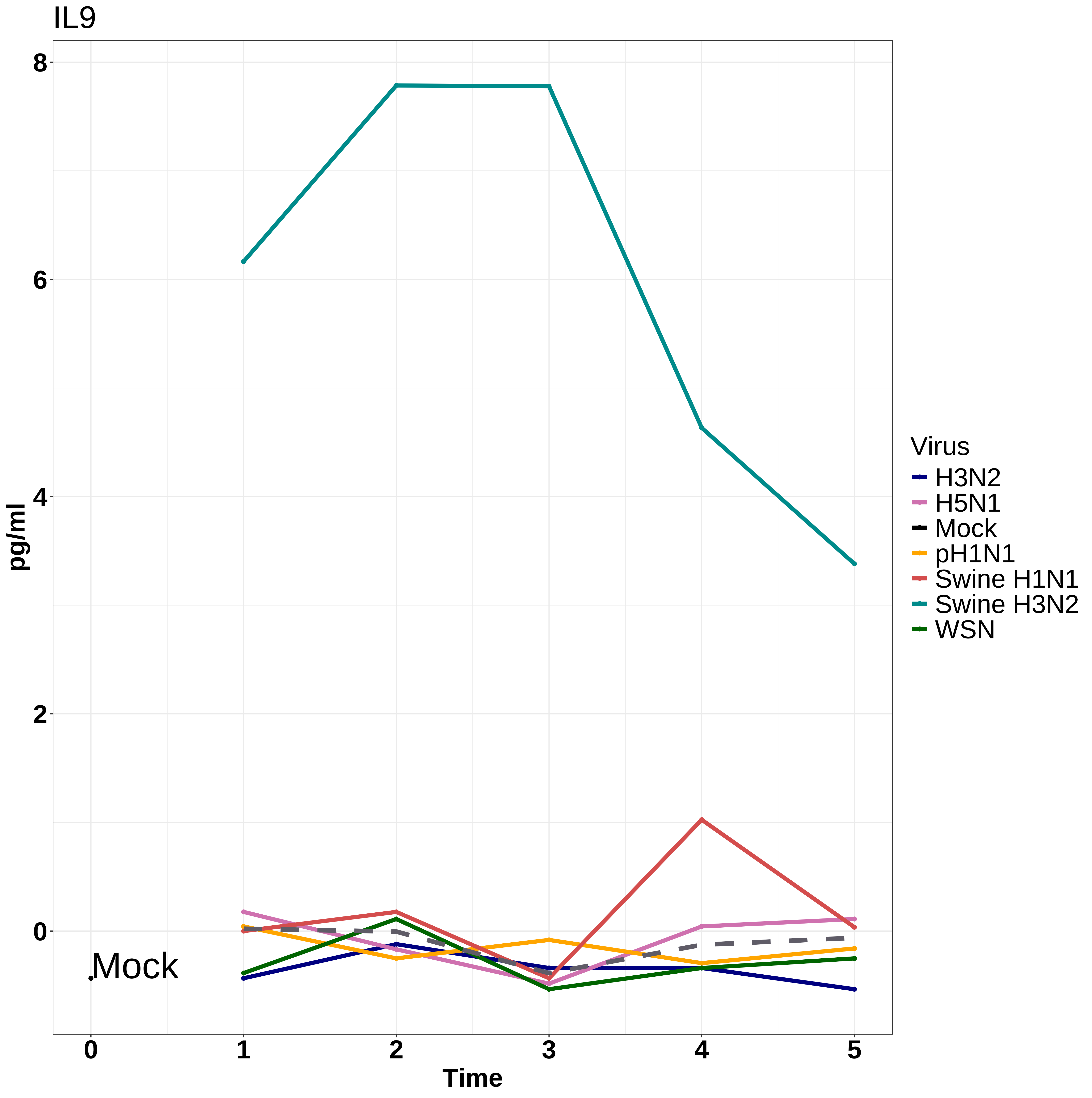
IL9
8
6
4
2
0
pg/ml
Mock
0
1
2
3
4
5
Time
Virus
H3N2
H5N1
Mock
pH1N1
Swine H1N1
Swine H3N2
WSN

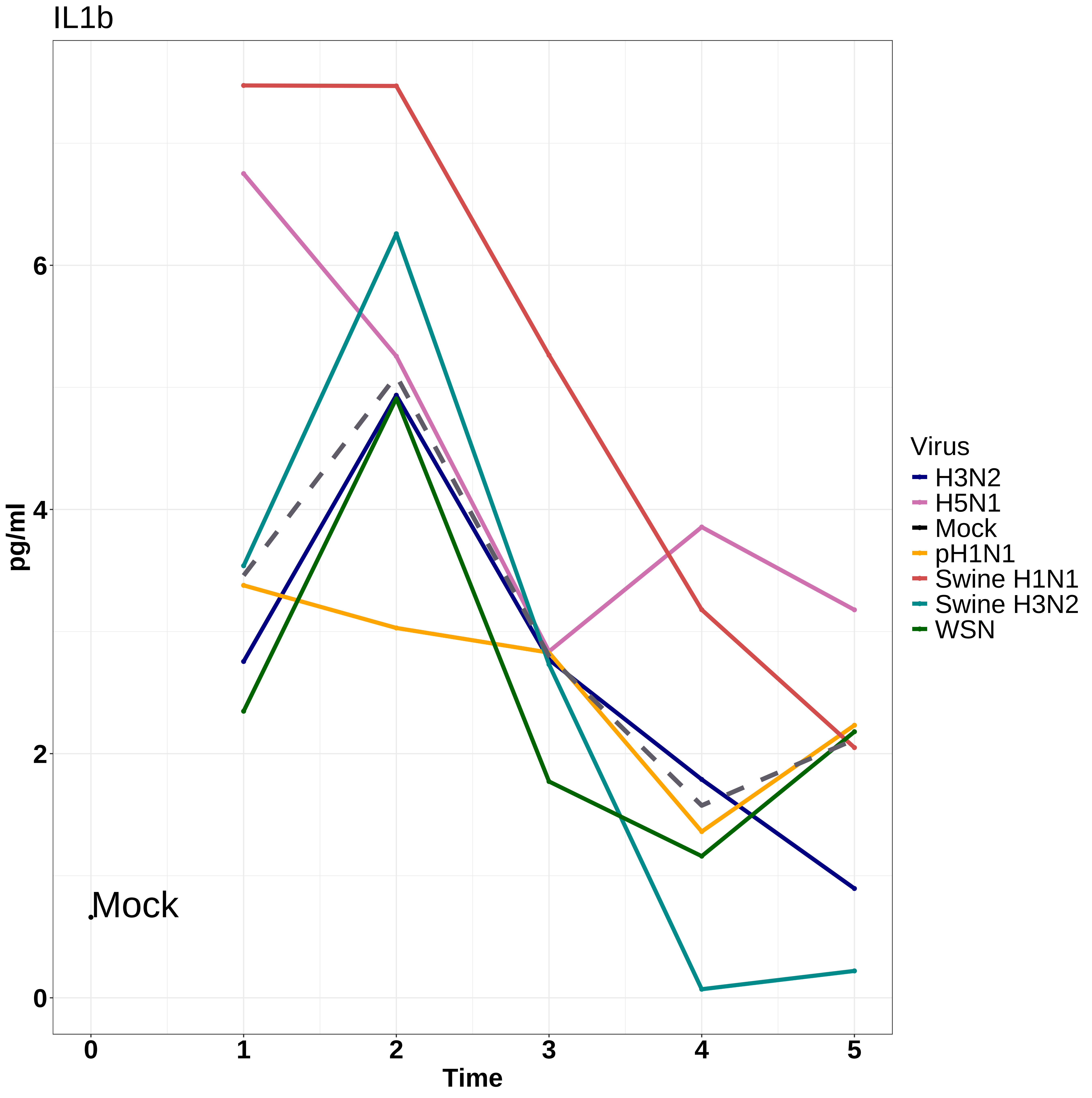
IL1b
pg/ml
Time
0
2
4
6
0
1
2
3
4
5
Mock
Virus
H3N2
H5N1
Mock
pH1N1
Swine H1N1
Swine H3N2
WSN

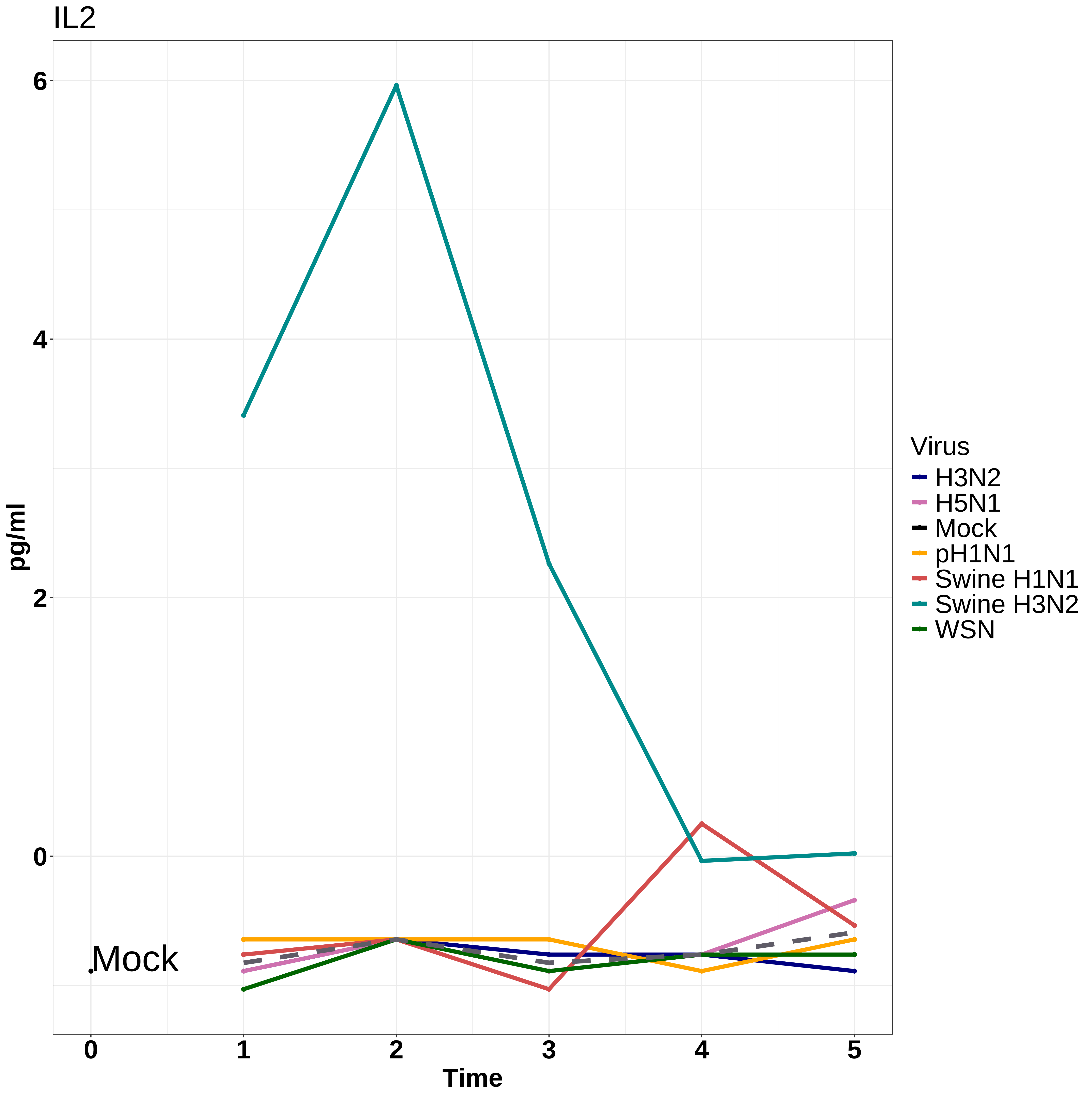

IL2
6
4
2
0
pg/ml
Mock
0
1
2
3
4
5
Time
Virus
H3N2
H5N1
Mock
pH1N1
Swine H1N1
Swine H3N2
WSN

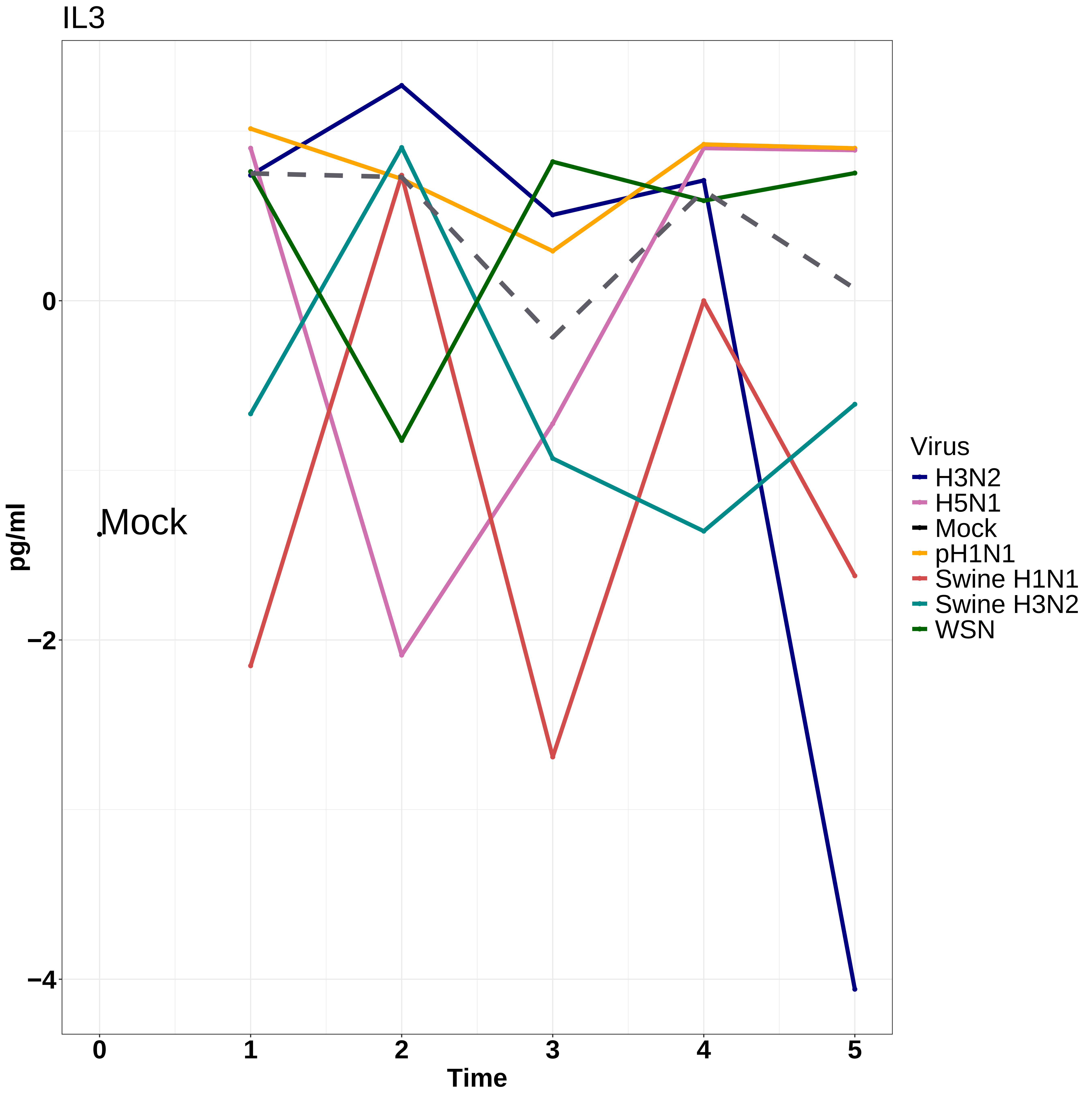
IL3
0
−2
−4
pg/ml
Mock
0
1
2
3
4
5
Time
Virus
H3N2
H5N1
Mock
pH1N1
Swine H1N1
Swine H3N2
WSN

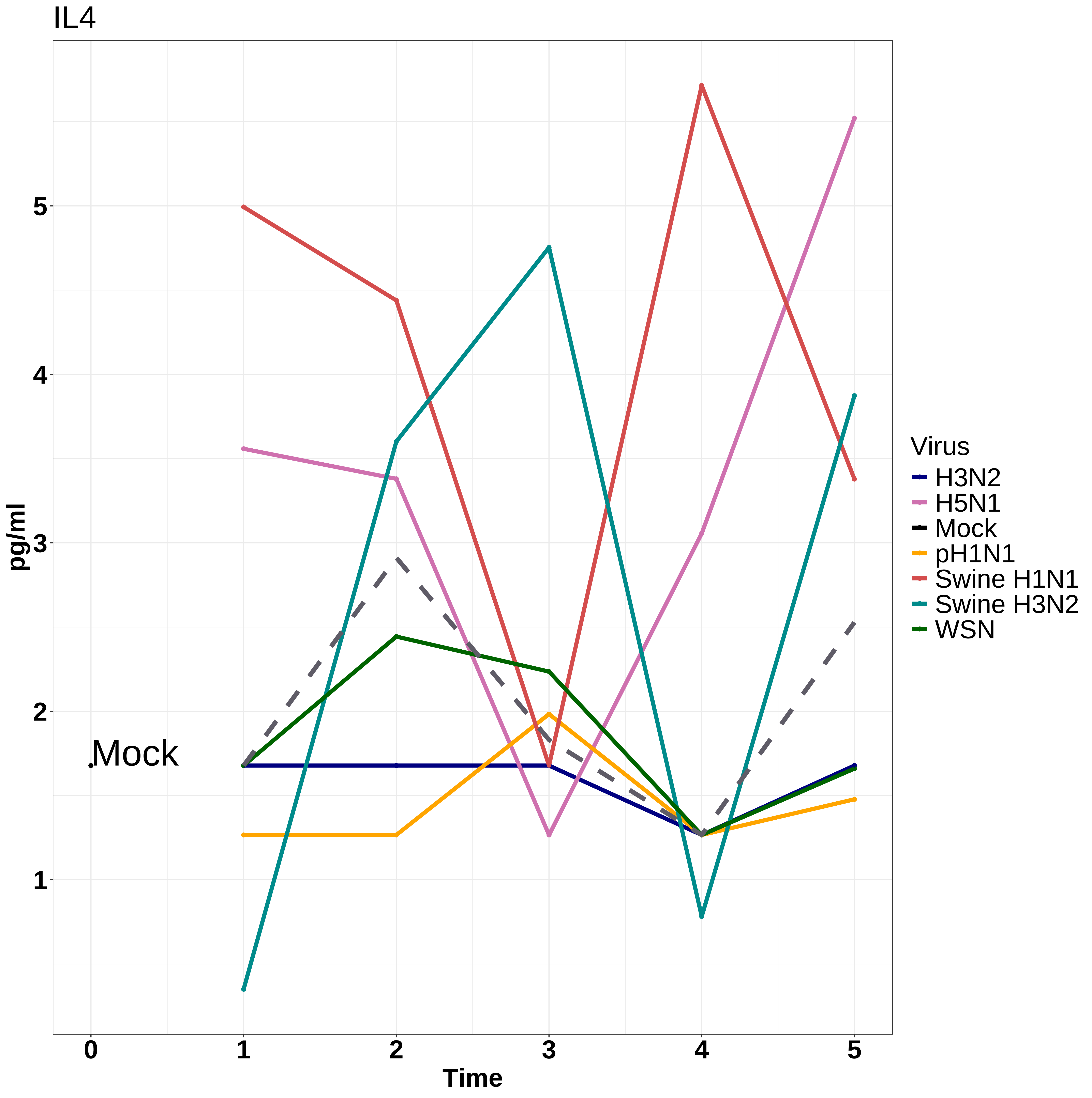

IL4
pg/ml
5
4
3
2
1
Mock
0
1
2
3
4
5
Time
Virus
H3N2
H5N1
Mock
pH1N1
Swine H1N1
Swine H3N2
WSN

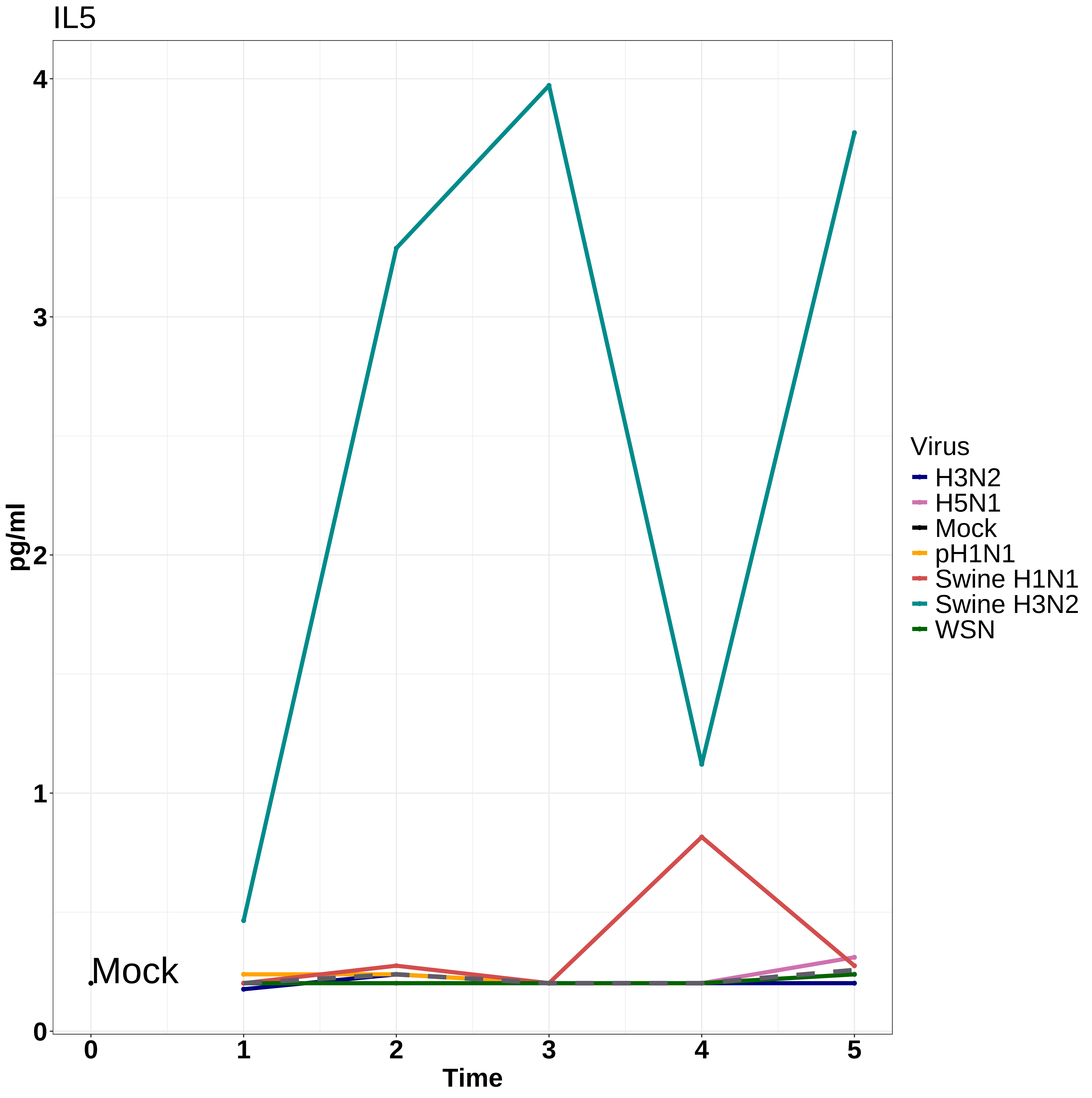
IL5
pg/ml
0
1
2
3
4
Time
0
1
2
3
4
5
Mock
Virus
H3N2
H5N1
Mock
pH1N1
Swine H1N1
Swine H3N2
WSN

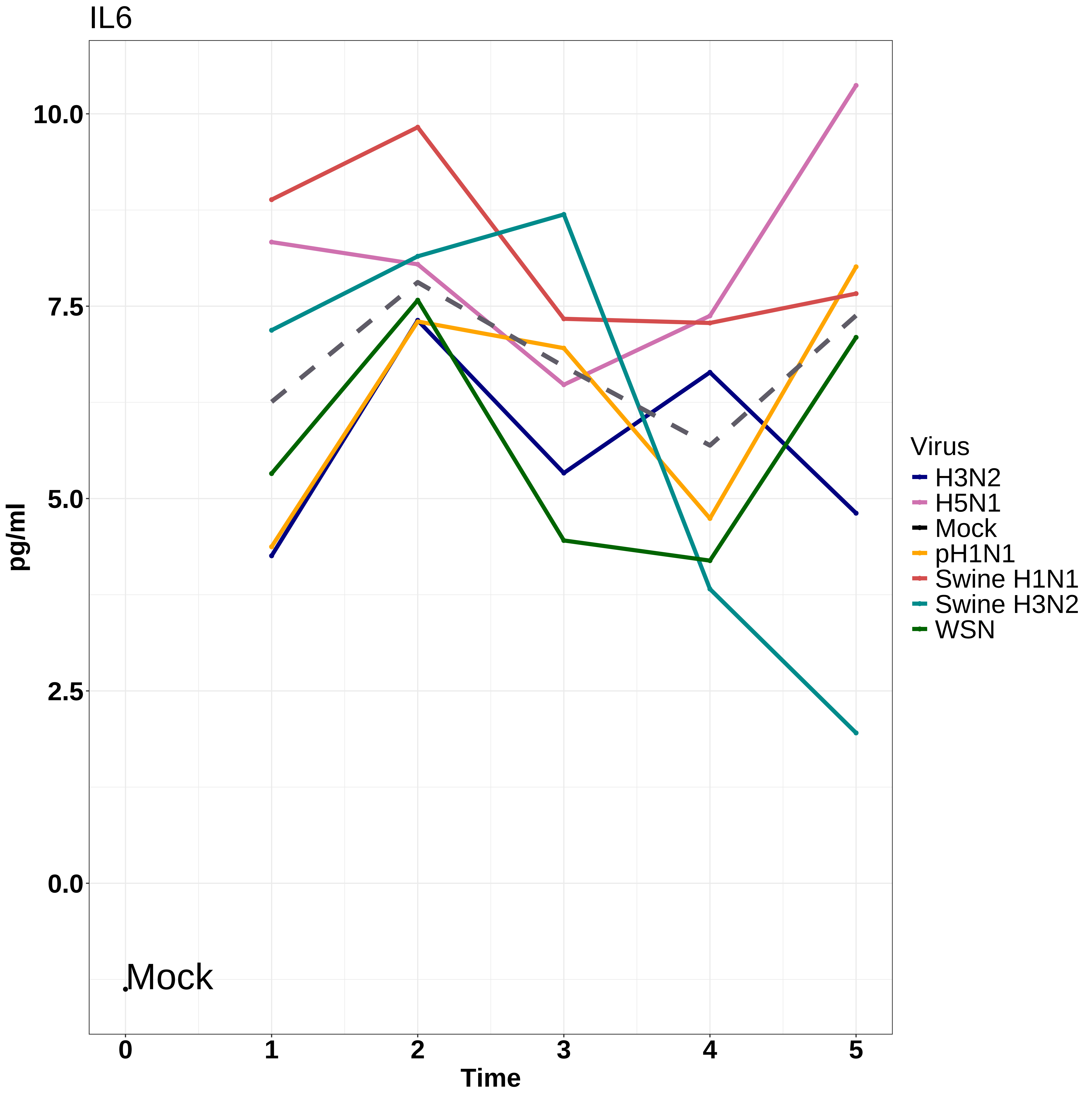

IL6
pg/ml
10.0
7.5
5.0
2.5
0.0
Mock
0
1
2
3
4
5
Time
Virus
H3N2
H5N1
Mock
pH1N1
Swine H1N1
Swine H3N2
WSN

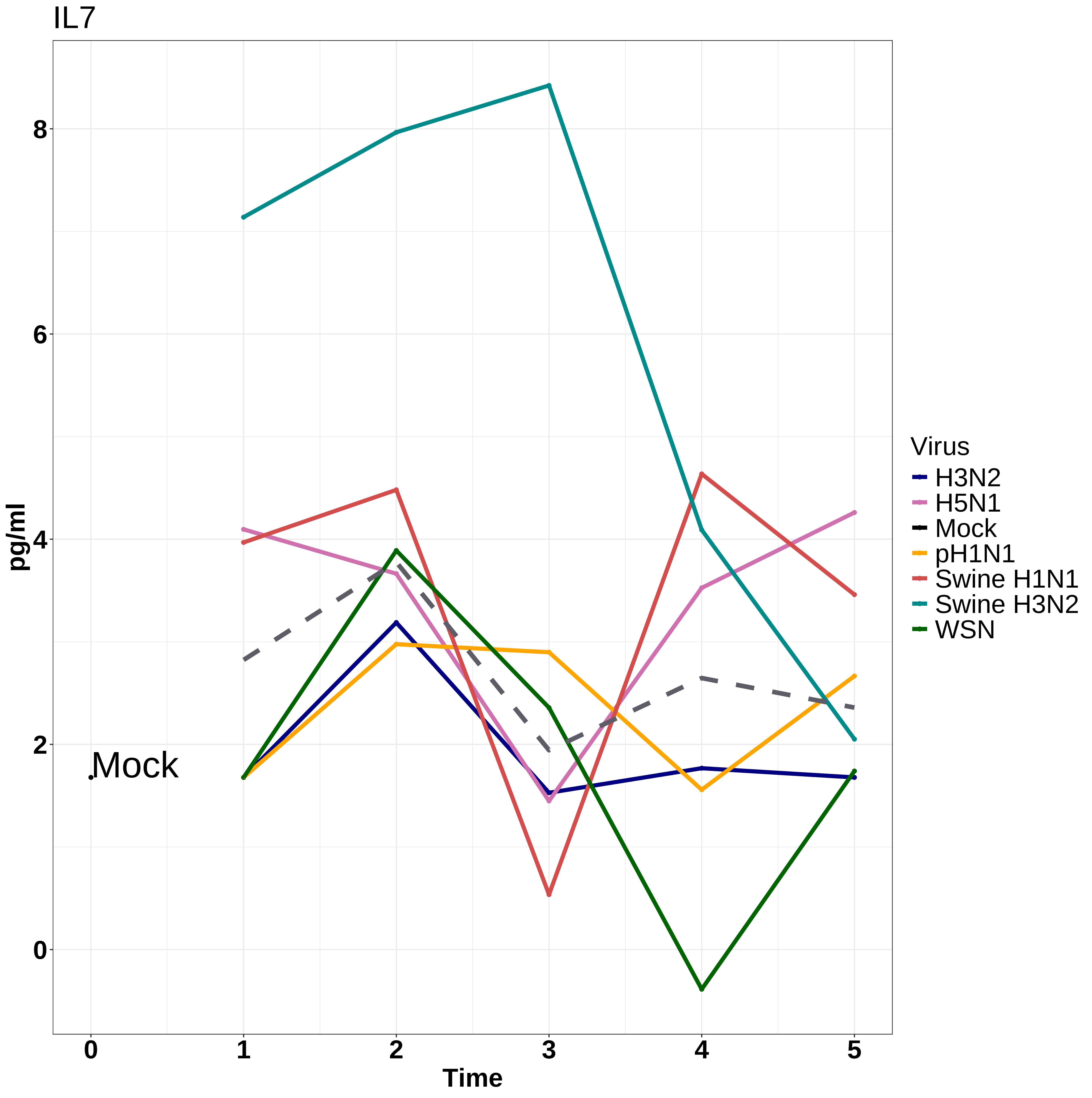

IL7
8
6
4
2
0
pg/ml
Mock
0
1
2
3
4
5
Time
Virus
H3N2
H5N1
Mock
pH1N1
Swine H1N1
Swine H3N2
WSN

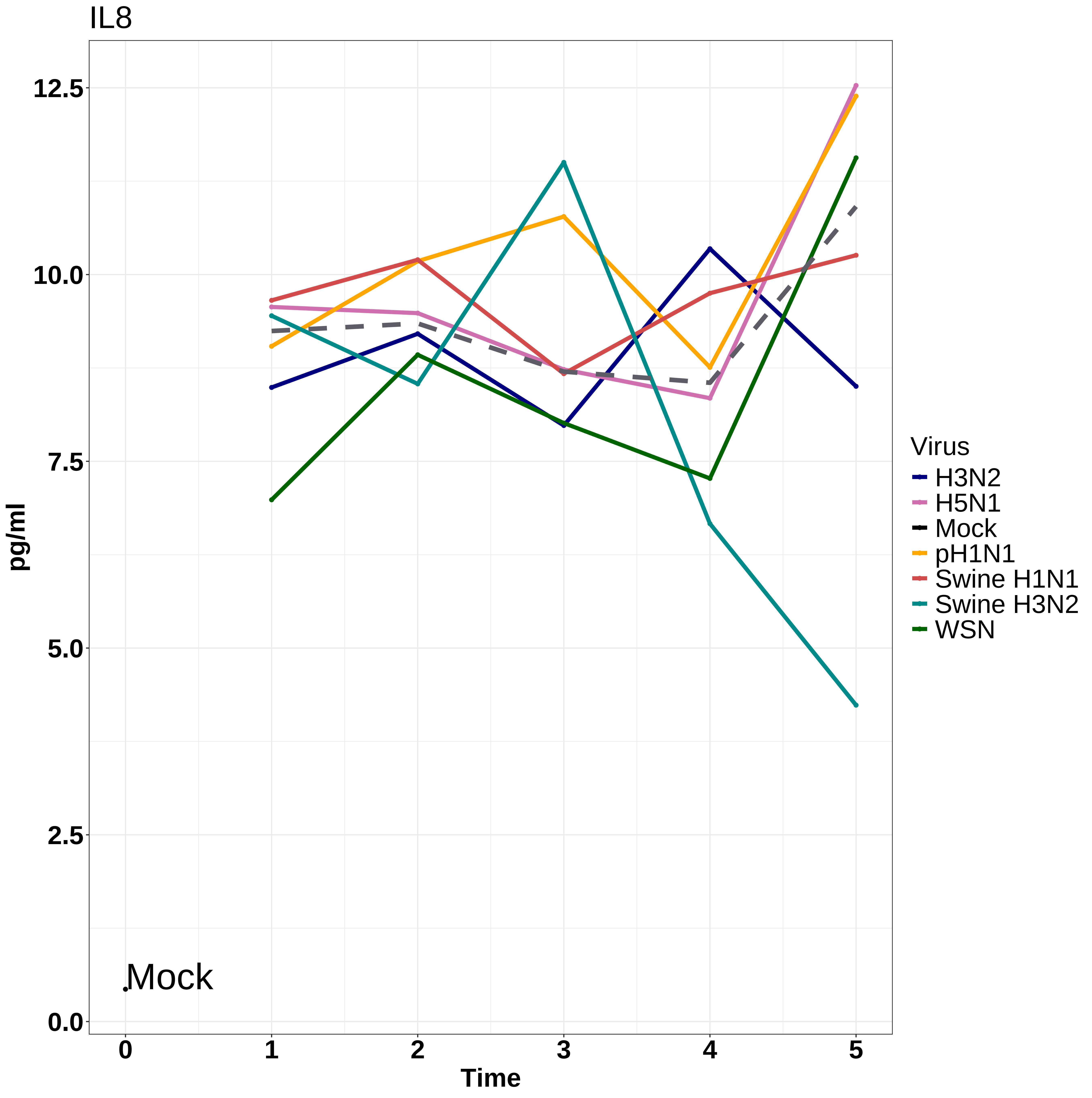

IL8
12.5
10.0
7.5
5.0
2.5
0.0
pg/ml
0
1
2
3
4
5
Time
Mock
Virus
H3N2
H5N1
Mock
pH1N1
Swine H1N1
Swine H3N2
WSN

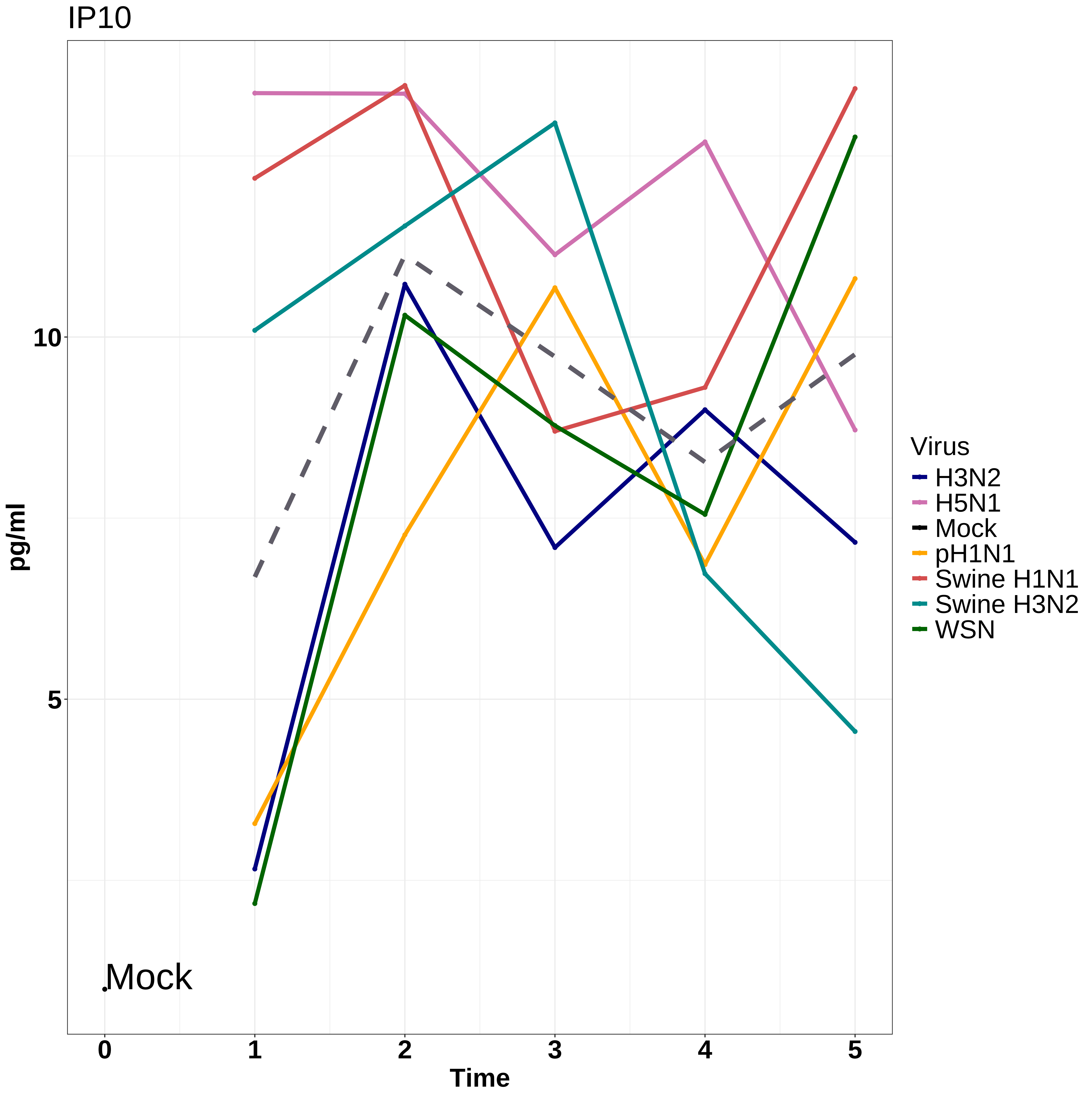
IP10
pg/ml
10
5
Mock
0
1
2
3
4
5
Time
Virus
H3N2
H5N1
Mock
pH1N1
Swine H1N1
Swine H3N2
WSN

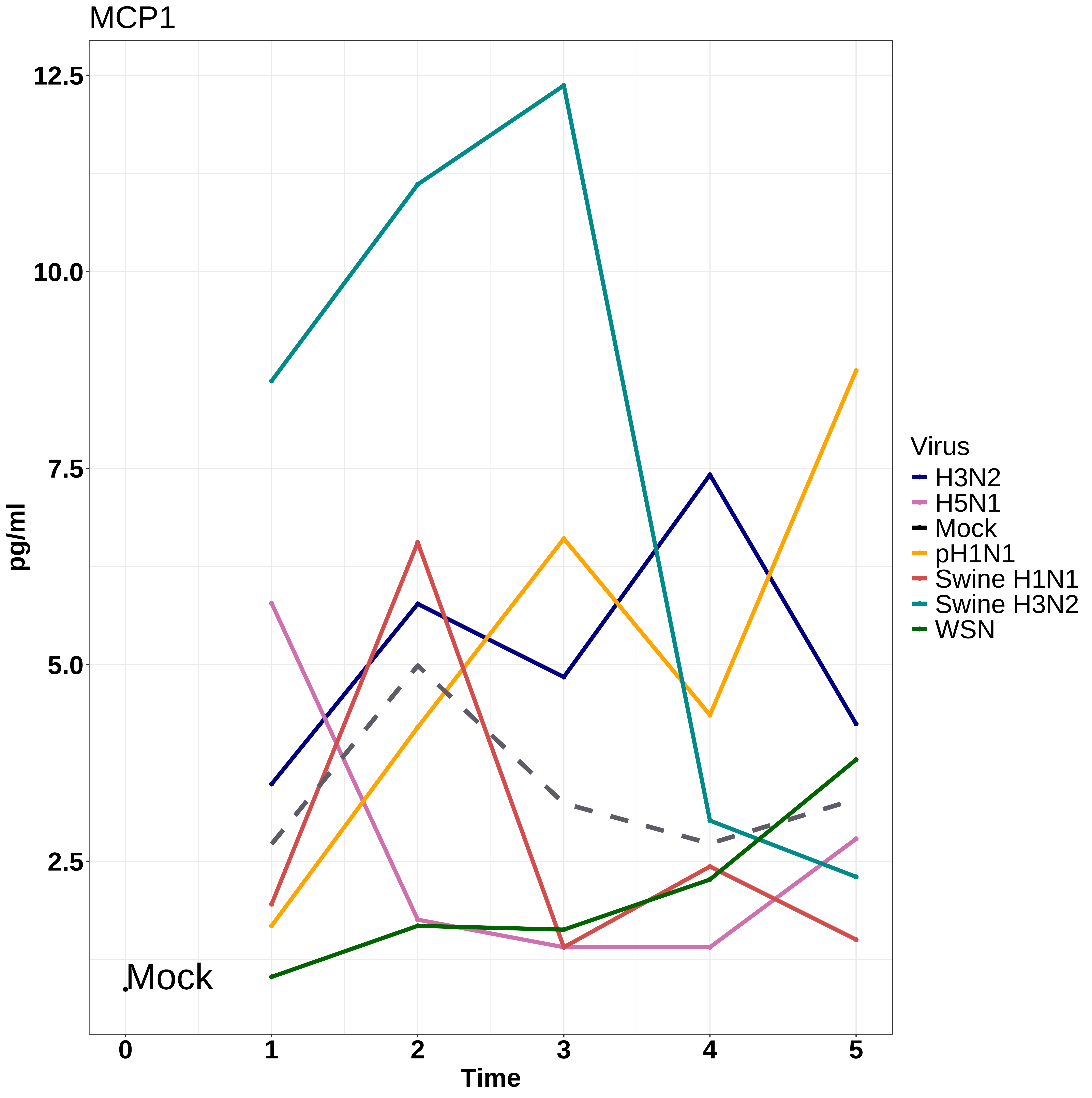
MCP1
pg/ml
12.5
10.0
7.5
5.0
2.5
Mock
0
1
2
3
4
5
Time
Virus
H3N2
H5N1
Mock
pH1N1
Swine H1N1
Swine H3N2
WSN

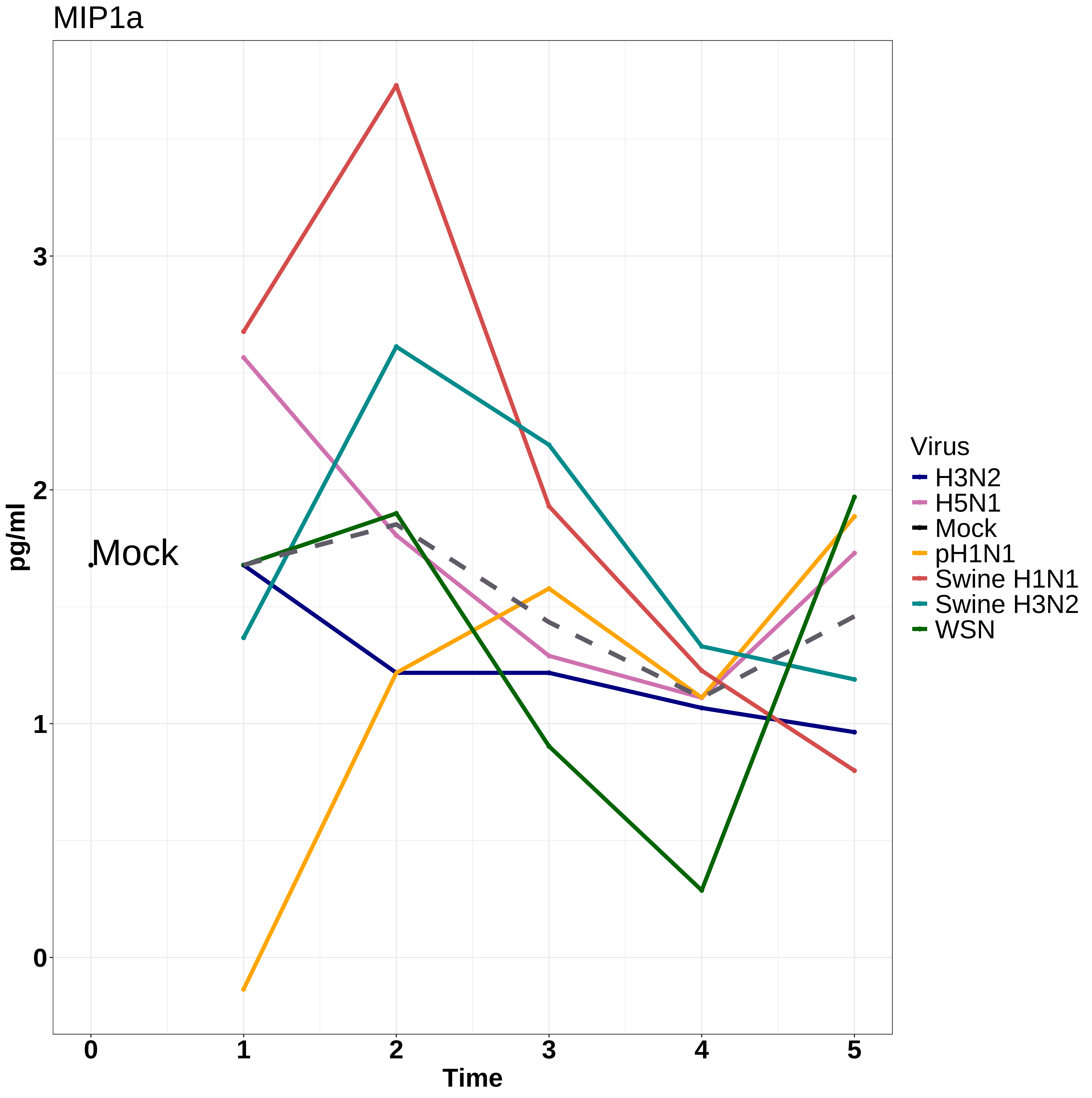

MIP1a
pg/ml
3
2
1
0
Mock
Time
0
1
2
3
4
5
Virus
H3N2
H5N1
Mock
pH1N1
Swine H1N1
Swine H3N2
WSN

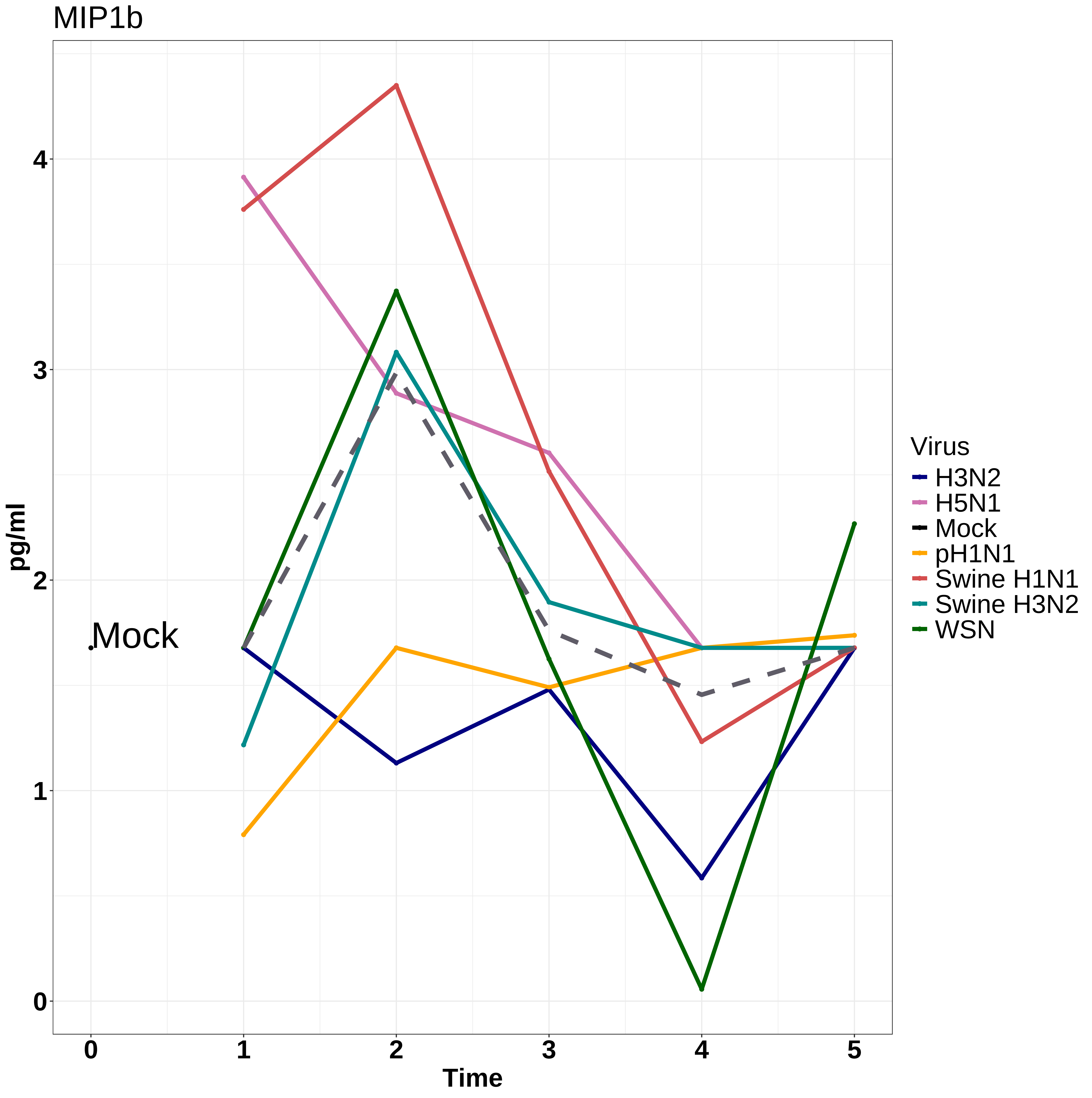

MIP1b
pg/ml
0
1
2
3
4
Time
0
1
2
3
4
5
Mock
Virus
H3N2
H5N1
Mock
pH1N1
Swine H1N1
Swine H3N2
WSN

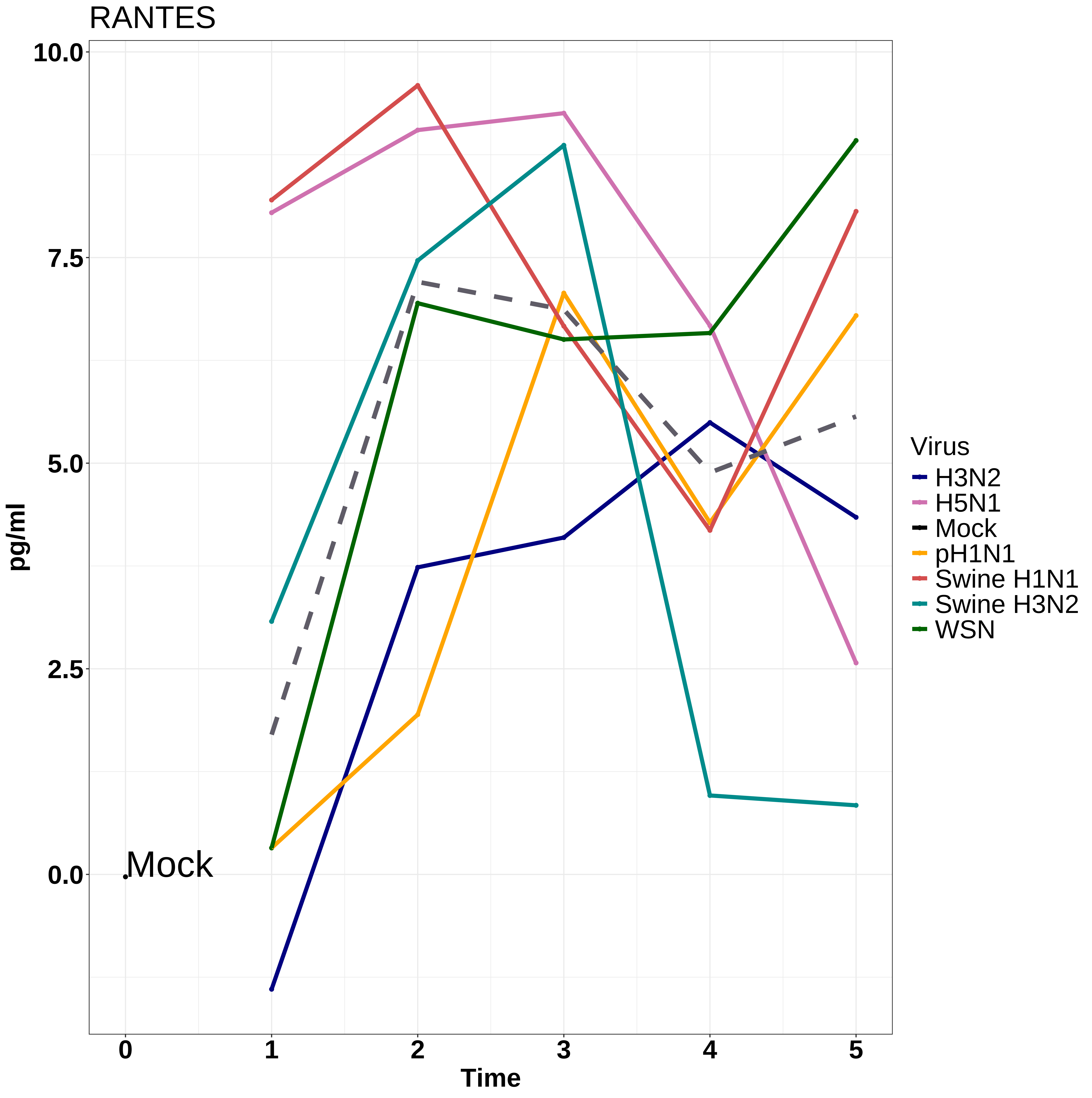
RANTES
10.0
7.5
5.0
2.5
0.0
pg/ml
Mock
0
1
2
3
4
5
Time
Virus
H3N2
H5N1
Mock
pH1N1
Swine H1N1
Swine H3N2
WSN

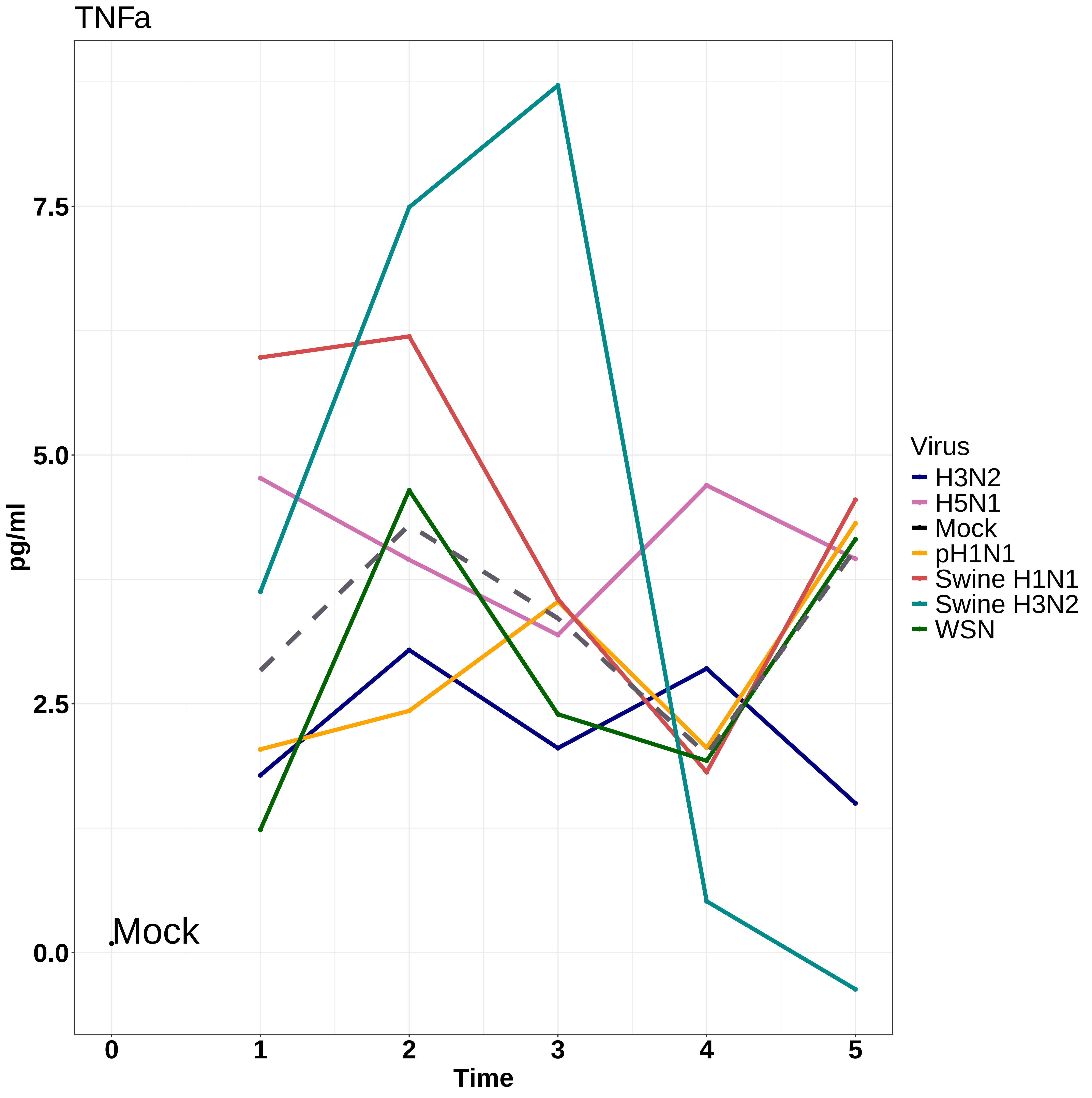
TNFa
7.5
5.0
2.5
0.0
0
1
2
3
4
5
Time
pg/ml
Mock
Virus
H3N2
H5N1
Mock
pH1N1
Swine H1N1
Swine H3N2
WSN

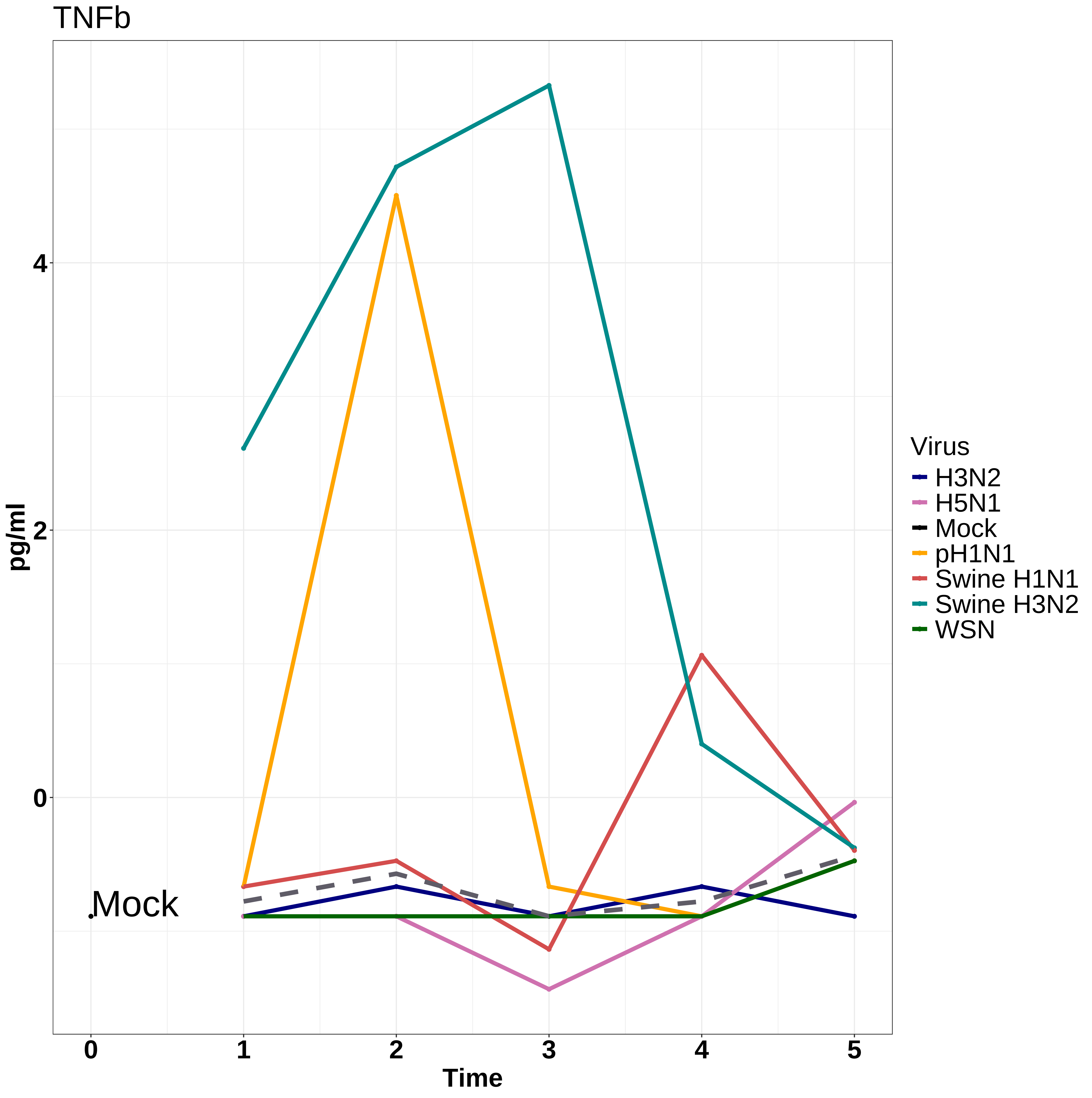
TNFb
pg/ml
4
2
0
Time
0
1
2
3
4
5
Mock
Virus
H3N2
H5N1
Mock
pH1N1
Swine H1N1
Swine H3N2
WSN

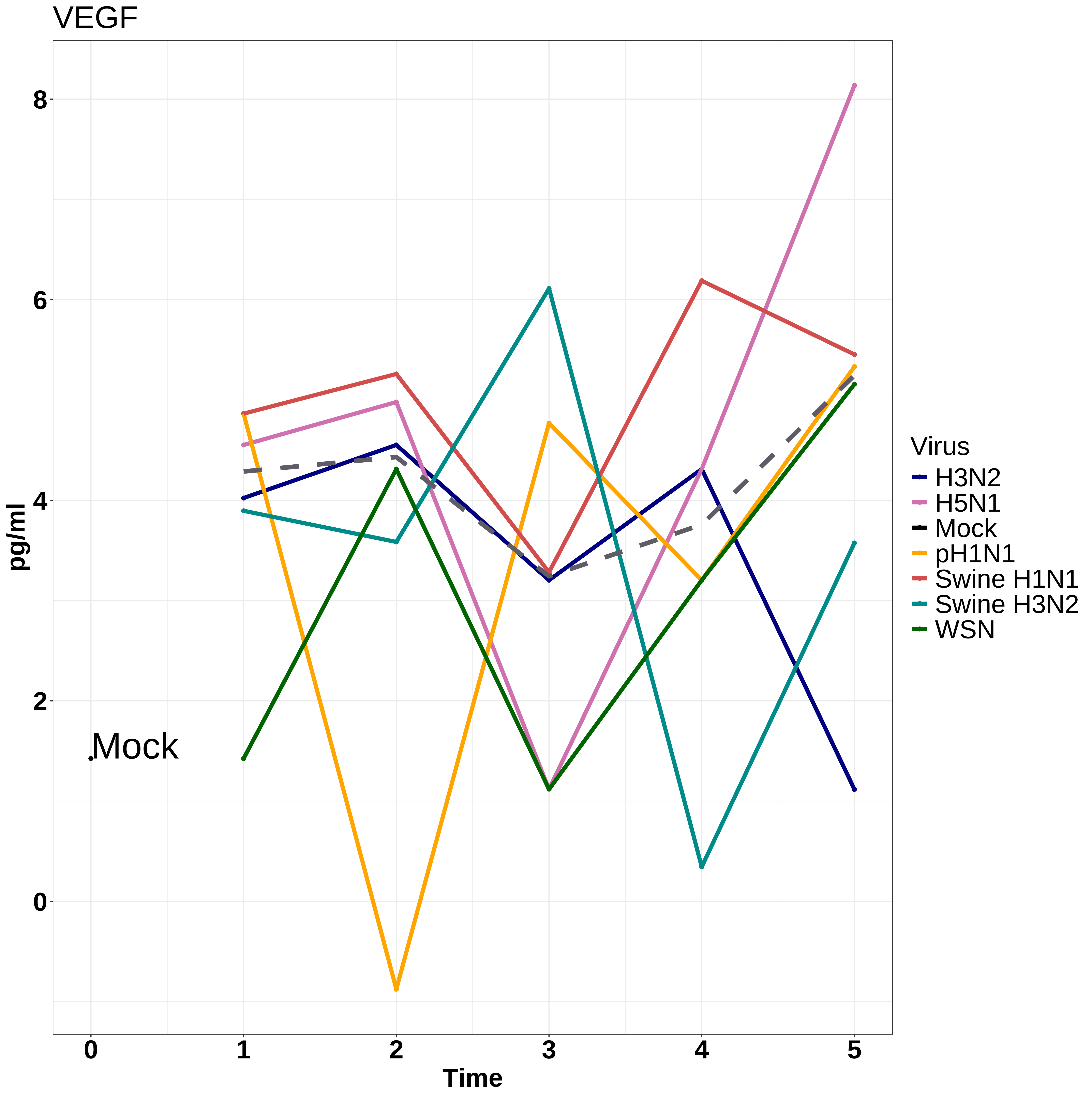
VEGF
pg/ml
8
6
4
2
0
Time
0
1
2
3
4
5
Mock
Virus
H3N2
H5N1
Mock
pH1N1
Swine H1N1
Swine H3N2
WSN
