# Supplementary material for: Modulation of cytokeratin and cytokine/chemokine expression following influenza virus infection of differentiated human tonsillar epithelial cells
Source: J Virol. 2025 Jan 10;99(2):e01460-24. doi: 10.1128/jvi.01460-24 (PMC11852761; doi:10.1128/jvi.01460-24)
Supplement: Supplemental figures — Figures S1 to S6 and legends for all supplemental material. [file jvi.01460-24-s0002.docx]

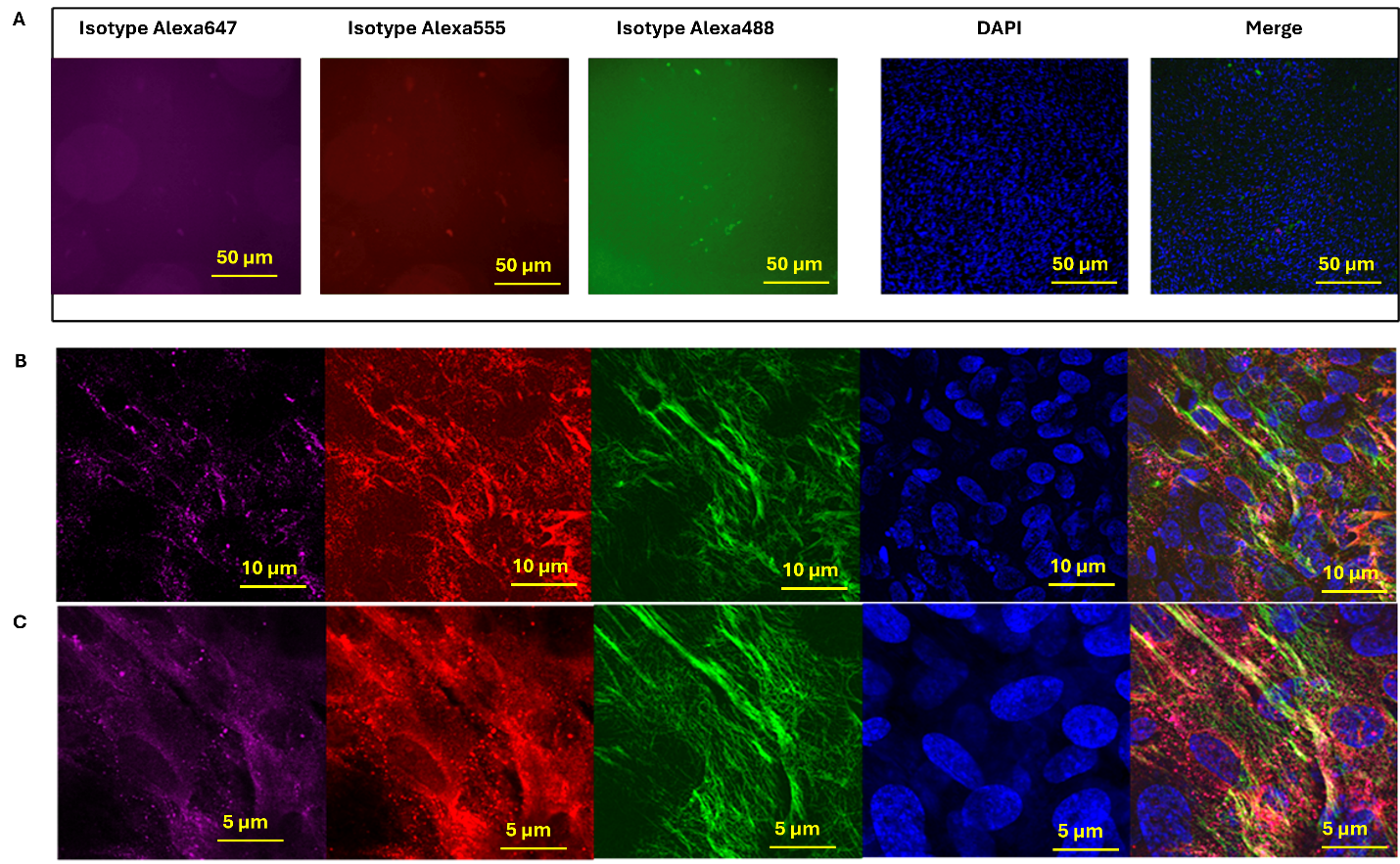


**Supplemental Figure S1.** Supplemental immunofluorescent confocal microscopy images. (A) Isotype control images for each fluorophore. (B) 63x magnification image as for Figure 1C, showing an additional field of cells. (C) 68x view of cells shown in (B).


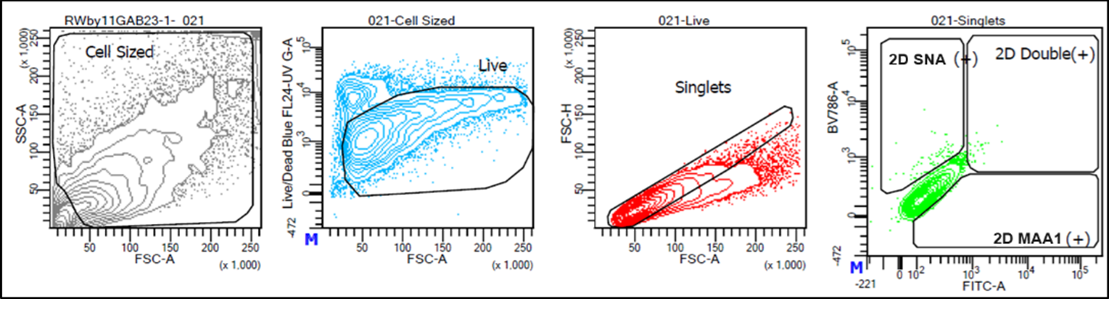


**Supplemental Figure S2.** Gating controls for Sialic acid distribution in HTECs. Cell populations were gated to exclude cellular debris based on overall size and intracellular complexity (first panel), then include only live cells via the absence of live-dead stain (second panel), then include only single cells (third panel). The gates determining cells positive for MAA I-FITC lectin (avian α2,3-linked SA receptors) and SNA-BV786 lectin (α2,6-linked SA receptors) are based on unstained controls for those fluorophores (fourth panel).


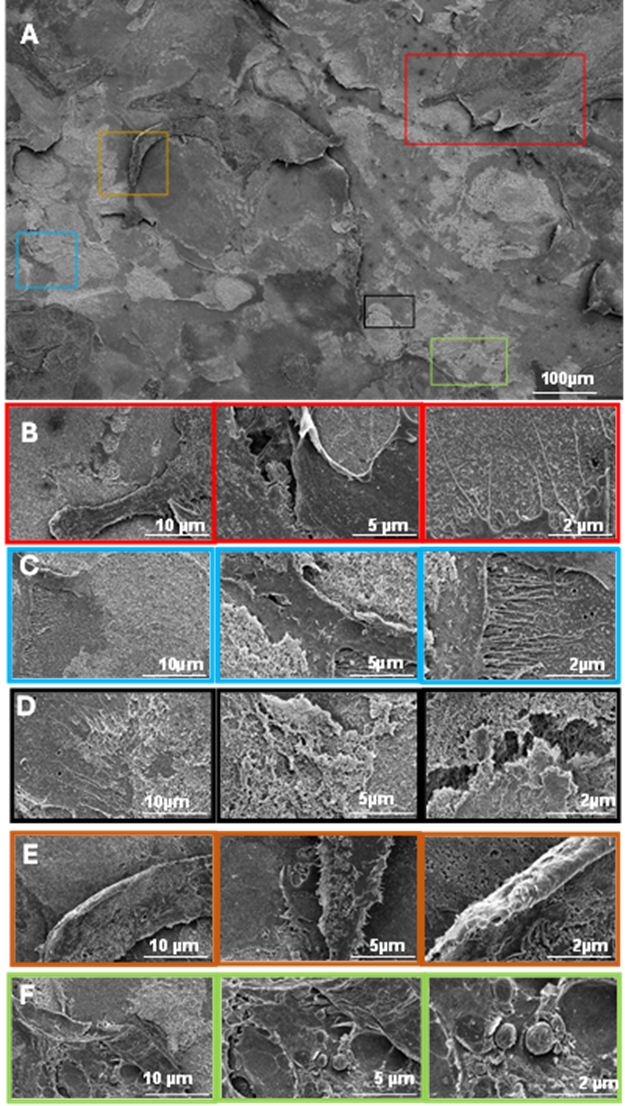


**Supplemental Figure S3.** Scanning electron microscopy views of HTECs infected with A/TN/1-560/2009 (pH1N1) at day 5 post-infection. (A) The apical surface of the infected HTECs, displaying intact infected cell layers and infected cells with peeled membranes, elongated cilia, and disrupted membranes. Panels B-F come from fields in panel A as denoted with matching frame colors. (B, C) cells with exposed actin cytoskeletons (panels with 10μm scale bars), peeled cell membranes (panels with 5μm scale bars), and filopodia filaments formed by the virus connecting adjacent infected cells (panels with 2μm scale bars). (D) Disrupted cell membrane with condensed cell debris, profusion of vacuoles, and scraped vacuoles with exposed content. (E) Elongated, collapsed, and ciliated infected cells. (F) Necrotic micro-crypts with the appearance of transport vehicles.


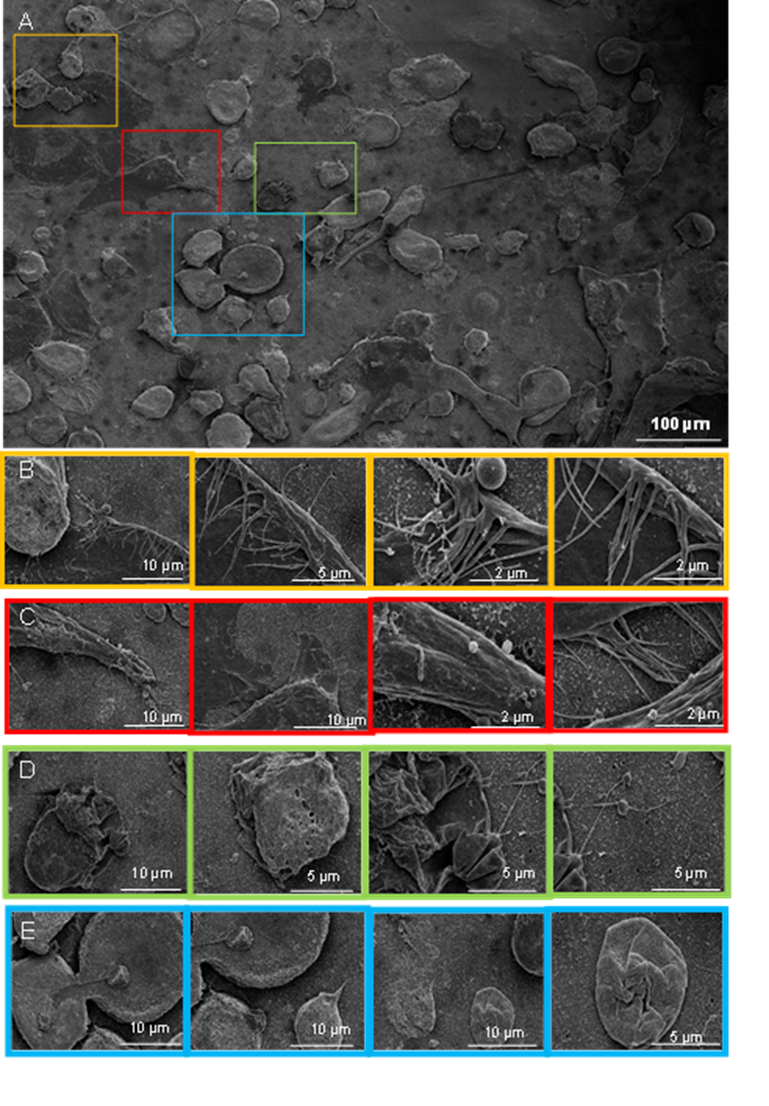


**Supplemental Figure S4.** Scanning electron microscopy views of HTECs infected with A/Swine/OH/15TOSU4783/2016 (H3N2) at day 5 post infection. (A) The apical surface of the infected HTECs, displaying disrupted and collapsed cells (scale bar: 100μm). Panels B-E come from fields in panel A as denoted with matching frame colors. Scales are indicated in each frame. (B, C) Filopodia and filaments connecting infected cells. (D, E) Collapsed cells. Note the connection between the collapsed cells in E.


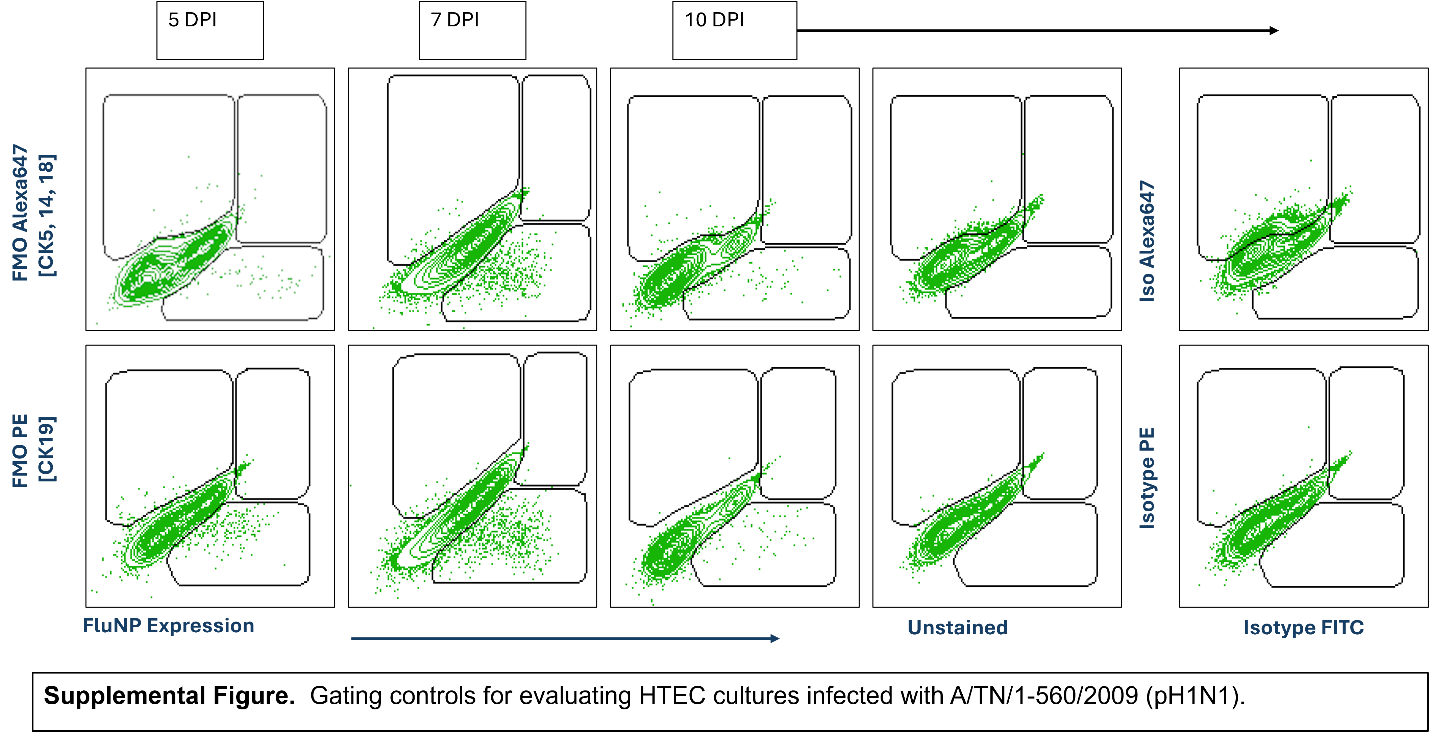


**Supplemental Figure S5.** Gating controls for evaluating HTEC cultures infected with A/TN/1-560/2009 (pH1N1), as shown in figure 5.


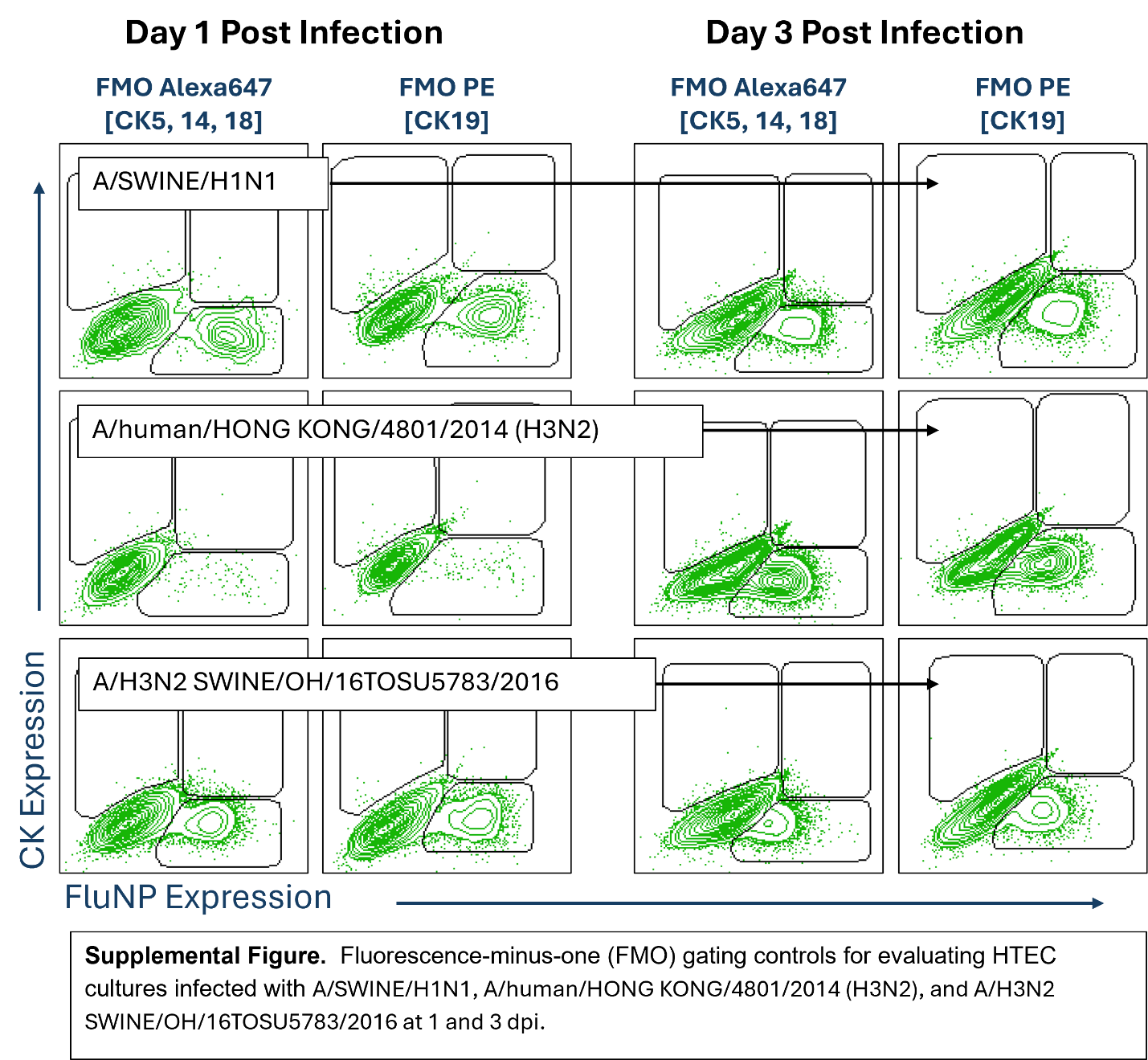


**Supplemental Figure S6**. Fluorescence-minus-one (FMO) gating controls for evaluating HTEC cultures infected with A/SWINE/H1N1, A/human/HONG KONG/4801/2014 (H3N2), and A/H3N2 SWINE/OH/16TOSU5783/2016 at 1 and 3 dpi, as shown in figure 6.

**Legends for other supplemental material**

**File S1.** Longitudinal cytokine values based on influenza virus strain infection.

**Table S1.** Linear regression on log2 fold changes for each cytokine, with virus and time as independent variables. Bold highlights events where p < 0.05.

**Video S1.** 3D images stack related to Figure 1C. Images shown from top to bottom stack with a step size of 412 nm.

**Video S2.** Higher resolution 3D images stack related to figure 1C. Images shown from bottom to top stack with a step size of 412 nm.
