# Supplementary material for: Modulation of cytokeratin and cytokine/chemokine expression following influenza virus infection of differentiated human tonsillar epithelial cells
Source: J Virol. 2025 Jan 10;99(2):e01460-24. doi: 10.1128/jvi.01460-24 (PMC11852761; doi:10.1128/jvi.01460-24)
Supplement: Table S1 — Linear regression on log2 fold changes for each cytokine, with virus and time as independent variables. [file jvi.01460-24-s0003.docx]

**Table S1.** Linear regression on log_2_ fold changes for each cytokine, with virus and time as independent variables. Bold highlights events where *p* < 0.05.

| **Row Labels** | **Estimate** | ***P*** |
| --- | --- | --- |
| **EGF** |  |  |
| (Intercept) | 1.239 | 0.496 |
| Time | 0.093 | 0.691 |
| VirusH3N2 | 1.070 | 0.612 |
| VirusH5N1 | 2.970 | 0.167 |
| ViruspH1N1 | 0.694 | 0.742 |
| VirusSwine H1N1 | 3.140 | 0.145 |
| VirusSwine H3N2 | 3.844 | 0.078 |
| VirusWSN | 1.027 | 0.626 |
| **Eotaxin** |  |  |
| (Intercept) | 0.465 | 0.515 |
| Time | 0.143 | 0.128 |
| VirusH3N2 | 0.371 | 0.653 |
| VirusH5N1 | 0.927 | 0.268 |
| ViruspH1N1 | 0.930 | 0.266 |
| VirusSwine H1N1 | 1.245 | 0.141 |
| VirusSwine H3N2 | 5.483 | **0.000** |
| VirusWSN | 0.498 | 0.548 |
| **FGF2** |  |  |
| (Intercept) | 1.678 | 0.290 |
| Time | 0.004 | 0.985 |
| VirusH3N2 | 3.150 | 0.093 |
| VirusH5N1 | 4.747 | **0.015** |
| ViruspH1N1 | 3.493 | 0.065 |
| VirusSwine H1N1 | 4.644 | **0.017** |
| VirusSwine H3N2 | 5.264 | **0.008** |
| VirusWSN | 3.342 | 0.076 |
| **Flt3L** |  |  |
| (Intercept) | 1.678 | 0.053 |
| Time | -0.133 | 0.222 |
| VirusH3N2 | 0.400 | 0.679 |
| VirusH5N1 | 0.396 | 0.683 |
| ViruspH1N1 | 0.362 | 0.708 |
| VirusSwine H1N1 | 0.527 | 0.586 |
| VirusSwine H3N2 | 3.867 | **0.000** |
| VirusWSN | 0.400 | 0.679 |
| **Fractalkine** |  |  |
| (Intercept) | 1.678 | 0.471 |
| Time | -0.098 | 0.744 |
| VirusH3N2 | 2.016 | 0.456 |
| VirusH5N1 | 4.368 | 0.114 |
| ViruspH1N1 | 2.474 | 0.362 |
| VirusSwine H1N1 | 4.595 | 0.097 |
| VirusSwine H3N2 | 3.033 | 0.266 |
| VirusWSN | 1.844 | 0.495 |
| **GCSF** |  |  |
| (Intercept) | 1.678 | 0.327 |
| Time | -0.063 | 0.773 |
| VirusH3N2 | 4.157 | **0.044** |
| VirusH5N1 | 5.878 | **0.006** |
| ViruspH1N1 | 5.146 | **0.014** |
| VirusSwine H1N1 | 5.073 | **0.016** |
| VirusSwine H3N2 | 3.915 | 0.056 |
| VirusWSN | 3.076 | 0.128 |
| **GMCSF** |  |  |
| (Intercept) | -0.340 | 0.762 |
| Time | 0.399 | **0.011** |
| VirusH3N2 | 2.919 | **0.033** |
| VirusH5N1 | 2.655 | 0.051 |
| ViruspH1N1 | 3.846 | **0.007** |
| VirusSwine H1N1 | 2.322 | 0.085 |
| VirusSwine H3N2 | 1.826 | 0.170 |
| VirusWSN | 2.562 | 0.059 |
| **GRO** |  |  |
| (Intercept) | 1.678 | 0.434 |
| Time | 0.512 | 0.073 |
| VirusH3N2 | 3.925 | 0.122 |
| VirusH5N1 | 6.178 | **0.019** |
| ViruspH1N1 | 5.346 | **0.039** |
| VirusSwine H1N1 | 5.867 | **0.025** |
| VirusSwine H3N2 | 4.671 | 0.069 |
| VirusWSN | 4.533 | 0.077 |
| **IFNa2** |  |  |
| (Intercept) | 1.678 | 0.220 |
| Time | -0.210 | 0.234 |
| VirusH3N2 | 1.506 | 0.340 |
| VirusH5N1 | 2.924 | 0.071 |
| ViruspH1N1 | 2.936 | 0.070 |
| VirusSwine H1N1 | 2.814 | 0.082 |
| VirusSwine H3N2 | 3.447 | **0.036** |
| VirusWSN | 1.922 | 0.226 |
| **IFNg** |  |  |
| (Intercept) | -0.160 | 0.823 |
| Time | -0.049 | 0.594 |
| VirusH3N2 | 0.599 | 0.474 |
| VirusH5N1 | 1.405 | 0.101 |
| ViruspH1N1 | 1.087 | 0.200 |
| VirusSwine H1N1 | 1.701 | 0.050 |
| VirusSwine H3N2 | 3.085 | **0.001** |
| VirusWSN | 0.815 | 0.333 |
| **IL10** |  |  |
| (Intercept) | 0.131 | 0.892 |
| Time | -0.095 | 0.451 |
| VirusH3N2 | 0.611 | 0.587 |
| VirusH5N1 | 1.169 | 0.303 |
| ViruspH1N1 | 0.965 | 0.393 |
| VirusSwine H1N1 | 1.428 | 0.211 |
| VirusSwine H3N2 | 7.827 | **0.000** |
| VirusWSN | 0.635 | 0.573 |
| **IL12p40** |  |  |
| (Intercept) | 1.678 | **0.001** |
| Time | -0.007 | 0.902 |
| VirusH3N2 | -0.149 | 0.783 |
| VirusH5N1 | -0.618 | 0.259 |
| ViruspH1N1 | 0.077 | 0.887 |
| VirusSwine H1N1 | 0.170 | 0.753 |
| VirusSwine H3N2 | 0.248 | 0.647 |
| VirusWSN | -0.298 | 0.582 |
| **IL12p70** |  |  |
| (Intercept) | -1.690 | 0.154 |
| Time | -0.006 | 0.969 |
| VirusH3N2 | 0.618 | 0.647 |
| VirusH5N1 | 1.677 | 0.221 |
| ViruspH1N1 | 1.071 | 0.430 |
| VirusSwine H1N1 | 1.757 | 0.201 |
| VirusSwine H3N2 | 3.804 | **0.009** |
| VirusWSN | 1.155 | 0.395 |
| **IL13** |  |  |
| (Intercept) | 1.678 | 0.327 |
| Time | -0.289 | 0.195 |
| VirusH3N2 | -0.586 | 0.766 |
| VirusH5N1 | -0.242 | 0.902 |
| ViruspH1N1 | -0.994 | 0.615 |
| VirusSwine H1N1 | 0.927 | 0.639 |
| VirusSwine H3N2 | 4.150 | **0.044** |
| VirusWSN | -0.942 | 0.633 |
| **IL15** |  |  |
| (Intercept) | -0.322 | 0.816 |
| Time | 0.123 | 0.492 |
| VirusH3N2 | 0.295 | 0.854 |
| VirusH5N1 | 0.506 | 0.753 |
| ViruspH1N1 | 0.878 | 0.586 |
| VirusSwine H1N1 | 0.578 | 0.719 |
| VirusSwine H3N2 | 1.930 | 0.236 |
| VirusWSN | 0.562 | 0.727 |
| **IL17a** |  |  |
| (Intercept) | -0.494 | 0.302 |
| Time | 0.042 | 0.491 |
| VirusH3N2 | 0.016 | 0.977 |
| VirusH5N1 | -0.091 | 0.869 |
| ViruspH1N1 | 0.376 | 0.496 |
| VirusSwine H1N1 | 0.104 | 0.850 |
| VirusSwine H3N2 | 1.088 | 0.057 |
| VirusWSN | 0.103 | 0.852 |
| **IL1a** |  |  |
| (Intercept) | 2.633 | 0.050 |
| Time | -0.632 | **0.001** |
| VirusH3N2 | 5.869 | **0.001** |
| VirusH5N1 | 7.559 | **0.000** |
| ViruspH1N1 | 6.451 | **0.000** |
| VirusSwine H1N1 | 7.873 | **0.000** |
| VirusSwine H3N2 | 6.762 | **0.000** |
| VirusWSN | 6.570 | **0.000** |
| **IL1b** |  |  |
| (Intercept) | 0.660 | 0.607 |
| Time | -0.857 | **0.000** |
| VirusH3N2 | 4.540 | **0.005** |
| VirusH5N1 | 6.286 | **0.000** |
| ViruspH1N1 | 4.477 | **0.006** |
| VirusSwine H1N1 | 6.998 | **0.000** |
| VirusSwine H3N2 | 4.475 | **0.006** |
| VirusWSN | 4.385 | **0.007** |
| **IL1ra** |  |  |
| (Intercept) | 1.826 | 0.389 |
| Time | -0.976 | **0.001** |
| VirusH3N2 | 9.414 | **0.001** |
| VirusH5N1 | 10.605 | **0.000** |
| ViruspH1N1 | 10.998 | **0.000** |
| VirusSwine H1N1 | 11.340 | **0.000** |
| VirusSwine H3N2 | 7.671 | **0.004** |
| VirusWSN | 10.228 | **0.000** |
| **IL2** |  |  |
| (Intercept) | -0.889 | 0.401 |
| Time | -0.173 | 0.209 |
| VirusH3N2 | 0.620 | 0.612 |
| VirusH5N1 | 0.704 | 0.565 |
| ViruspH1N1 | 0.716 | 0.559 |
| VirusSwine H1N1 | 0.865 | 0.481 |
| VirusSwine H3N2 | 3.733 | **0.005** |
| VirusWSN | 0.592 | 0.628 |
| **IL3** |  |  |
| (Intercept) | -1.377 | 0.303 |
| Time | -0.127 | 0.457 |
| VirusH3N2 | 1.593 | 0.305 |
| VirusH5N1 | 1.734 | 0.265 |
| ViruspH1N1 | 2.529 | 0.109 |
| VirusSwine H1N1 | 0.615 | 0.689 |
| VirusSwine H3N2 | 1.227 | 0.427 |
| VirusWSN | 2.180 | 0.165 |
| **IL4** |  |  |
| (Intercept) | 1.678 | 0.191 |
| Time | 0.078 | 0.633 |
| VirusH3N2 | -0.316 | 0.829 |
| VirusH5N1 | 1.445 | 0.329 |
| ViruspH1N1 | -0.460 | 0.754 |
| VirusSwine H1N1 | 2.129 | 0.155 |
| VirusSwine H3N2 | 0.761 | 0.605 |
| VirusWSN | -0.055 | 0.970 |
| **IL5** |  |  |
| (Intercept) | 0.202 | 0.765 |
| Time | 0.090 | 0.307 |
| VirusH3N2 | -0.268 | 0.733 |
| VirusH5N1 | -0.249 | 0.752 |
| ViruspH1N1 | -0.248 | 0.752 |
| VirusSwine H1N1 | -0.118 | 0.880 |
| VirusSwine H3N2 | 2.052 | **0.014** |
| VirusWSN | -0.263 | 0.738 |
| **IL6** |  |  |
| (Intercept) | -1.377 | 0.445 |
| Time | -0.185 | 0.428 |
| VirusH3N2 | 7.602 | **0.001** |
| VirusH5N1 | 10.051 | **0.000** |
| ViruspH1N1 | 8.207 | **0.001** |
| VirusSwine H1N1 | 10.129 | **0.000** |
| VirusSwine H3N2 | 7.892 | **0.001** |
| VirusWSN | 7.660 | **0.001** |
| **IL7** |  |  |
| (Intercept) | 1.678 | 0.282 |
| Time | -0.329 | 0.108 |
| VirusH3N2 | 1.277 | 0.478 |
| VirusH5N1 | 2.707 | 0.140 |
| ViruspH1N1 | 1.664 | 0.357 |
| VirusSwine H1N1 | 2.725 | 0.137 |
| VirusSwine H3N2 | 5.243 | **0.007** |
| VirusWSN | 1.164 | 0.517 |
| **IL8** |  |  |
| (Intercept) | 0.433 | 0.803 |
| Time | 0.120 | 0.593 |
| VirusH3N2 | 8.112 | **0.000** |
| VirusH5N1 | 8.938 | **0.000** |
| ViruspH1N1 | 9.435 | **0.000** |
| VirusSwine H1N1 | 8.914 | **0.000** |
| VirusSwine H3N2 | 7.284 | **0.001** |
| VirusWSN | 7.758 | **0.001** |
| **IL9** |  |  |
| (Intercept) | -0.434 | 0.603 |
| Time | -0.146 | 0.183 |
| VirusH3N2 | 0.519 | 0.593 |
| VirusH5N1 | 0.808 | 0.407 |
| ViruspH1N1 | 0.724 | 0.458 |
| VirusSwine H1N1 | 1.033 | 0.292 |
| VirusSwine H3N2 | 6.821 | **0.000** |
| VirusWSN | 0.592 | 0.542 |
| **IP10** |  |  |
| (Intercept) | 0.996 | 0.750 |
| Time | 0.213 | 0.597 |
| VirusH3N2 | 5.692 | 0.126 |
| VirusH5N1 | 10.218 | **0.009** |
| ViruspH1N1 | 6.143 | 0.100 |
| VirusSwine H1N1 | 9.783 | **0.012** |
| VirusSwine H3N2 | 7.538 | **0.047** |
| VirusWSN | 6.678 | 0.075 |
| **MCP1** |  |  |
| (Intercept) | 0.872 | 0.743 |
| Time | -0.142 | 0.679 |
| VirusH3N2 | 4.707 | 0.136 |
| VirusH5N1 | 2.181 | 0.482 |
| ViruspH1N1 | 4.671 | 0.139 |
| VirusSwine H1N1 | 2.324 | 0.454 |
| VirusSwine H3N2 | 7.036 | **0.030** |
| VirusWSN | 1.633 | 0.597 |
| **MCP3** |  |  |
| (Intercept) | 1.678 | **0.000** |
| Time | **0.009** | 0.748 |
| VirusH3N2 | -0.027 | 0.914 |
| VirusH5N1 | -0.099 | 0.693 |
| ViruspH1N1 | -0.027 | 0.914 |
| VirusSwine H1N1 | **0.034** | 0.891 |
| VirusSwine H3N2 | -0.311 | 0.220 |
| VirusWSN | -0.027 | 0.914 |
| **MDC** |  |  |
| (Intercept) | 1.678 | 0.198 |
| Time | 0.215 | 0.202 |
| VirusH3N2 | -0.698 | 0.640 |
| VirusH5N1 | 0.243 | 0.870 |
| ViruspH1N1 | -0.487 | 0.744 |
| VirusSwine H1N1 | 1.714 | 0.256 |
| VirusSwine H3N2 | 0.572 | 0.701 |
| VirusWSN | -0.567 | 0.704 |
| **MIP1a** |  |  |
| (Intercept) | 1.678 | **0.028** |
| Time | -0.149 | 0.121 |
| VirusH3N2 | -0.003 | 0.998 |
| VirusH5N1 | 0.469 | 0.578 |
| ViruspH1N1 | -0.100 | 0.905 |
| VirusSwine H1N1 | 0.841 | 0.323 |
| VirusSwine H3N2 | 0.507 | 0.548 |
| VirusWSN | 0.116 | 0.890 |
| **MIP1b** |  |  |
| (Intercept) | 1.678 | 0.059 |
| Time | -0.237 | **0.040** |
| VirusH3N2 | 0.344 | 0.729 |
| VirusH5N1 | 1.586 | 0.120 |
| ViruspH1N1 | 0.509 | 0.609 |
| VirusSwine H1N1 | 1.741 | 0.089 |
| VirusSwine H3N2 | 0.944 | 0.346 |
| VirusWSN | 0.834 | 0.404 |
| **PDGFAA** |  |  |
| (Intercept) | 1.325 | 0.649 |
| Time | 0.227 | 0.547 |
| VirusH3N2 | 1.502 | 0.657 |
| VirusH5N1 | 5.253 | 0.129 |
| ViruspH1N1 | 2.333 | 0.492 |
| VirusSwine H1N1 | 6.187 | 0.077 |
| VirusSwine H3N2 | 2.319 | 0.494 |
| VirusWSN | 3.365 | 0.324 |
| **PDGFAB_BB** |  |  |
| (Intercept) | 1.081 | 0.688 |
| Time | **0.026** | 0.941 |
| VirusH3N2 | 2.027 | 0.518 |
| VirusH5N1 | 3.342 | 0.291 |
| ViruspH1N1 | 0.106 | 0.973 |
| VirusSwine H1N1 | 2.951 | 0.350 |
| VirusSwine H3N2 | 2.115 | 0.501 |
| VirusWSN | 0.369 | 0.906 |
| **RANTES** |  |  |
| (Intercept) | -0.029 | 0.992 |
| Time | 0.256 | 0.512 |
| VirusH3N2 | 2.515 | 0.475 |
| VirusH5N1 | 6.380 | 0.078 |
| ViruspH1N1 | 3.342 | 0.345 |
| VirusSwine H1N1 | 6.602 | 0.069 |
| VirusSwine H3N2 | 3.502 | 0.322 |
| VirusWSN | 5.116 | 0.153 |
| **sCD40L** |  |  |
| (Intercept) | 1.678 | **0.000** |
| Time | -0.029 | 0.388 |
| VirusH3N2 | 0.088 | 0.772 |
| VirusH5N1 | 0.088 | 0.772 |
| ViruspH1N1 | 0.088 | 0.772 |
| VirusSwine H1N1 | -0.176 | 0.564 |
| VirusSwine H3N2 | -0.614 | 0.053 |
| VirusWSN | 0.088 | 0.772 |
| **TGFa** |  |  |
| (Intercept) | -0.358 | 0.786 |
| Time | 0.326 | 0.066 |
| VirusH3N2 | 3.156 | **0.049** |
| VirusH5N1 | 4.260 | **0.010** |
| ViruspH1N1 | 3.560 | **0.028** |
| VirusSwine H1N1 | 3.500 | **0.030** |
| VirusSwine H3N2 | 2.655 | 0.094 |
| VirusWSN | 3.918 | **0.017** |
| **TNFa** |  |  |
| (Intercept) | 0.091 | 0.964 |
| Time | -0.276 | 0.293 |
| VirusH3N2 | 2.983 | 0.208 |
| VirusH5N1 | 4.848 | **0.047** |
| ViruspH1N1 | 3.610 | 0.131 |
| VirusSwine H1N1 | 5.153 | **0.035** |
| VirusSwine H3N2 | 4.731 | 0.052 |
| VirusWSN | 3.607 | 0.131 |
| **TNFb** |  |  |
| (Intercept) | -0.889 | 0.549 |
| Time | -0.178 | 0.355 |
| VirusH3N2 | 0.623 | 0.717 |
| VirusH5N1 | 0.596 | 0.729 |
| ViruspH1N1 | 1.785 | 0.304 |
| VirusSwine H1N1 | 1.102 | 0.523 |
| VirusSwine H3N2 | 3.959 | **0.029** |
| VirusWSN | 0.617 | 0.720 |
| **VEGF** |  |  |
| (Intercept) | 1.425 | 0.481 |
| Time | 0.168 | 0.521 |
| VirusH3N2 | 1.514 | 0.519 |
| VirusH5N1 | 2.692 | 0.256 |
| ViruspH1N1 | 1.531 | 0.514 |
| VirusSwine H1N1 | 3.082 | 0.196 |
| VirusSwine H3N2 | 1.574 | 0.503 |
| VirusWSN | 1.115 | 0.634 |
